# Comprehensive Expression Analysis of microRNAs and mRNAs in Synovial Tissue from a Mouse Model of Early Post-Traumatic Osteoarthritis

**DOI:** 10.1038/s41598-017-17545-1

**Published:** 2017-12-18

**Authors:** Louise H. W. Kung, Varshini Ravi, Lynn Rowley, Katrina M. Bell, Christopher B. Little, John F. Bateman

**Affiliations:** 1 Murdoch Childrens Research Institute, Parkville, Victoria 3052 Australia; 2 Raymond Purves Bone and Joint Research Laboratories, Kolling Institute of Medical Research, Institute of Bone and Joint Research, University of Sydney, St Leonards, New South Wales, 2065 Australia; 3 Department of Biochemistry and Molecular Biology, University of Melbourne, Parkville, Victoria 3052 Australia

## Abstract

To better understand the molecular processes involved in driving osteoarthritis disease progression we characterized expression profiles of microRNAs (miRNA) and mRNAs in synovial tissue from a post-traumatic OA mouse model. OA was induced in 10–12 week old male C57BL6 mice by bilateral surgical destabilization of the medial meniscus (DMM). RNA isolated from the anterior synovium of mice at 1 and 6 weeks post-surgery was subject to expression profiling using Agilent microarrays and qPCR. OA severity was determined histologically. Anterior and posterior synovitis decreased with post-operative time after sham and DMM. No differences in synovitis parameters were evident between sham and DMM in the anterior synovium at either time. While expression profiling revealed 394 miRNAs were dysregulated between 1 and 6 week time-points in the anterior synovium, there were no significant changes in miRNA or mRNA expression between DMM and sham mice at both time-points. Bioinformatic analysis of the miRNAs and mRNAs differentially expressed in tandem with the resolution of anterior synovial inflammation revealed similar biological processes and functions, including organismal injury, connective tissue disorder and inflammatory responses. Our data demonstrates that early OA-specific patterns of synovial miRNAs or mRNAs dysregulation could not be identified in this model of post-traumatic OA.

## Introduction

Although primarily characterized by the progressive breakdown of articular cartilage, osteoarthritis (OA) is often accompanied with synovial hyperplasia and inflammation (synovitis), sclerosis of the underlying subchondral bone, osteophyte formation and ligament and meniscal damage, ultimately resulting in complete “organ” failure^1^. Evidently, all joint tissues, including the synovium contribute to the pathological process^2–7^. In particular synovitis is no longer thought of as a secondary process to cartilage degeneration, but rather one that actively contributes not only to increased OA joint pain and dysfunction but to both the initiation and progression of cartilage loss^8^. In patients a strong association exists not only between the presence and severity of synovitis and progression of existing OA^9–11^, but also with disease incidence^12^. Persistent elevation of cytokines and chemokines, and accumulation of lymphocytes and macrophages is a feature of the synovitis associated with joint injury and early post-traumatic (pt)OA in patients (reviewed in^13^). In mice, genetic modifications that specifically reduce the inflammatory response significantly reduce cartilage erosion in models of ptOA (reviewed in^6^). This cumulative clinical and pre-clinical data clearly demonstrates that specific aspects of synovial inflammation are viable therapeutic targets, however it is unclear whether the inflammatory pathways activated in ptOA are distinct from those in non-OA-inducing joint trauma. Any such “OA-specific” inflammatory events or pathways would be especially attractive for development of new therapies through minimizing off-target and side-effects. This highlights the critical need to perform parallel molecular studies on all OA-affected joint tissues to determine their pathophysiological contribution, and to compare with appropriate controls to define disease-specific mechanisms.

In this regard, microRNAs (miRNAs) are being identified as novel regulators of cartilage homeostasis and OA pathology, making them exciting candidates for treatment and as diagnostic biomarkers^14–18^. However, their role in synovial OA pathology and the disease-specificity of any such association has yet to be determined. MiRNAs are a family of evolutionarily conserved, small, non-coding RNAs, with over 2500 mature miRNAs identified in humans to date (www.mirbase.org)^19^. MiRNAs are important cellular regulators which act by modulating gene expression at the post-transcriptional level during various physiological and disease settings. Primary and precursor miRNAs are processed into mature miRNAs that range from 21–25 nucleotides in length. Mature miRNAs associate with Argonaute family proteins in an RNA-induced silencing complex (RISC) which are then guided to target mRNAs where base-pairing of the miRNA results in translational repression or mRNA decay of the target transcript^20–22^.

Investigators have demonstrated changes in miRNAs expression in the synovium and synovial fluid from OA and rheumatoid arthritis (RA) patients, suggesting a role of miRNAs in synovitis/inflammation^23–28^. Furthermore, miR-16, miR-146a, miR-155 and miR-223 in synovial fluid were identified as potential diagnostic biomarkers for distinguishing between RA and OA patients^25^. However, these studies have been limited by high biological/pathological variability between patients, and accessibility only to samples from end-stage disease, a therapeutically-redundant timeframe. Furthermore, with regard to the key question of whether synovial inflammation in ptOA is distinct from that seen in non-OA-inducing joint injury, clinical studies lack appropriate control samples.

Here, we aim to characterize the expression profile of miRNAs and their target mRNAs in synovium of normal, early and progressive stages of ptOA in a clinically reproducible mouse model (destabilization of the medial meniscus model; DMM). We conducted parallel global miRNA and mRNA expression microarrays of synovium from DMM (“OA-inducing injury”) and sham-operated (“non-OA-inducing injury”) joints at 1 and 6 weeks post-surgery. These studies provide the first comprehensive transcriptome-wide analysis of temporal and disease-specific regulation of synovial mRNAs and their regulatory miRNAs in ptOA.

## Results

### Histological evaluation of DMM-operated joints relative to sham

**Figure 1:**
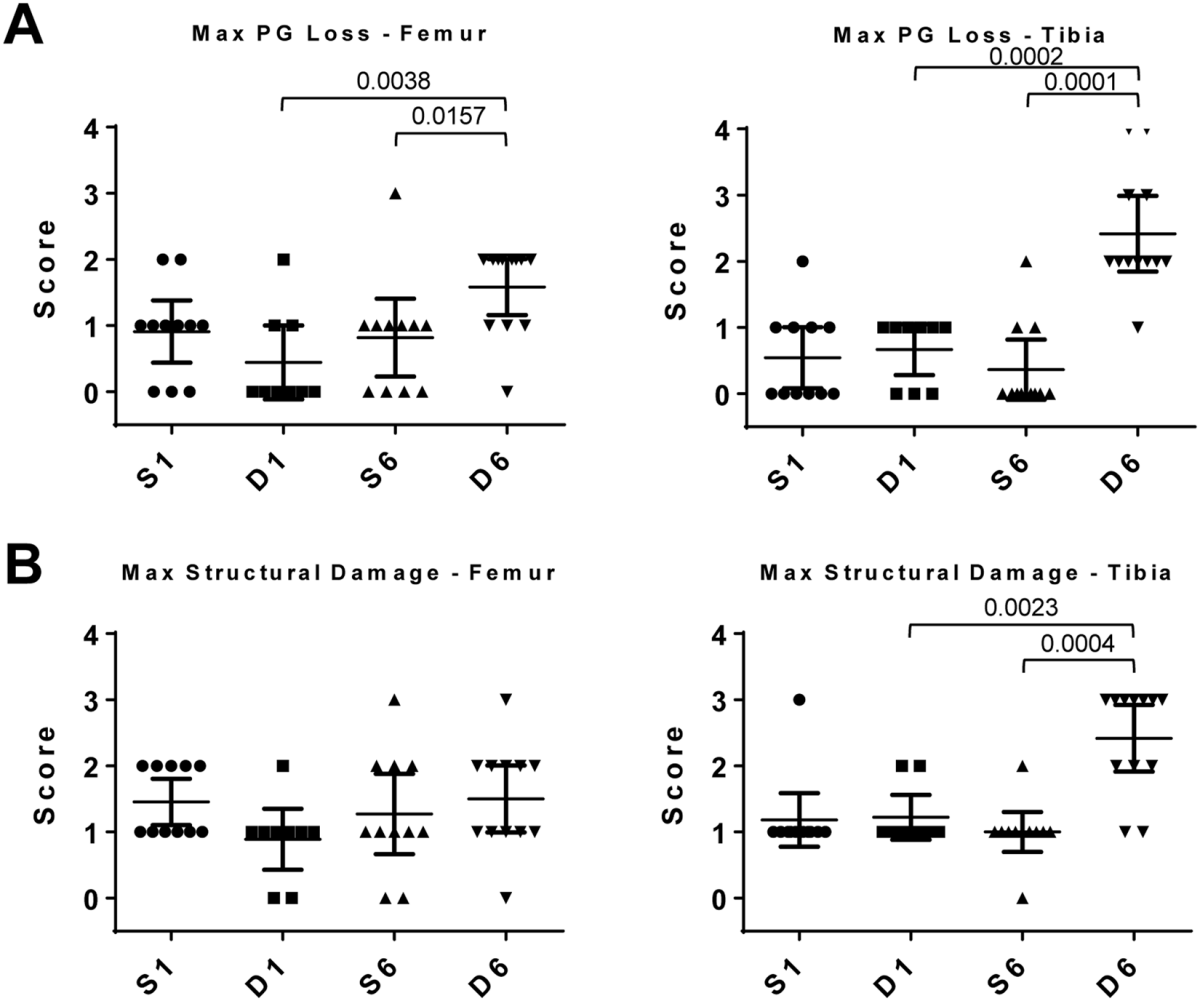
Histologic scores of maximal (**A**) proteoglycan (PG) loss and (**B**) cartilage structural damage in the femur and tibia of male mouse knee joints at 1 and 6 weeks post DMM and sham surgeries. n = 11 (S1), n = 9 (D1), n = 11 (S6), n = 12 (D6). Scatter plots display values for each mouse and mean (horizontal bar) ±95% confidence intervals. Significant differences between groups connected by lines with exact p-values for each comparison indicated above the line. S1 = 1 week Sham; D1 = 1 week DMM; S6 = 6 weeks Sham; D6 = 6 weeks DMM.

No statistically significant proteoglycan loss or cartilage structural damage was observed in DMM-operated mice compared with sham in either femur or tibia at week 1 (Fig. 1). By week 6, proteoglycan loss was significantly increased in both femur and tibia of DMM mice compared with sham and compared with week 1 DMM group (Fig. 1A). This was accompanied by a significant increase in cartilage structural damage in the tibia but not the femur of 6 week DMM mice compared with sham and compared with week 1 DMM group (Fig. 1B). This progressive increase in cartilage pathology with more severe damage in tibia compared with femur is similar to previous reports^29,30^.

**Figure 2:**
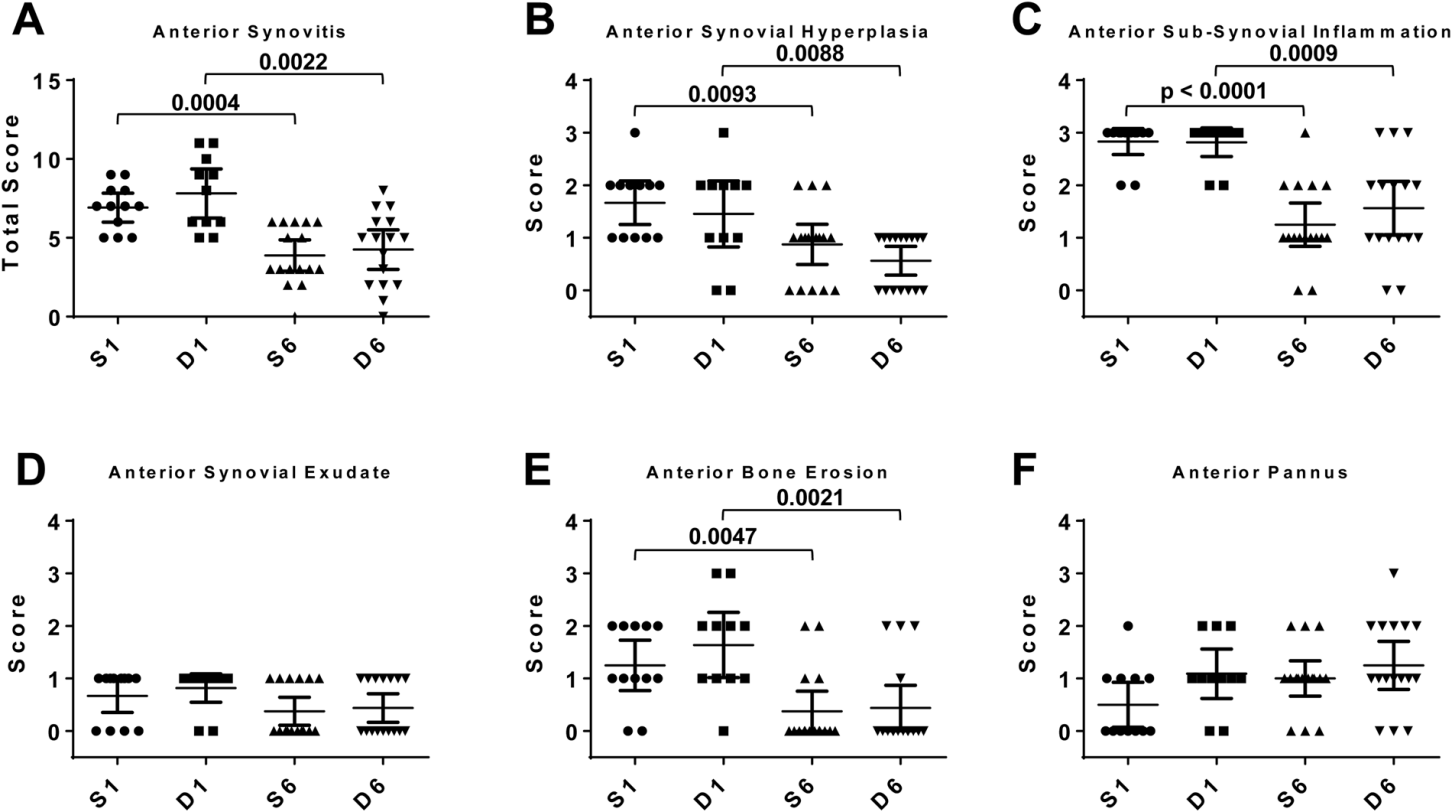
Histologic scoring examining synovitis parameters in the anterior region of male mouse knee joints: (**A**) synovitis, (**B**) synovial hyperplasia, (**C**) sub-synovial inflammation, (**D**) synovial exudate, (**E**) bone erosion and (**F**) pannus at 1 and 6 weeks post DMM and sham surgeries. n = 12 (S1), n = 11 (D1), n = 16 (S6), n = 16 (D6). Scatter plots display values for each mouse and mean (horizontal bar) ± 95% confidence intervals. Significant differences between groups connected by lines with exact p-values for each comparison indicated above the line. S1 = 1 week Sham; D1 = 1 week DMM; S6 = 6 weeks Sham; D6 = 6 weeks DMM.

The synovium was assessed for histologic signs of synovitis - synovial hyperplasia, sub-synovial inflammation, synovial exudate, bone erosion and pannus. Total anterior synovitis score decreased with post-operative time for both sham and DMM surgeries but there was no statistical difference between surgeries at either time-point (Fig. 2A). Most individual parameters (synovial hyperplasia, sub-synovial inflammation and bone erosion) showed a similar decline with time in both sham and DMM surgeries (Fig. 2B–E).

As previously described^31^ total synovitis scores in the posterior region of the joint distant from the surgical incision were lower than anterior at both time-points (Supplementary Fig. 2). This was largely driven by less sub-synovial inflammation and lining cell hyperplasia (compare Fig. 2 and Supplementary Fig. 2). As a result of being removed from the site of surgical inflammation, significant differences between sham and DMM were evident in the posterior synovium. Total synovitis was more severe in DMM compared with sham at 1 week (Supplementary Fig. 2A; p = 0.01). Of the individual parameters, synovial hyperplasia (week 6) and sub-synovial inflammation (week 1) were significantly increased in DMM joints compared with sham (Supplementary Fig. 2B,C). As with the anterior region, there was no difference between surgical interventions for exudate, bone erosion or pannus in the posterior joint. Temporal changes in synovial pathology were also evident in the posterior region, with declines in total synovitis in both sham and DMM mirroring those in the anterior region. Unlike hyperplasia in the anterior synovium, scores did not significantly decrease with post-operative time in the posterior region regardless of surgical intervention (Supplementary Fig. 2B).

### miRNA expression profiling of synovium tissue

We performed miRNA microarray analyses on 1 and 6 week post-sham and DMM synovium samples (n = 4) dissected from the anterior region of the joint. Of the 1,881 miRNAs represented on the microarrays, 559 miRNAs were detected above background.

**Figure 3:**
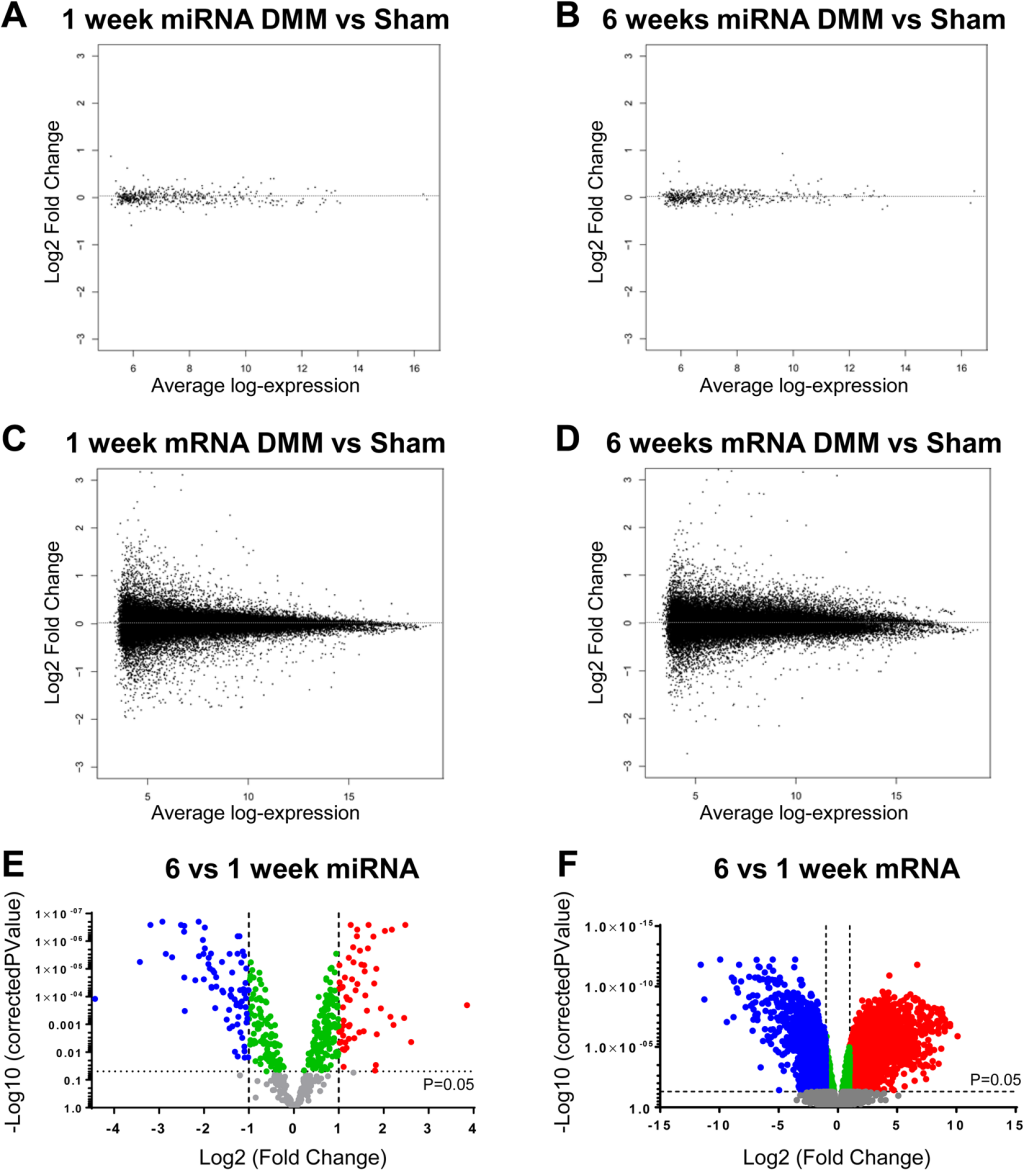
miRNA and mRNA microarray expression profiling of synovial tissue from male mice. MA plots displaying average log-expression intensity values and log2 fold change for each miRNA expressed between DMM and sham in (**A**) 1 week synovium and (**B**) 6 week synovium. MA plots displaying average log-expression intensity values and log2 fold change for each mRNA transcript expressed between DMM and sham in (**C**) 1 week synovium and (**D**) 6 week synovium. Volcano plots showing differential (**E**) miRNA expression and (**F**) mRNA expression in synovium of 6 week compared with 1 week post-surgery samples. Blue dots represent miRNA/mRNA down regulated >2 fold with adjusted p value < 0.05, green dots represent miRNA/mRNA with adjusted p value < 0.05 and log2 fold change between -1 and 1, red dots represent miRNA/mRNA up regulated >2 fold and adjusted p value < 0.05 and grey dots represent non-significant entities.

Following stringent analysis, we did not observe any statistically significant differences (adj.p.value < 0.05) in miRNA expression between DMM and sham surgeries at 1 or 6 week time-points (Fig. 3A,B). The expression levels of the miRNAs detected in our synovium samples were similar between DMM and sham surgeries, with no statistically significant dysregulation between the two groups at either time-point (Supplementary Tables 2 and 3).

**Figure 4:**
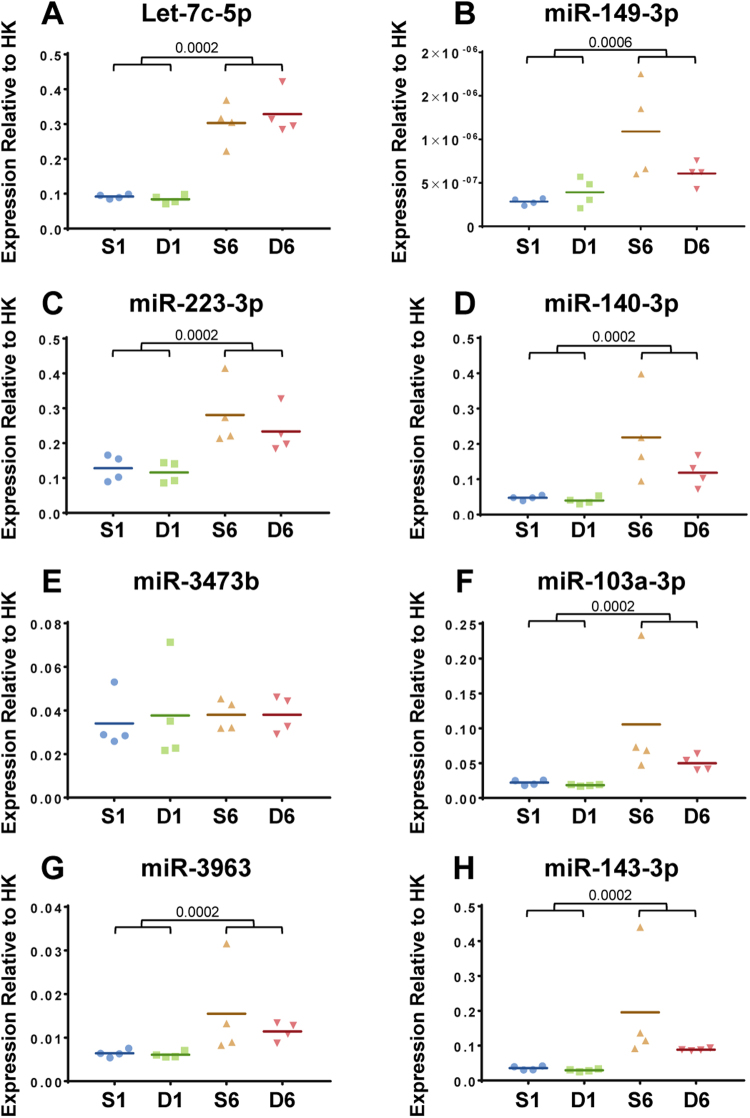
qPCR validation of miRNA expression in synovial tissue from male mice at 1 and 6 weeks post sham or DMM surgery was determined. Data shown as expression relative to the average expression of two housekeepers (HK), U6 and 5s. In all cases there was no significant differences between DMM and sham within each time point. Each symbol represents an individual mouse (average of 2 technical replicates). Horizontal bars show the mean expression. Significant differences as determined by unpaired Mann-Whitney U test between groups connected by lines with exact p-values for each comparison indicated over the line. S1 = 1 week Sham; D1 = 1 week DMM; S6 = 6 weeks Sham; D6 = 6 weeks DMM.

As a measure of the sensitivity and confidence of the microarray approach to detect statistically significant changes in miRNA expression, analysis did demonstrate statistically significant miRNA dysregulation with post-operative time in synovium tissues, thus reflecting the temporal histologic changes in the anterior synovium (Fig. 2). 394 miRNAs were dysregulated between 1 and 6 week time-points (adj.p.value < 0.05; Supplementary Table 4). 63 miRNAs were up-regulated >2 fold (adj.p.value < 0.05) and 77 miRNAs were down-regulated >2 fold (adj.p.value < 0.05) in 6 week compared to 1 week samples (Fig. 3E; Supplementary Table 4). The miRNA array data was independently verified by qPCR of a selection of miRNAs in additional biological replicates (Fig. 4).

### mRNA expression profiling of synovium tissue

Similar to the miRNA data, mRNA expression also differed with post-operative time in anterior synovium tissues, again demonstrating the sensitivity of the arrays to detect statistically significant changes. mRNA expression profiling demonstrated >12,000 probes to be differentially expressed between 1 and 6 week time-points (adj.p.value < 0.05). 5,335 probes were up-regulated >2 fold (adj.p.value < 0.05) and 3,033 probes were down regulated >2 fold (adj.p.value < 0.05) in 6 week compared to 1 week samples (Fig. 3F; Supplementary Table 5). The full dataset can be found in the GEO data repository (at http://www.ncbi.nlm.nih.gov/geo/; accession number GSE99738).

**Figure 5:**
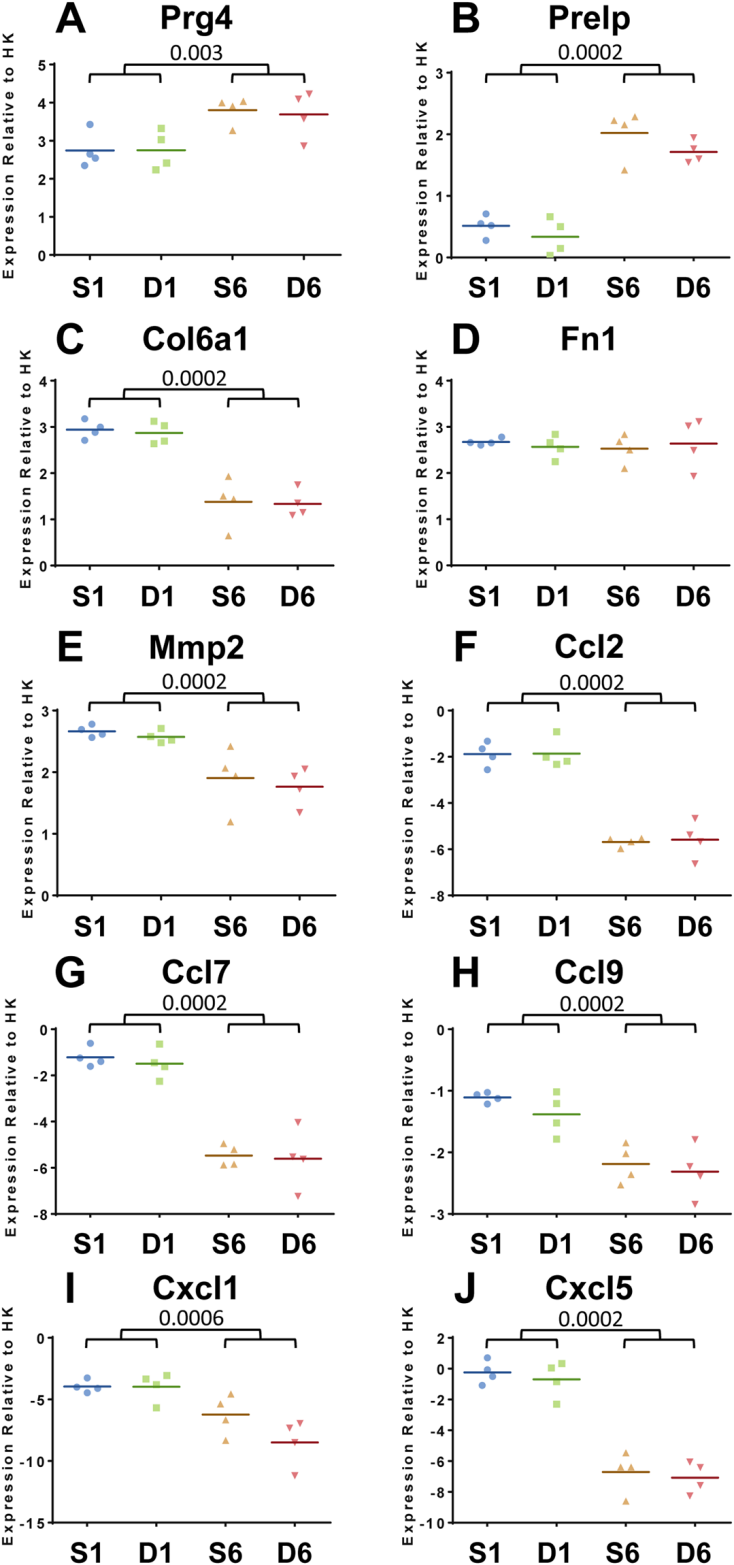
qPCR validation of gene expression in synovial tissue from male mice at 1 and 6 weeks post sham or DMM surgery was determined. Data shown as expression relative to the average expression of two housekeeping genes (HK), *Atp5b* and *Rpl10*. Each symbol represents an individual mouse (average of 2 technical replicates). In all cases there was no significant differences between DMM and sham at either time point. Horizontal bars show the mean expression. Significant differences as determined by unpaired Mann-Whitney U test between groups connected by lines with exact p-values for each comparison indicated over the line. S1 = 1 week Sham; D1 = 1 week DMM; S6 = 6 weeks Sham; D6 = 6 weeks DMM.

Mirroring the miRNA expression data, there were no statistically significant changes in mRNA expression between DMM and sham synovium samples at 1 or 6 week time-points. Despite lack of statistical significance, a cohort of mRNAs were dysregulated >2 fold in DMM compared with sham (Fig. 3C,D; Supplementary Tables 6 and 7). At 1 week, 175 and 205 probes were >2 fold up-regulated or down-regulated in DMM compared with sham, respectively (Supplementary Table 6). At 6 weeks, 145 and 116 probes were >2 fold up-regulated or down-regulated in DMM compared with sham, respectively (Supplementary Table 7). Of those, a number of chemokines (e.g. *Ccl2*, *Ccl7, Cxcl1, Cxcl5*), along with troponin and myosin genes, were >2 fold dysregulated in the DMM group compared with sham (Supplementary Table 7). However, independent verification by qPCR of a selection of these genes (Fig. 5) confirmed no statistically significant changes in gene expression were observed in DMM versus sham comparisons. As with the microarray data, significant changes with post-operative time were confirmed for most of the genes examined.

### Dysregulated molecular functions and pathways associated with resolution of synovial inflammation

**Figure 6:**
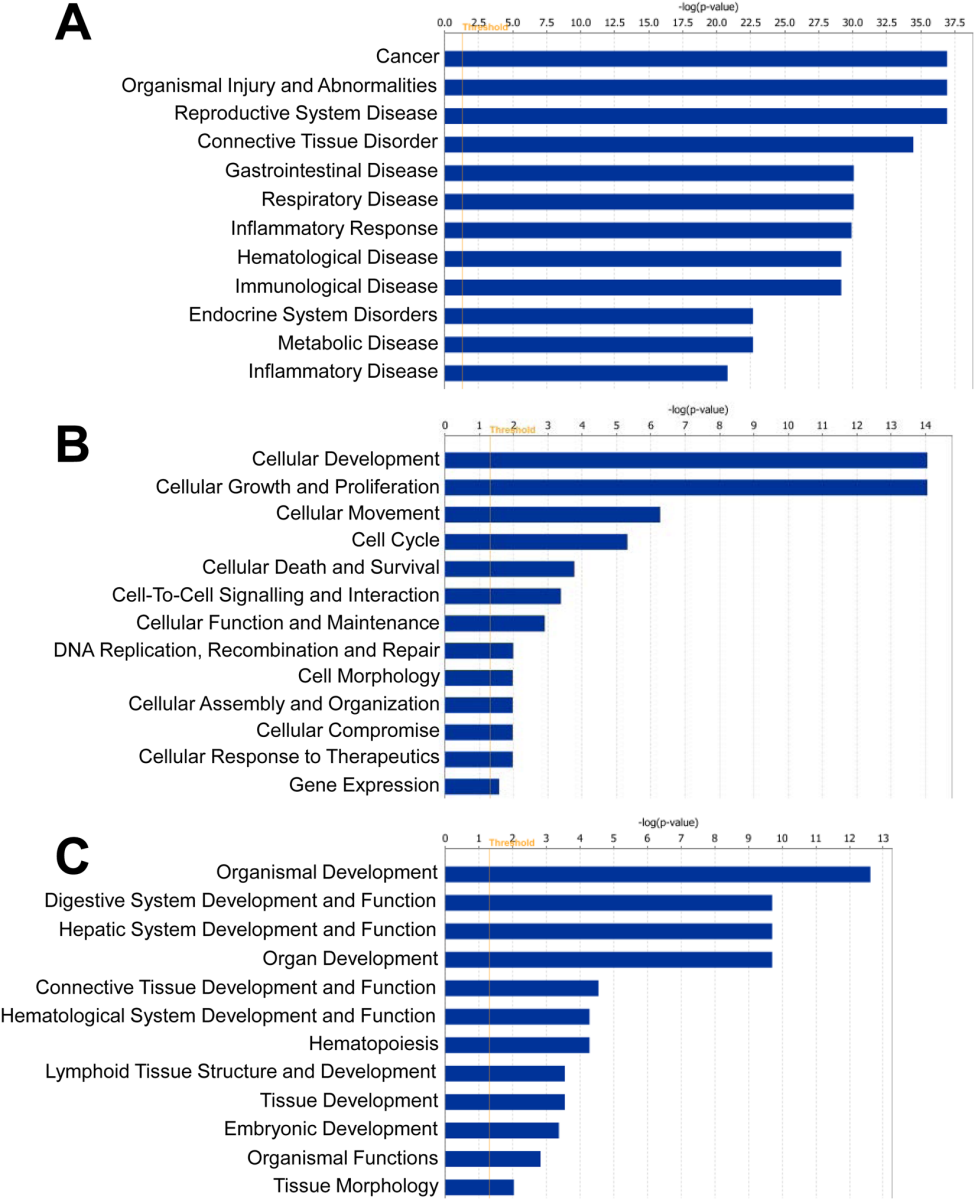
Ingenuity pathway-generated core functional analysis of the 394 differentially expressed synovial miRNAs between 1 and 6 weeks post-surgery (adj.p.val < 0.05). Most significant (**A**) disease and disorders, (**B**) molecular and cellular functions and (**C**) physiological system development and function mapped to the network of dysregulated miRNAs. Threshold bar shows cut-off point of significance p < 0.05, −log(P-value) of 1.3 using Fisher’s exact test.

**Figure 7:**
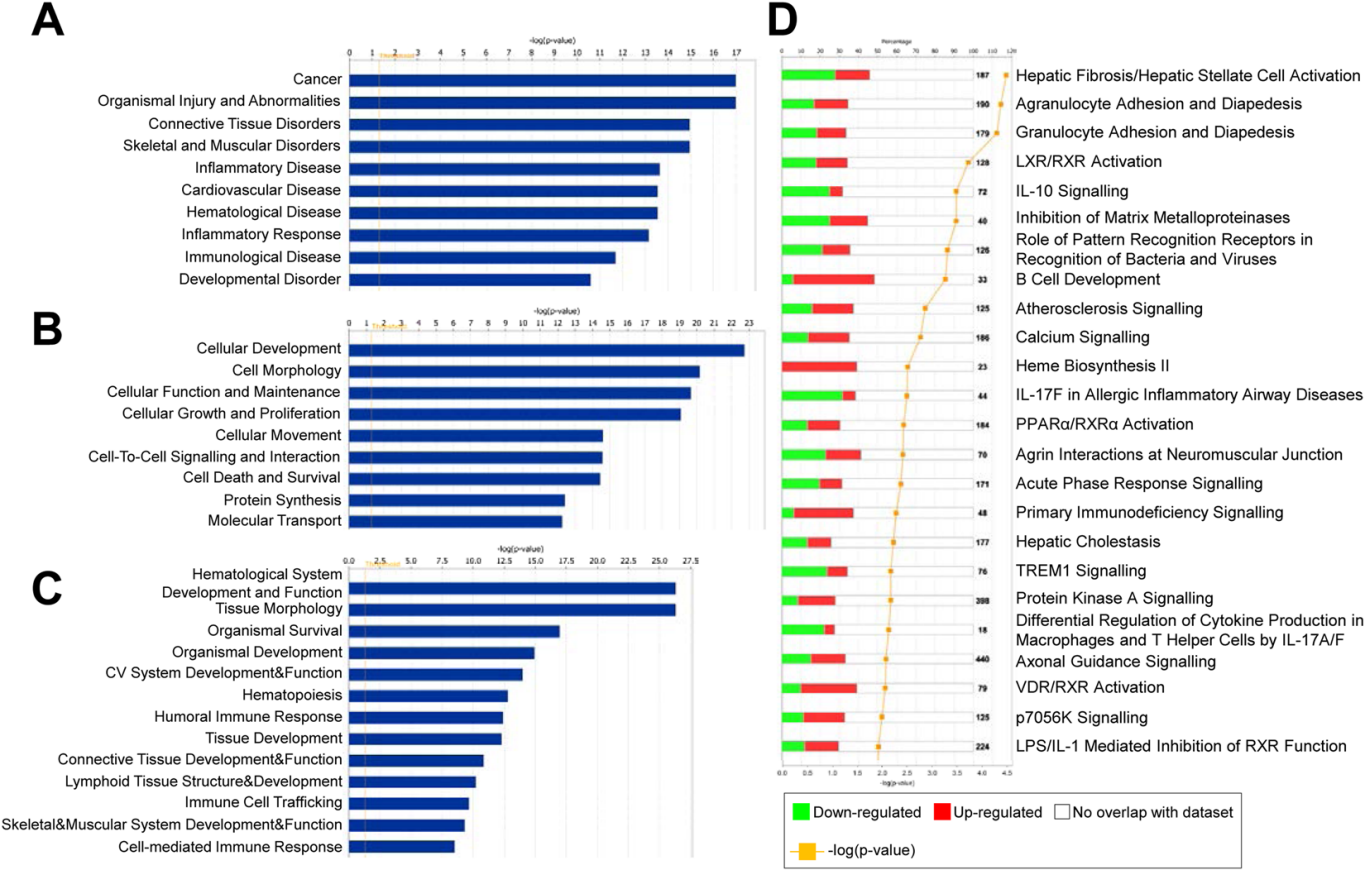
Ingenuity pathway-generated core functional analysis of the top 1000 differentially expressed genes between 1 and 6 weeks post-surgery (adj.p.val < 0.05). Most significant (**A**) disease and disorders, (**B**) molecular and cellular functions, (**C**) physiological system development and function and (**D**) canonical pathways mapped to the network of dysregulated genes. Threshold bar shows cut-off point of significance p < 0.05, −log(P-value) of 1.3 using Fisher’s exact test.

To explore the biological function of the miRNAs and mRNAs that were significantly dysregulated (adj.p.val. < 0.05) with post-operative time, we employed a bioinformatics approach using Ingenuity Pathway Analysis to identify functional networks and pathways that were altered. Core functional analysis revealed “connective tissue disorders”, “organismal injury abnormalities” and “inflammatory disease and response” as common top diseases and disorders of the differentially expressed miRNAs and mRNAs between 1 and 6 week post-surgery (Figs 6 and 7A). Likewise, “connective tissue development and function” and physiological cellular processes, such as, “proliferation”, “death”, “survival”, “signaling interactions” and “hematological processes” were significantly affected by post-operative time at the miRNA and mRNA level (Figs 6 and 7B,C). Clustering of differentially expressed genes into canonical pathways showed dysregulation in those implicated in fibrosis and cell activation, agranulocyte and granulocyte adhesion, LXR/RXR activation, IL-10 signalling, and inhibition of matrix metalloproteinases (Fig. 7D).

## Discussion

The pathophysiology of OA has broadened from primarily considered a cartilage-associated disorder to a whole joint disease, however, the relative contributions of each joint tissue to the overall pathology remains to be elucidated. The purpose of this study was to explore the expression profiles of miRNAs and mRNAs in a mouse a model of early OA in order to identify OA-dysregulated miRNAs and mRNAs in inflamed synovial tissues.

As it is impossible to obtain human synovial tissue to study the initiation and early stages of OA, we used a clinically reproducible animal model of ptOA (DMM) to overcome these limitations and gain insight into the pathogenesis of early OA. Our previous analysis demonstrated not only progressive cartilage and bone pathology mimicking human OA in mouse joints following DMM, but also significant synovitis over and above that seen in sham-operated joints^31^. In this previous study significantly greater synovitis was seen in the anterior aspect of DMM compared with sham at weeks 4–8 but not week 1, and at both times posteriorly. In the current study posterior synovitis was similarly greater in DMM than sham at both 1 and 6 weeks, however anterior synovitis was not significantly different between surgeries. This subtle dissimilarity between experiments may be a statistical reflection of biological variability, or it may reflect differences associated with bilateral versus unilateral surgery or between surgeons, although both are very experienced (>500 DMM each) and one (CBL) was common to both studies and trained the other so techniques are similar. Importantly, the current study confirms greater overall joint inflammation with the onset and progression of post-surgical OA pathology in DMM compared with that induced by the arthrotomy alone^31^. Although synovial tissue can only be successfully isolated from the anterior aspect of the joint, we have previously shown that the underlying molecular activation can be significantly different despite equivalent histopathologic synovitis^31^. Thus it seems unlikely that the lack of OA-specific RNA synovitis signature in the current study is due to the inter-experimental variability in anterior synovitis.

Our miRNA expression profiling of anterior synovium showed no significant miRNA expression differences between DMM and sham at either 1 week or 6 weeks post-surgery. Similarly, mRNA expression profiling also revealed no statistically significant OA-related changes in mRNA expression. These data suggest that while synovitis is undoubtedly critical in the pathophysiology of ptOA initiation and early progression in this model^6^, there was no OA-specific miRNA nor mRNA dysregulation in the synovium associated with the joint inflammation. A facile explanation for the lack of significant OA-specific differences could be that the expression arrays lacked sensitivity. However, this was discounted by the statistically significant dynamic changes in miRNA and mRNA expression between time-points, consistent with the temporal changes observed in the pathology of both the anterior and posterior synovium. This clearly demonstrates a significant association between synovitis severity and miRNA/mRNA expression changes, and the sensitivity of the microarrays to detect these if present and thus the robustness of the datasets. Our previous analyses using a similar array approach identified 725 and 1,293 significantly differentially regulated mRNAs in cartilage of sham versus DMM joints at the same 1 and 6 week time-points, respectively^32^.

While “OA-specific” synovial miRNA:mRNA targeting did not occur, the inflammation associated with the joint injury (surgery) plays a critical role in concert with joint instability (DMM) to induce ptOA. For instance, we know from previous studies using knockout mice that DMM-induced OA can be reduced by genetically targeting specific aspects of inflammation (e.g. CCR5, PAR2, IL-1, IL-6)^31,33–35^. Furthermore, since sham surgery does not induce OA itself and our current data shows common signatures between OA-associated (DMM) and regular wound healing (sham) synovial inflammation, this implies a combination of inflammation and altered biomechanics (instability, increased focal loading) are required for OA-initiation. Our studies demonstrate that there is no unique OA-inducing synovial inflammatory driver in early surgically-induced ptOA, and in order to dissect out the interaction between inflammation and mechanical instability the use of a non-surgical OA model^36^ would be beneficial in future studies.

While not specific to OA-inducing injury, the temporal miRNA and mRNA changes associated with resolution of synovial inflammation may nevertheless identify potential disease-modifying therapeutic targets. Bioinformatic analysis of the miRNAs and mRNAs differentially expressed in tandem with the resolution of synovial inflammation revealed similar biological processes and functions, such as, connective tissue disorders, organismal injury and inflammatory disease and responses. Canonical pathway analysis on the dysregulated mRNAs also revealed biologically relevant processes associated with synovial tissue changes in OA e.g. fibrosis and cell activation. An important caveat to be aware of when utilizing bioinformatics tools like IPA is the heavy population of data from cancer and non-musculoskeletal studies which may inflict bias in the downstream analysis of our data.

As noted above a limitation of this study is related to an unavoidable synovial sampling/dissection issue. Although less severe, the most profound pathological differences between sham and DMM in the synovium were in the posterior region of the joint (Supplementary Fig. 2). Due to its anatomical location we were unable to isolate the posterior synovial tissue by gross microscopic dissection, and therefore unable to confirm if the histological differences were reflected at the miRNA/mRNA expression level. This technical issue is a challenge that many in the field face, and may require use of laser capture micro-dissection as previously done for articular cartilage in this model^32^.

Comparative gene expression analysis of normal/reactive versus inflamed areas of synovial membrane from individual patients revealed 896 genes that were dysregulated between the two areas reflecting the focal nature of the synovial response in OA^37^. A large number of inflammatory mediators were up-regulated in the inflamed synovial samples, including CXCL1, CXCL2 and CXCL5^37^. Interestingly, CXCL1, CXCL5 and CCL7 were dysregulated >2 fold in 6 week DMM compared with sham in the current study, albeit not significantly (Supplementary Table 7), suggesting their likely involvement in synovial inflammation as OA joint pathology is initiated and progresses. The focal nature of the mRNA dysregulation seen in human OA joints^37^, highlights a limitation of the current study where RNA was isolated from the entire anterior synovium and infra-patella fat pad. It may be that it is only focal regions in this tissue and/or a particular population of cells such as macrophages, synoviocytes, T-lymphocytes that were specifically affected by OA. Such subtle changes may be masked in the whole tissue extracts, and cell sorting (FACs analysis) of tissue digests^31^ and assaying changes in miRNA and mRNA of individual cell types could be beneficial in identifying OA-specific changes.

Overall, miRNAs in OA synovium have not been extensively studied mechanistically, however, numerous studies have illustrated changes in miRNA expression in synovial tissue and fluid from end-stage rheumatoid arthritis (RA) patients^23–26,38^, thus indicating a potential role of miRNAs in RA-specific synovitis/inflammation. For instance, the dysregulation of miR-146 in RA has been documented by several investigators^23,38,39^ and has been shown to play a role in balancing inflammatory responses in cartilage and synovium, as well as pain-related functions in glial cells^40^. From a biomarker perspective, profiling synovial fluid circulating locally within the joint cavity may offer an indication of pathology changes occurring during knee OA initiation and progression. Li *et al*. demonstrated distinct miRNA signatures in the synovial fluid between early and late-stage knee OA patients, with the identified miRNAs likely to be released from synovial tissue following inflammatory (IL-1β) stimulation^28^. However, these studies lacked appropriate “non-arthritis inducing” joint controls which our study includes.

Furthermore, Genemaras *et al*. showed that miRNA expression and extracellular release by synoviocytes following cytokine stimulation, is time-dependent^41^. It is therefore possible that OA-specific synovial miRNA and mRNA signatures would be apparent at different times than those examined in the current study. This may be particularly true in a surgical model such as DMM where our data suggests it is likely that at the early time-points, synovial changes are driven primarily by responses to the surgical injury. Differences between DMM and sham joints in synovial pathology scores became apparent as inflammation in sham-operated joints resolved more rapidly and returned to levels of a naïve joint^31^. Further array analysis at additional time-points after surgery might allow us to distinguish between responses related to surgical injury/wound healing and those pathologically associated with OA. Several studies have shown gene expression changes in synovial samples from OA patients undergoing total knee replacement surgery or other arthroscopic procedures^37,42,43^. However analyses of late stage disease in mice offer little advantage over similar studies of advanced human OA, in terms of defining potential therapeutic targets to stop or slow disease onset or progression.

In conclusion, the results of the current study suggest that, although the DMM mouse model of OA depicted typical pathological features of the human disease, early OA-specific patterns of synovial miRNA or mRNA dysregulation could not be identified. Further investigations are required to shed light into the pathological contributions of miRNAs in synovial inflammation in OA that occurs prior to irreversible cartilage damage and the requirement of total knee replacement surgery. Similarly, the molecular role of miRNAs and their influence in the OA pathology of other joint tissues, such as the cartilage and subchondral bone, remains to be determined.

## Materials and Methods

### Animal Model of OA

All animal experiments were approved by the Royal North Shore Hospital Animal Ethics Committee (Protocol. RESP/14/77) and conducted in accordance with the Australian Code for the Care and Use of Animals for Scientific Purposes (National Health and Medical Research Council). Thirty two 10–12 week old male C57BL/6 mice were used for this study. Sixteen animals had bilateral DMM surgery, as described previously^30,31,44^. Briefly, under isoflurane anaesthesia and following standard surgical site preparation and isolation with sterile paper drapes, the medial menisco-tibial ligament was exposed by medial para-patellar arthrotomy and infrapatellar fat pad elevation, and transected with curved dissecting forceps by a single experienced specialist veterinary surgeon (CBL) using sterile surgical techniques. Joints were flushed with sterile saline prior to separate closure of the joint capsule, subcutaneous tissue (8/0 polyglactin 910 suture) and skin (cyanoacrylate). Bilateral sham-operations were performed in 16 age- and sex-matched mice, where all procedures were identical except the medial menisco-tibial ligament was visualised but not transected. Mice were randomly allocated to groups/harvest-time-points using their individual ID-numbers prior to study commencement. DMM/Sham mice were co-housed 2–5 animals/30 × 20 × 18 cm individually-ventilated-cage with filter lids, provided with sterilised bedding and environmental enrichment, maintained at 21–22 °C with a 12-hour light/dark cycle, and received water and complete pelleted food *ad libitum*. Mice received no post-arthritis-induction medication and were allowed unrestricted cage exercise. Animals were sacrificed at 1 and 6 weeks after surgery. From each animal, one randomly selected joint was processed for histology whilst the other was used for expression analysis. A cohort of n = 4/time-point/treatment was used for the microarray experiment and a separate cohort of n = 4/time-point/treatment was used for qPCR validation.

### Histopathological Analysis of OA Features

Knee joints were fixed, decalcified, processed for histology and scored for OA histopathologic features as described previously^30,31^. Sagittal sections every 80 µm across the medial femoro-tibial joint were collected and stained with toluidine blue-fast green. Histopathologic features were scored by a single experienced observer (VR) blinded to surgical intervention and post-operative time as recommended^45^. Score reliability was ensured through use of a standardised protocol with clearly defined parameters, and confirmed in a random selection of slides by a second independent blinded observer (CBL). Histologic scores for cartilage proteoglycan loss (0–5) and structural damage (0–8) in both the femur and tibia were scored on 11 serial sections/knee to determine the maximum medial tibial and femoral pathology in each joint. Synovitis was assessed in a single section adjacent to the intercondylar fossa as previously described^31^. Briefly, anterior and posterior joint regions were evaluated separately for the presence and severity of synovial hyperplasia (score range 0–3), sub-synovial cell infiltration/inflammation (score range 0–3), synovial exudate (score range 0–1), cortical bone erosion (score range 0–3) and pannus (score range 0–3). Total anterior and posterior synovitis scores were computed by addition of the scores for each parameter in the relevant joint region in each mouse. Anterior and posterior scores were kept separate as only the former is collected for RNA extraction (see below).

### Synovium tissue dissection and RNA preparation

The anterior synovial tissues (comprising the joint capsule, synovial lining, infrapatellar fat pad, excluding muscle) were isolated using a dissecting microscope. Posterior synovial tissue could not be readily isolated without iatrogenic damage to cartilage and contamination with the closely associated biceps-femoris, semi-tendinosus, semi-membranosus and gastrocnemius muscles (Supplementary Figure 1). RNA was extracted using TRIZOL as previously described^31^ and isolated using the miRCURY RNA Tissue Isolation Kit (Exiqon) including an on-column DNase digestion. RNA samples were quantified by Xpose (Trinean) and checked for RNA integrity by capillary electrophoresis with a Bioanalyzer 2100 (Agilent) prior to expression profiling.

### miRNA and mRNA microarray expression profiling

Expression profiling was performed at the Ramaciotti Centre for Genomics (UNSW, Sydney, Australia). miRNA and mRNA expression profiling was performed using SurePrint Mouse miRNA Microarray Technology, release 21 (G4859C, Agilent Technologies) and SurePrint Mouse mRNA Expression V2 Microarray Technology (G4852B, Agilent Technologies), respectively. Briefly, 100 ng of total RNA was labelled and hybridized using either the miRNA Microarray System with miRNA Complete and Hyb Labeling kit version 3.0 or the Low Input Quick Amp WT Labeling Kit (Agilent Technologies). Randomized placement of samples on the arrays was performed. The arrays were scanned on a G2565CA microarray scanner and the features were extracted using Agilent Feature Extraction 12.0.07 software.

### Quantitative polymerase chain reaction (qPCR)

qPCR validation was carried out on a LightCycler 480 Instrument (Roche) using the miRCURY LNA microRNA PCR system (Exiqon) for miRNA expression. All qPCR reactions were performed in duplicate. U6 snRNA and 5s rRNA were used as internal reference controls. qPCR validation of mRNA expression was performed using the Transcriptor High Fidelity cDNA Synthesis Kit (Roche Diagnostics) and SYBR qPCR Master Mix (Agilent Technologies) and gene-specific primers (Supplementary Table 1). All qPCR reactions were performed in duplicate and normalized to internal housekeeping genes, *Atp5b* and *Rpl10*.

### Data analysis

Comparison of histopathology scores between groups was done using nonparametric ranked Kruskal-Wallis analysis, and when significant (P < 0.05) Mann-Whitney U-test (for unpaired data) for between group comparisons (StatSE, Stata-corporation), with P < 0.016 considered significant after Benjamini-Hochberg correction for multiple comparisons.

The array data was processed in statistical language R, using the limma package (limma_3.20.9) that utilizes linear models to assess differential expression in the context of multifactor designed experiments^46^. Data was background corrected (Normexp) and normalized using cyclic loess which we previously demonstrated as the optimized approach for normalizing miRNA microarray expression data^47^. Only probes with 10% greater signal than the negative controls in at least 5 samples were maintained for differential expression analysis. Probes were summarized at the gene level and data adjusted for multiple testing using the Benjamini-Hochberg method to control for false discovery rate. The data has been submitted to the GEO data repository (https://www.ncbi.nlm.nih.gov/geo/; accession number GSE99738). Differentially expressed miRNAs (394) and mRNAs (top 1000) with adj.p.val < 0.05 between time-points were uploaded into Ingenuity Pathway Analysis (Ingenuity Systems) to perform functional analysis of the two datasets.

qPCR data was quantitated using the comparative Ct method^48^ and results were analyzed by Mann-Whitney U-test to evaluate statistical differences between DMM/sham and post-operative times (GraphPad Prism, version 6.02).

## Electronic supplementary material

Supplementary Data
