# Supplementary material for: Comprehensive Expression Analysis of microRNAs and mRNAs in Synovial Tissue from a Mouse Model of Early Post-Traumatic Osteoarthritis
Source: Sci Rep. 2017 Dec 18;7:17701. doi: 10.1038/s41598-017-17545-1 (PMC5735155; doi:10.1038/s41598-017-17545-1)
Supplement: Supplementary file 1 — Supplementary Data [file 41598_2017_17545_MOESM1_ESM.pdf]

SUPPLEMENTARY DATA

Comprehensive Expression Analysis of microRNAs and mRNAs in Synovial Tissue from a Mouse Model of Early Post-Traumatic Osteoarthritis

Louise H.W. Kung, Varshini Ravi, Lynn Rowley, Katrina M. Bell, Christopher B. Little and John F. Bateman

Supplementary Figures 1-2

Supplementary Tables 1-7

Supplementary Figs 1-2

Comprehensive Expression Analysis of microRNAs and mRNAs in Osteoarthritic Synovial Tissue from a Mouse Model of Early Post-Traumatic OA

Louise H.W. Kung et al

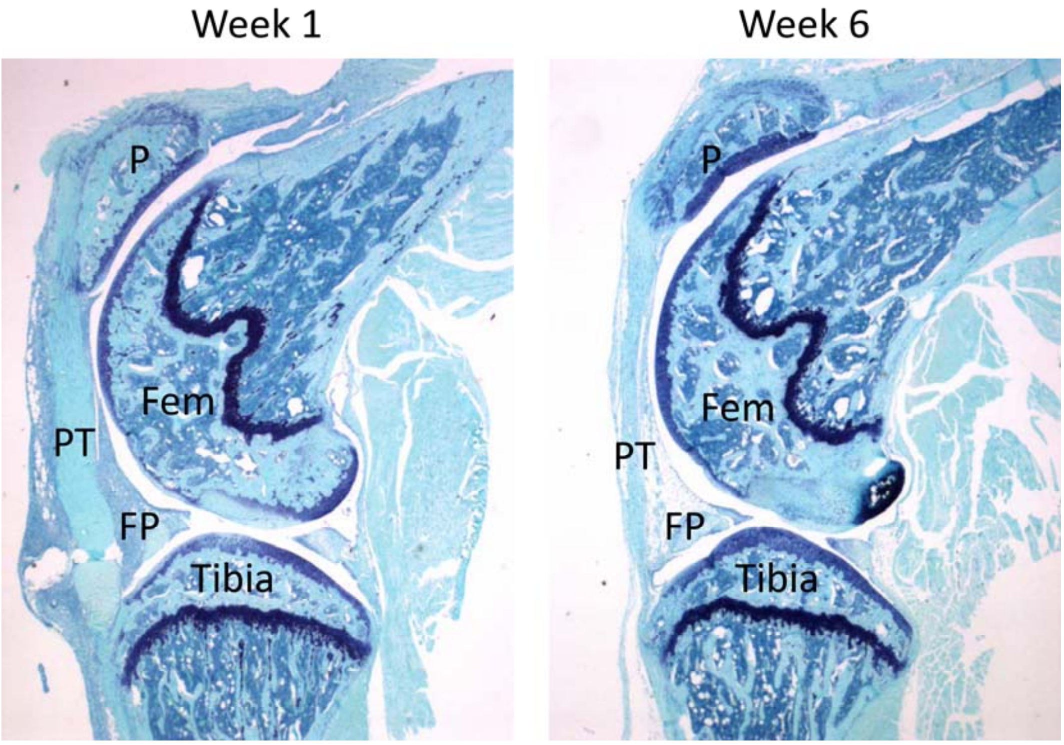

Supplementary Figure 1. Representative images of toluidine blue/fast green stained mid-sagittal sections of mouse knee joints 1 and 6 weeks post DMM surgery, showing thickening and inflammatory cell infiltration in the synovium (particularly anterior) that resolves with time. There is limited tissue overlying the anterior synovium and fat pad allowing for easy isolation, as opposed to the posterior synovium with closely associated overlying muscle. The femur (Fem), Tibia, patella (P), paella tendon (PT) and infra-patella fat pad (FP) are identified.

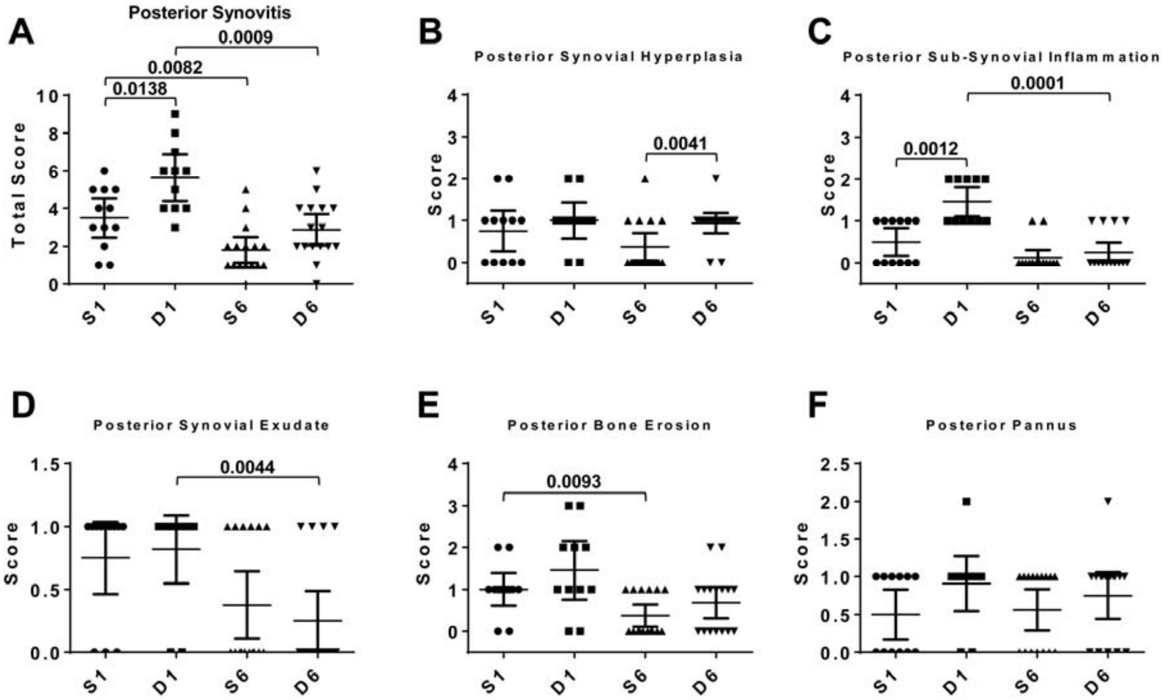

Supplementary Figure 2. Histologic scoring examining synovitis parameters in the posterior region of mouse knee joints: **(A)** synovitis, **(B)** synovial hyperplasia, **(C)** sub-synovial inflammation, **(D)** synovial exudate, **(E)** bone erosion and **(F)** pannus at 1 and 6 weeks post DMM and sham surgeries. n = 12 (S1), n = 11 (D1), n = 16 (S6), n = 16 (D6). Scatter plots display values for each mouse and mean (horizontal bar) ± 95% confidence intervals. Significant differences between groups connected by lines with exact p-values for each comparison indicated above the line. S = Sham; D = DMM.

Supplementary Table 1. qPCR primer sequences for quantification of gene expression

**Gene Name**	**Forward 5'-3'**	**Reverse 5'-3**
Prg4	TGCCATCAACTACTCGGTGT	CAACAGCACGCTCAAAGC
Prelp	ATACAGCGTCCGCATTCC	TGTGCTGGGTGTTTTCCATA
Col6a1	TGGATCCAGCCATCTCGT	GGTGTGAAGACACCACGGATA
Fn1	CAATTCAGATTGCCTAGAAATACCT	CAACTGCATACAAAGTGTCTTCAA
Mmp2	CCTAGGGGATGCTTGGATATT	AAGAACCAGAAGAGTGGAGCAC
Ccl9	GCTGGGTCTGCCCACTAAGA	AATTTCAAGCCCTTGCTGTGC
Ccl7	AGGATCTCTGCCACGCTTCT	CTCCTCGACCCACTTCTGATG
Cxcl1	CCGAAGTCATAGCCACACTCA	TTCTCCGTTACTTGGGGACAC
Cxcl5	TGCCCTACGGTGGAAGTCAT	TGCGAGTGCATTCCGCTTA
Ccl2	CTGCTGTTCACAGTTGCCG	CATTCCTTCTTGGGGTCAGC

Supplementary Table 2: miRNA expression profiles between 1 week post DMM and sham synovium samples

miRNA	miRBase Accession No.	Log_2FC	adj.p.val
mmu-let-7a-5p	MIMAT0000521	-0.106767687	0.990320465
mmu-let-7b-3p	MIMAT0004621	-0.588746068	0.990320465
mmu-let-7b-5p	MIMAT0000522	-0.049060523	0.990320465
mmu-let-7c-5p	MIMAT0000523	-0.075069106	0.990320465
mmu-let-7d-5p	MIMAT0000383	-0.071817503	0.990320465
mmu-let-7e-5p	MIMAT0000524	-0.039707786	0.990320465
mmu-let-7f-5p	MIMAT0000525	-0.122889077	0.990320465
mmu-let-7g-5p	MIMAT0000121	-0.112613319	0.990320465
mmu-let-7i-5p	MIMAT0000122	-0.069415607	0.990320465
mmu-let-7k	MIMAT0025580	-0.10123742	0.990320465
mmu-miR-100-5p	MIMAT0000655	-0.083770034	0.990320465
mmu-miR-101a-3p	MIMAT0000133	-0.187491009	0.990320465
mmu-miR-101c	MIMAT0019349	-0.210270914	0.990320465
mmu-miR-103-3p	MIMAT0000546	-0.119013941	0.990320465
mmu-miR-106b-5p	MIMAT0000386	0.003492179	0.990320465
mmu-miR-107-3p	MIMAT0000647	-0.126564994	0.990320465
mmu-miR-10a-5p	MIMAT0000648	-0.105021663	0.990320465
mmu-miR-10b-5p	MIMAT0000208	-0.119494979	0.990320465
mmu-miR-1187	MIMAT0005837	-0.018159138	0.990320465
mmu-miR-1188-5p	MIMAT0005843	0.013230338	0.990320465
mmu-miR-1198-5p	MIMAT0005859	0.376712989	0.990320465
mmu-miR-1224-5p	MIMAT0005460	-0.148754426	0.990320465
mmu-miR-1231-5p	MIMAT0022357	0.012906231	0.990320465
mmu-miR-1249-3p	MIMAT0010560	0.12623184	0.990320465
mmu-miR-125a-3p	MIMAT0004528	0.122641214	0.990320465
mmu-miR-125a-5p	MIMAT0000135	0.09716303	0.990320465
mmu-miR-125b-5p	MIMAT0000136	-0.02631713	0.990320465
mmu-miR-126a-3p	MIMAT0000138	-0.156262613	0.990320465
mmu-miR-126a-5p	MIMAT0000137	-0.145351264	0.990320465
mmu-miR-127-3p	MIMAT0000139	-0.035409511	0.990320465
mmu-miR-128-3p	MIMAT0000140	0.026205414	0.990320465
mmu-miR-129-5p	MIMAT0000209	-0.037809377	0.990320465
mmu-miR-129b-5p	MIMAT0029862	0.046808612	0.990320465
mmu-miR-1306-3p	MIMAT0009411	-0.10319135	0.990320465
mmu-miR-130a-3p	MIMAT0000141	-0.041237595	0.990320465
mmu-miR-130b-3p	MIMAT0000387	0.097440861	0.990320465
mmu-miR-132-3p	MIMAT0000144	0.107054872	0.990320465
mmu-miR-133a-3p	MIMAT0000145	0.426568517	0.990320465
mmu-miR-133a-5p	MIMAT0003473	0.154948756	0.990320465
mmu-miR-133b-3p	MIMAT0000769	0.399194691	0.990320465
mmu-miR-134-5p	MIMAT0000146	0.087777782	0.990320465
mmu-miR-135a-1-3p	MIMAT0004531	-0.192351427	0.990320465
mmu-miR-136-5p	MIMAT0000148	-0.071526555	0.990320465
mmu-miR-139-3p	MIMAT0004662	-0.010909586	0.990320465
mmu-miR-139-5p	MIMAT0000656	0.001482578	0.990320465
mmu-miR-140-3p	MIMAT0000152	0.309687222	0.990320465
mmu-miR-140-5p	MIMAT0000151	0.223074155	0.990320465

mmu-miR-142a-3p	MIMAT0000155	-0.133328712	0.990320465
mmu-miR-142a-5p	MIMAT0000154	-0.094765713	0.990320465
mmu-miR-143-3p	MIMAT0000247	-0.080053555	0.990320465
mmu-miR-144-3p	MIMAT0000156	0.069014423	0.990320465
mmu-miR-144-5p	MIMAT0016988	0.35189933	0.990320465
mmu-miR-145a-5p	MIMAT0000157	-0.039476361	0.990320465
mmu-miR-146a-5p	MIMAT0000158	-0.246623851	0.990320465
mmu-miR-146b-5p	MIMAT0003475	0.020803197	0.990320465
mmu-miR-148a-3p	MIMAT0000516	0.047592103	0.990320465
mmu-miR-148b-3p	MIMAT0000580	0.004571481	0.990320465
mmu-miR-149-3p	MIMAT0016990	0.010976532	0.990320465
mmu-miR-149-5p	MIMAT0000159	0.126054209	0.990320465
mmu-miR-150-3p	MIMAT0004535	-0.060433935	0.990320465
mmu-miR-150-5p	MIMAT0000160	-0.043265866	0.990320465
mmu-miR-151-5p	MIMAT0004536	-0.052439443	0.990320465
mmu-miR-152-3p	MIMAT0000162	0.066889392	0.990320465
mmu-miR-154-3p	MIMAT0004537	-0.108088236	0.990320465
mmu-miR-154-5p	MIMAT0000164	-0.083668483	0.990320465
mmu-miR-155-5p	MIMAT0000165	-0.024907937	0.990320465
mmu-miR-15a-5p	MIMAT0000526	-0.125768839	0.990320465
mmu-miR-15b-5p	MIMAT0000124	-0.007718421	0.990320465
mmu-miR-16-5p	MIMAT0000527	-0.072291901	0.990320465
mmu-miR-17-3p	MIMAT0000650	0.033507181	0.990320465
mmu-miR-17-5p	MIMAT0000649	0.09170329	0.990320465
mmu-miR-181a-1-3p	MIMAT0000660	0.010020678	0.990320465
mmu-miR-181a-5p	MIMAT0000210	0.071461791	0.990320465
mmu-miR-181b-5p	MIMAT0000673	0.077075426	0.990320465
mmu-miR-181c-5p	MIMAT0000674	-0.050380215	0.990320465
mmu-miR-181d-5p	MIMAT0004324	0.076638101	0.990320465
mmu-miR-1839-3p	MIMAT0009457	0.039148077	0.990320465
mmu-miR-1839-5p	MIMAT0009456	-0.122168127	0.990320465
mmu-miR-185-5p	MIMAT0000214	0.004084322	0.990320465
mmu-miR-188-5p	MIMAT0000217	-0.004876513	0.990320465
mmu-miR-1892	MIMAT0007871	-0.163957043	0.990320465
mmu-miR-1894-3p	MIMAT0007878	-0.01846884	0.990320465
mmu-miR-1895	MIMAT0007867	0.014274948	0.990320465
mmu-miR-1896	MIMAT0007873	-0.056334742	0.990320465
mmu-miR-1897-3p	MIMAT0007865	-0.011865124	0.990320465
mmu-miR-1897-5p	MIMAT0007864	0.301541915	0.990320465
mmu-miR-18a-5p	MIMAT0000528	0.021265091	0.990320465
mmu-miR-1901	MIMAT0007880	0.140230758	0.990320465
mmu-miR-1904	MIMAT0007874	-0.022146204	0.990320465
mmu-miR-1906	MIMAT0007872	0.106562413	0.990320465
mmu-miR-1907	MIMAT0007876	0.082496437	0.990320465
mmu-miR-190b-5p	MIMAT0004852	-0.004688777	0.990320465
mmu-miR-191-3p	MIMAT0004542	0.003403778	0.990320465
mmu-miR-192-5p	MIMAT0000517	0.02512725	0.990320465
mmu-miR-1927	MIMAT0009390	0.068161674	0.990320465
mmu-miR-1930-3p	MIMAT0017340	-0.046143266	0.990320465
mmu-miR-1931	MIMAT0009394	0.172693359	0.990320465
mmu-miR-1934-3p	MIMAT0017341	-0.022495178	0.990320465

mmu-miR-193a-3p	MIMAT0000223	-0.132572616	0.990320465
mmu-miR-193b-3p	MIMAT0004859	0.006853199	0.990320465
mmu-miR-193b-5p	MIMAT0017271	0.043596915	0.990320465
mmu-miR-194-5p	MIMAT0000224	-0.071692921	0.990320465
mmu-miR-1941-5p	MIMAT0009405	-0.328860083	0.990320465
mmu-miR-1945	MIMAT0009410	0.163274142	0.990320465
mmu-miR-1949	MIMAT0009416	0.090827783	0.990320465
mmu-miR-1955-3p	MIMAT0017348	0.079714936	0.990320465
mmu-miR-1957a	MIMAT0009430	0.145808276	0.990320465
mmu-miR-195a-3p	MIMAT0017000	-0.006009614	0.990320465
mmu-miR-195a-5p	MIMAT0000225	-0.073458328	0.990320465
mmu-miR-1960	MIMAT0009433	-0.143306368	0.990320465
mmu-miR-1966-5p	MIMAT0009439	-0.133565061	0.990320465
mmu-miR-1967	MIMAT0009440	-0.061531454	0.990320465
mmu-miR-1968-5p	MIMAT0009441	0.01899498	0.990320465
mmu-miR-196a-5p	MIMAT0000518	0.008689419	0.990320465
mmu-miR-196b-5p	MIMAT0001081	-0.09016646	0.990320465
mmu-miR-1971	MIMAT0009446	-0.114743904	0.990320465
mmu-miR-1982-5p	MIMAT0009459	0.109180505	0.990320465
mmu-miR-199a-3p	MIMAT0000230	-0.020945909	0.990320465
mmu-miR-199a-5p	MIMAT0000229	0.131926456	0.990320465
mmu-miR-199b-5p	MIMAT0000672	-0.010393835	0.990320465
mmu-miR-19a-3p	MIMAT0000651	-0.005311319	0.990320465
mmu-miR-19b-3p	MIMAT0000513	-0.005453434	0.990320465
mmu-miR-1a-3p	MIMAT0000123	0.401273366	0.990320465
mmu-miR-202-3p	MIMAT0000235	0.07218863	0.990320465
mmu-miR-203-3p	MIMAT0000236	-0.122876716	0.990320465
mmu-miR-204-5p	MIMAT0000237	-0.182488181	0.990320465
mmu-miR-206-3p	MIMAT0000239	0.222359285	0.990320465
mmu-miR-208a-5p	MIMAT0017014	-0.092435641	0.990320465
mmu-miR-20a-5p	MIMAT0000529	0.046739891	0.990320465
mmu-miR-20b-5p	MIMAT0003187	0.047721645	0.990320465
mmu-miR-210-3p	MIMAT0000658	0.139810112	0.990320465
mmu-miR-211-3p	MIMAT0017059	0.300751752	0.990320465
mmu-miR-212-3p	MIMAT0000659	0.082116937	0.990320465
mmu-miR-2137	MIMAT0011213	0.006944314	0.990320465
mmu-miR-214-3p	MIMAT0000661	0.116365278	0.990320465
mmu-miR-214-5p	MIMAT0004664	0.153804709	0.990320465
mmu-miR-218-5p	MIMAT0000663	-0.050843047	0.990320465
mmu-miR-21a-3p	MIMAT0004628	0.096726016	0.990320465
mmu-miR-21a-5p	MIMAT0000530	-0.116332564	0.990320465
mmu-miR-22-3p	MIMAT0000531	-0.084613981	0.990320465
mmu-miR-22-5p	MIMAT0004629	-0.049406158	0.990320465
mmu-miR-221-3p	MIMAT0000669	-0.099700508	0.990320465
mmu-miR-222-3p	MIMAT0000670	-0.099582792	0.990320465
mmu-miR-223-3p	MIMAT0000665	0.036152785	0.990320465
mmu-miR-224-5p	MIMAT0000671	0.056357498	0.990320465
mmu-miR-23a-3p	MIMAT0000532	-0.115053581	0.990320465
mmu-miR-23a-5p	MIMAT0017019	-0.0929987	0.990320465
mmu-miR-23b-3p	MIMAT0000125	-0.117101152	0.990320465
mmu-miR-24-2-5p	MIMAT0005440	-0.027015786	0.990320465

mmu-miR-24-3p	MIMAT0000219	-0.095044713	0.990320465
mmu-miR-25-3p	MIMAT0000652	0.034888106	0.990320465
mmu-miR-26a-5p	MIMAT0000533	-0.108260048	0.990320465
mmu-miR-26b-5p	MIMAT0000534	-0.149345419	0.990320465
mmu-miR-27a-3p	MIMAT0000537	-0.077515979	0.990320465
mmu-miR-27b-3p	MIMAT0000126	-0.031619585	0.990320465
mmu-miR-2861	MIMAT0013803	0.117122201	0.990320465
mmu-miR-28a-5p	MIMAT0000653	0.033999459	0.990320465
mmu-miR-28c	MIMAT0019339	-0.020919987	0.990320465
mmu-miR-290a-5p	MIMAT0000366	0.222946335	0.990320465
mmu-miR-292a-5p	MIMAT0000369	0.180339587	0.990320465
mmu-miR-295-5p	MIMAT0004575	-0.002663055	0.990320465
mmu-miR-296-5p	MIMAT0000374	0.141312662	0.990320465
mmu-miR-299a-3p	MIMAT0004577	0.177946527	0.990320465
mmu-miR-299b-5p	MIMAT0022836	0.007965234	0.990320465
mmu-miR-29a-3p	MIMAT0000535	-0.162941326	0.990320465
mmu-miR-29b-1-5p	MIMAT0004523	-0.011906021	0.990320465
mmu-miR-29b-3p	MIMAT0000127	-0.113207087	0.990320465
mmu-miR-29c-3p	MIMAT0000536	-0.191611353	0.990320465
mmu-miR-29c-5p	MIMAT0004632	-0.018941697	0.990320465
mmu-miR-300-3p	MIMAT0000378	0.040485809	0.990320465
mmu-miR-301a-3p	MIMAT0000379	-0.154325572	0.990320465
mmu-miR-302c-5p	MIMAT0003375	0.056807873	0.990320465
mmu-miR-3058-3p	MIMAT0014814	0.051841633	0.990320465
mmu-miR-3058-5p	MIMAT0014813	0.098888589	0.990320465
mmu-miR-3059-3p	MIMAT0014812	-0.026180261	0.990320465
mmu-miR-3067-3p	MIMAT0014841	0.031680479	0.990320465
mmu-miR-3069-3p	MIMAT0014845	-0.005658676	0.990320465
mmu-miR-3070-2-3p	MIMAT0014849	0.044281854	0.990320465
mmu-miR-3072-5p	MIMAT0014852	-0.027552561	0.990320465
mmu-miR-3075-5p	MIMAT0014858	0.005046016	0.990320465
mmu-miR-3077-5p	MIMAT0014862	0.092430968	0.990320465
mmu-miR-3081-5p	MIMAT0014870	-0.085543379	0.990320465
mmu-miR-3082-5p	MIMAT0014872	-0.031252484	0.990320465
mmu-miR-3085-3p	MIMAT0014879	0.08698356	0.990320465
mmu-miR-3085-5p	MIMAT0014878	0.084750891	0.990320465
mmu-miR-3092-3p	MIMAT0014906	0.211988385	0.990320465
mmu-miR-3093-3p	MIMAT0014908	0.034145744	0.990320465
mmu-miR-3095-3p	MIMAT0014912	0.130793406	0.990320465
mmu-miR-3098-5p	MIMAT0014917	-0.075948259	0.990320465
mmu-miR-3099-3p	MIMAT0014816	0.126555081	0.990320465
mmu-miR-30a-3p	MIMAT0000129	-0.042181922	0.990320465
mmu-miR-30a-5p	MIMAT0000128	-0.204950599	0.990320465
mmu-miR-30b-3p	MIMAT0004524	-0.076820071	0.990320465
mmu-miR-30b-5p	MIMAT0000130	-0.09831436	0.990320465
mmu-miR-30c-1-3p	MIMAT0004616	-0.033120603	0.990320465
mmu-miR-30c-2-3p	MIMAT0005438	-0.029258125	0.990320465
mmu-miR-30c-5p	MIMAT0000514	-0.154400893	0.990320465
mmu-miR-30d-5p	MIMAT0000515	-0.024423337	0.990320465
mmu-miR-30e-3p	MIMAT0000249	-0.054870254	0.990320465
mmu-miR-30e-5p	MIMAT0000248	-0.100513659	0.990320465

mmu-miR-31-5p	MIMAT0000538	0.237193955	0.990320465
mmu-miR-3101-3p	MIMAT0014922	-0.055862909	0.990320465
mmu-miR-3102-3p	MIMAT0014936	0.001834461	0.990320465
mmu-miR-3102-5p	MIMAT0014933	0.058145741	0.990320465
mmu-miR-3102-5p.2-5p	MIMAT0014934	0.063789811	0.990320465
mmu-miR-3110-3p	MIMAT0014952	0.012178466	0.990320465
mmu-miR-3154	MIMAT0035714	0.117010794	0.990320465
mmu-miR-320-3p	MIMAT0000666	0.085135183	0.990320465
mmu-miR-322-5p	MIMAT0000548	-0.016533366	0.990320465
mmu-miR-324-3p	MIMAT0000556	0.056540141	0.990320465
mmu-miR-324-5p	MIMAT0000555	-0.033253101	0.990320465
mmu-miR-327	MIMAT0004867	-0.02115035	0.990320465
mmu-miR-328-5p	MIMAT0017030	0.198665701	0.990320465
mmu-miR-329-3p	MIMAT0000567	-0.035852994	0.990320465
mmu-miR-331-3p	MIMAT0000571	0.013355028	0.990320465
mmu-miR-335-5p	MIMAT0000766	0.187032182	0.990320465
mmu-miR-337-3p	MIMAT0000578	-0.017338165	0.990320465
mmu-miR-337-5p	MIMAT0004644	-0.020379036	0.990320465
mmu-miR-338-3p	MIMAT0000582	-0.125273026	0.990320465
mmu-miR-338-5p	MIMAT0004647	0.003235503	0.990320465
mmu-miR-340-5p	MIMAT0004651	-0.080196783	0.990320465
mmu-miR-341-3p	MIMAT0000588	-0.111854106	0.990320465
mmu-miR-342-3p	MIMAT0000590	-0.127254408	0.990320465
mmu-miR-344i	MIMAT0022503	0.377311682	0.990320465
mmu-miR-345-5p	MIMAT0000595	0.062050891	0.990320465
mmu-miR-3470a	MIMAT0015640	0.121425195	0.990320465
mmu-miR-3472	MIMAT0015643	0.015069145	0.990320465
mmu-miR-3473a	MIMAT0015645	0.233048668	0.990320465
mmu-miR-3473b	MIMAT0020367	0.144302301	0.990320465
mmu-miR-3473e	MIMAT0025587	0.216068528	0.990320465
mmu-miR-3473f	MIMAT0031390	0.174182946	0.990320465
mmu-miR-3473g	MIMAT0031427	0.087083792	0.990320465
mmu-miR-3474	MIMAT0015646	-0.068907245	0.990320465
mmu-miR-34a-5p	MIMAT0000542	-0.078665085	0.990320465
mmu-miR-34b-5p	MIMAT0000382	-0.026993239	0.990320465
mmu-miR-34c-5p	MIMAT0000381	0.015848709	0.990320465
mmu-miR-350-3p	MIMAT0000605	-0.07835353	0.990320465
mmu-miR-3535	MIMAT0031410	-0.075809928	0.990320465
mmu-miR-3544-3p	MIMAT0022354	0.05418535	0.990320465
mmu-miR-3547-5p	MIMAT0027832	0.181882108	0.990320465
mmu-miR-3572-5p	MIMAT0022986	0.070112735	0.990320465
mmu-miR-361-5p	MIMAT0000704	0.003067225	0.990320465
mmu-miR-362-3p	MIMAT0004684	-0.101045837	0.990320465
mmu-miR-362-5p	MIMAT0000706	0.050955649	0.990320465
mmu-miR-3620-3p	MIMAT0029879	0.082611356	0.990320465
mmu-miR-3620-5p	MIMAT0029878	0.116962482	0.990320465
mmu-miR-365-1-5p	MIMAT0017077	-0.087864698	0.990320465
mmu-miR-365-3p	MIMAT0000711	-0.138560694	0.990320465
mmu-miR-370-3p	MIMAT0001095	0.071721975	0.990320465
mmu-miR-374c-5p	MIMAT0014953	-0.0067475	0.990320465
mmu-miR-376a-3p	MIMAT0000740	-0.047903155	0.990320465

mmu-miR-376b-3p	MIMAT0001092	-0.072188764	0.990320465
mmu-miR-376c-3p	MIMAT0003183	-0.042698745	0.990320465
mmu-miR-377-3p	MIMAT0000741	-0.078221777	0.990320465
mmu-miR-378a-3p	MIMAT0003151	0.110481228	0.990320465
mmu-miR-378a-5p	MIMAT0000742	0.043593347	0.990320465
mmu-miR-378b	MIMAT0019348	0.049288101	0.990320465
mmu-miR-378c	MIMAT0025138	0.022026623	0.990320465
mmu-miR-378d	MIMAT0025167	0.038431053	0.990320465
mmu-miR-379-5p	MIMAT0000743	-0.029195866	0.990320465
mmu-miR-381-3p	MIMAT0000746	0.052458633	0.990320465
mmu-miR-382-5p	MIMAT0000747	-0.006650605	0.990320465
mmu-miR-3960	MIMAT0019336	0.186441693	0.990320465
mmu-miR-3963	MIMAT0019341	0.071521014	0.990320465
mmu-miR-3968	MIMAT0019352	-0.066028709	0.990320465
mmu-miR-409-3p	MIMAT0001090	-0.008718181	0.990320465
mmu-miR-410-3p	MIMAT0001091	-0.045289073	0.990320465
mmu-miR-411-3p	MIMAT0001093	0.006333193	0.990320465
mmu-miR-411-5p	MIMAT0004747	-0.035906419	0.990320465
mmu-miR-423-5p	MIMAT0004825	0.068792075	0.990320465
mmu-miR-425-5p	MIMAT0004750	-0.043294392	0.990320465
mmu-miR-431-5p	MIMAT0001418	-0.127943469	0.990320465
mmu-miR-432	MIMAT0012771	-0.113983678	0.990320465
mmu-miR-434-3p	MIMAT0001422	0.003593447	0.990320465
mmu-miR-434-5p	MIMAT0001421	-0.055660506	0.990320465
mmu-miR-450a-2-3p	MIMAT0004789	-0.169861501	0.990320465
mmu-miR-451a	MIMAT0001632	0.178733866	0.990320465
mmu-miR-452-5p	MIMAT0001637	-0.023765984	0.990320465
mmu-miR-455-3p	MIMAT0003742	0.10583296	0.990320465
mmu-miR-455-5p	MIMAT0003485	0.018392354	0.990320465
mmu-miR-466f-3p	MIMAT0004882	-0.064447041	0.990320465
mmu-miR-466g	MIMAT0004883	-0.119608403	0.990320465
mmu-miR-466h-3p	MIMAT0017274	-0.116182288	0.990320465
mmu-miR-466i-3p	MIMAT0005834	-0.113686933	0.990320465
mmu-miR-466i-5p	MIMAT0017325	-0.055606981	0.990320465
mmu-miR-466m-3p	MIMAT0014883	-0.092354246	0.990320465
mmu-miR-466q	MIMAT0020631	-0.073850902	0.990320465
mmu-miR-467a-3p	MIMAT0002108	-0.007627727	0.990320465
mmu-miR-467f	MIMAT0005846	-0.112011522	0.990320465
mmu-miR-468-3p	MIMAT0002109	0.019321055	0.990320465
mmu-miR-470-5p	MIMAT0002111	-0.031786238	0.990320465
mmu-miR-483-3p	MIMAT0003120	-0.034556706	0.990320465
mmu-miR-483-5p	MIMAT0004782	0.050992197	0.990320465
mmu-miR-484	MIMAT0003127	0.624245313	0.990320465
mmu-miR-486a-3p	MIMAT0017206	-0.041479153	0.990320465
mmu-miR-486a-5p	MIMAT0003130	0.218676972	0.990320465
mmu-miR-487b-3p	MIMAT0003184	0.041330913	0.990320465
mmu-miR-490-5p	MIMAT0017261	0.009472241	0.990320465
mmu-miR-494-3p	MIMAT0003182	-0.302520259	0.990320465
mmu-miR-495-3p	MIMAT0003456	-0.029160705	0.990320465
mmu-miR-497a-5p	MIMAT0003453	-0.009925538	0.990320465
mmu-miR-500-3p	MIMAT0003507	0.038248269	0.990320465

mmu-miR-503-5p	MIMAT0003188	0.175367378	0.990320465
mmu-miR-504-3p	MIMAT0017277	0.029594602	0.990320465
mmu-miR-5099	MIMAT0020606	0.063433349	0.990320465
mmu-miR-5100	MIMAT0020607	0.21798602	0.990320465
mmu-miR-5103	MIMAT0020610	0.089938718	0.990320465
mmu-miR-5104	MIMAT0020611	0.104419359	0.990320465
mmu-miR-5107-5p	MIMAT0020615	-0.001699948	0.990320465
mmu-miR-511-3p	MIMAT0017281	-0.22987953	0.990320465
mmu-miR-5110	MIMAT0020618	-0.131241896	0.990320465
mmu-miR-5112	MIMAT0020620	0.049064085	0.990320465
mmu-miR-5113	MIMAT0020621	0.109814265	0.990320465
mmu-miR-5118	MIMAT0020626	0.205819052	0.990320465
mmu-miR-5119	MIMAT0020627	-0.022113966	0.990320465
mmu-miR-5121	MIMAT0020629	-0.261343072	0.990320465
mmu-miR-5122	MIMAT0020630	-0.042501682	0.990320465
mmu-miR-5126	MIMAT0020637	0.103125036	0.990320465
mmu-miR-5128	MIMAT0020639	0.027521049	0.990320465
mmu-miR-5130	MIMAT0020641	0.004247367	0.990320465
mmu-miR-5131	MIMAT0020642	-0.016239397	0.990320465
mmu-miR-5132-5p	MIMAT0020643	-0.052174325	0.990320465
mmu-miR-5135	MIMAT0020646	0.029157902	0.990320465
mmu-miR-532-3p	MIMAT0004781	0.046700538	0.990320465
mmu-miR-532-5p	MIMAT0002889	-0.038263428	0.990320465
mmu-miR-541-5p	MIMAT0003170	0.064907314	0.990320465
mmu-miR-546	MIMAT0003166	0.062096746	0.990320465
mmu-miR-551b-5p	MIMAT0017236	0.127072387	0.990320465
mmu-miR-5620-3p	MIMAT0022368	0.077107481	0.990320465
mmu-miR-5622-3p	MIMAT0022372	-0.175595123	0.990320465
mmu-miR-574-3p	MIMAT0004894	-0.079998558	0.990320465
mmu-miR-574-5p	MIMAT0004893	-0.007765831	0.990320465
mmu-miR-615-3p	MIMAT0003783	-0.030379735	0.990320465
mmu-miR-6236	MIMAT0024857	-0.063581992	0.990320465
mmu-miR-6238	MIMAT0024859	-0.14295151	0.990320465
mmu-miR-6349	MIMAT0025092	-0.027333188	0.990320465
mmu-miR-6351	MIMAT0025094	-0.053919707	0.990320465
mmu-miR-6354	MIMAT0025097	-0.293276882	0.990320465
mmu-miR-6360	MIMAT0025103	-0.056093739	0.990320465
mmu-miR-6366	MIMAT0025110	-0.038744296	0.990320465
mmu-miR-6368	MIMAT0025112	0.070569382	0.990320465
mmu-miR-6370	MIMAT0025114	0.067539227	0.990320465
mmu-miR-6378	MIMAT0025124	-0.307093527	0.990320465
mmu-miR-6385	MIMAT0025131	-0.134893795	0.990320465
mmu-miR-6386	MIMAT0025133	-0.073640241	0.990320465
mmu-miR-6388	MIMAT0025135	0.00929403	0.990320465
mmu-miR-6391	MIMAT0025140	-0.082198579	0.990320465
mmu-miR-6392-3p	MIMAT0025142	0.173841789	0.990320465
mmu-miR-6394	MIMAT0025144	0.11788382	0.990320465
mmu-miR-6401	MIMAT0025153	-0.047828502	0.990320465
mmu-miR-6402	MIMAT0025154	0.181203726	0.990320465
mmu-miR-6405	MIMAT0025157	0.029428335	0.990320465
mmu-miR-6407	MIMAT0025160	-0.088422111	0.990320465

mmu-miR-6412	MIMAT0025165	0.081417249	0.990320465
mmu-miR-6418-5p	MIMAT0025173	-0.051475152	0.990320465
mmu-miR-652-3p	MIMAT0003711	0.008771525	0.990320465
mmu-miR-652-5p	MIMAT0017260	0.019658936	0.990320465
mmu-miR-6538	MIMAT0025583	0.084107076	0.990320465
mmu-miR-664-5p	MIMAT0017353	-0.144170454	0.990320465
mmu-miR-669c-3p	MIMAT0017253	-0.019292118	0.990320465
mmu-miR-669f-3p	MIMAT0005839	-0.020718689	0.990320465
mmu-miR-669h-3p	MIMAT0005842	-0.075285854	0.990320465
mmu-miR-669l-5p	MIMAT0009418	-0.057430298	0.990320465
mmu-miR-669n	MIMAT0009427	-0.062272391	0.990320465
mmu-miR-669p-3p	MIMAT0014890	-0.110664771	0.990320465
mmu-miR-671-5p	MIMAT0003731	0.066307816	0.990320465
mmu-miR-674-3p	MIMAT0003741	0.012312578	0.990320465
mmu-miR-674-5p	MIMAT0003740	0.024610574	0.990320465
mmu-miR-676-3p	MIMAT0003782	0.004261192	0.990320465
mmu-miR-6769b-5p	MIMAT0028040	0.082836677	0.990320465
mmu-miR-677-3p	MIMAT0017246	0.072052765	0.990320465
mmu-miR-678	MIMAT0003452	0.029090899	0.990320465
mmu-miR-680	MIMAT0003457	0.142416858	0.990320465
mmu-miR-681	MIMAT0003458	0.028152104	0.990320465
mmu-miR-682	MIMAT0003459	0.050208279	0.990320465
mmu-miR-6898-5p	MIMAT0027696	0.109239446	0.990320465
mmu-miR-690	MIMAT0003469	0.216263828	0.990320465
mmu-miR-6901-5p	MIMAT0027702	-0.039004729	0.990320465
mmu-miR-6904-5p	MIMAT0027708	0.032170008	0.990320465
mmu-miR-6905-5p	MIMAT0027710	0.003031887	0.990320465
mmu-miR-6906-5p	MIMAT0027712	0.003338428	0.990320465
mmu-miR-6908-5p	MIMAT0027716	0.052426132	0.990320465
mmu-miR-6909-5p	MIMAT0027718	-0.025540938	0.990320465
mmu-miR-691	MIMAT0003470	-0.03084322	0.990320465
mmu-miR-6910-5p	MIMAT0027720	-0.144379366	0.990320465
mmu-miR-6911-5p	MIMAT0027722	-0.052887806	0.990320465
mmu-miR-6912-5p	MIMAT0027724	-0.018934196	0.990320465
mmu-miR-6915-5p	MIMAT0027730	0.041568726	0.990320465
mmu-miR-6918-5p	MIMAT0027736	0.05530526	0.990320465
mmu-miR-6921-5p	MIMAT0027742	-0.043058717	0.990320465
mmu-miR-6922-5p	MIMAT0027744	-0.073227292	0.990320465
mmu-miR-6923-5p	MIMAT0027746	0.011021495	0.990320465
mmu-miR-6926-5p	MIMAT0027752	0.011086537	0.990320465
mmu-miR-6931-5p	MIMAT0027762	0.018425758	0.990320465
mmu-miR-6934-5p	MIMAT0027768	-0.008964044	0.990320465
mmu-miR-6937-5p	MIMAT0027774	0.029681718	0.990320465
mmu-miR-6939-5p	MIMAT0027778	-0.129282915	0.990320465
mmu-miR-6944-5p	MIMAT0027788	-0.023067145	0.990320465
mmu-miR-6946-5p	MIMAT0027792	0.016880406	0.990320465
mmu-miR-6952-5p	MIMAT0027804	0.029282605	0.990320465
mmu-miR-6954-5p	MIMAT0027808	0.074749005	0.990320465
mmu-miR-6955-5p	MIMAT0027810	-0.063183633	0.990320465
mmu-miR-6956-5p	MIMAT0027812	0.024429802	0.990320465
mmu-miR-6957-5p	MIMAT0027814	-0.057144237	0.990320465

mmu-miR-6959-5p	MIMAT0027818	-0.024825929	0.990320465
mmu-miR-6961-5p	MIMAT0027822	0.038574518	0.990320465
mmu-miR-6963-5p	MIMAT0027826	0.002003504	0.990320465
mmu-miR-6965-5p	MIMAT0027830	0.01465803	0.990320465
mmu-miR-6969-5p	MIMAT0027840	-0.154462857	0.990320465
mmu-miR-697	MIMAT0003487	0.045813834	0.990320465
mmu-miR-6970-5p	MIMAT0027842	-0.001734358	0.990320465
mmu-miR-6971-5p	MIMAT0027844	0.10232774	0.990320465
mmu-miR-6972-5p	MIMAT0027846	-0.006085993	0.990320465
mmu-miR-6973a-5p	MIMAT0027848	-0.067161426	0.990320465
mmu-miR-6973b-5p	MIMAT0027908	0.051886092	0.990320465
mmu-miR-6974-5p	MIMAT0027850	0.075612029	0.990320465
mmu-miR-6975-5p	MIMAT0027852	0.044887401	0.990320465
mmu-miR-6976-3p	MIMAT0027855	0.030064926	0.990320465
mmu-miR-6978-5p	MIMAT0027858	0.106753779	0.990320465
mmu-miR-698-5p	MIMAT0022930	-0.03050125	0.990320465
mmu-miR-6980-5p	MIMAT0027862	0.026043775	0.990320465
mmu-miR-6981-5p	MIMAT0027864	-0.095665431	0.990320465
mmu-miR-6982-5p	MIMAT0027866	-0.045996062	0.990320465
mmu-miR-6984-3p	MIMAT0027871	0.195196655	0.990320465
mmu-miR-6984-5p	MIMAT0027870	0.072196861	0.990320465
mmu-miR-6987-5p	MIMAT0027876	0.032634775	0.990320465
mmu-miR-6988-5p	MIMAT0027878	-0.031045297	0.990320465
mmu-miR-6990-5p	MIMAT0027882	-0.00485014	0.990320465
mmu-miR-6991-5p	MIMAT0027884	-0.001179359	0.990320465
mmu-miR-6995-5p	MIMAT0027892	0.156319069	0.990320465
mmu-miR-6997-5p	MIMAT0027896	0.031238701	0.990320465
mmu-miR-6998-5p	MIMAT0027898	-0.102910468	0.990320465
mmu-miR-6999-5p	MIMAT0027900	-0.026043188	0.990320465
mmu-miR-700-3p	MIMAT0003490	0.038056048	0.990320465
mmu-miR-7002-5p	MIMAT0027906	-0.064663382	0.990320465
mmu-miR-7005-5p	MIMAT0027914	-0.357052624	0.990320465
mmu-miR-7007-5p	MIMAT0027918	0.070947702	0.990320465
mmu-miR-7008-5p	MIMAT0027920	-0.084832843	0.990320465
mmu-miR-7009-5p	MIMAT0027922	0.023048207	0.990320465
mmu-miR-7011-5p	MIMAT0027926	-0.088576797	0.990320465
mmu-miR-7012-5p	MIMAT0027928	0.046826368	0.990320465
mmu-miR-7014-5p	MIMAT0027932	0.047141784	0.990320465
mmu-miR-7015-5p	MIMAT0027934	-0.084144139	0.990320465
mmu-miR-7016-5p	MIMAT0027936	-0.068896827	0.990320465
mmu-miR-7018-5p	MIMAT0027940	0.026642866	0.990320465
mmu-miR-7019-5p	MIMAT0027942	-0.021207785	0.990320465
mmu-miR-702-3p	MIMAT0003492	0.084424066	0.990320465
mmu-miR-7020-5p	MIMAT0027944	-0.217784615	0.990320465
mmu-miR-7022-3p	MIMAT0027949	0.087411077	0.990320465
mmu-miR-7025-5p	MIMAT0027954	0.033888675	0.990320465
mmu-miR-7027-5p	MIMAT0027958	-0.059180676	0.990320465
mmu-miR-7028-5p	MIMAT0027960	0.094382123	0.990320465
mmu-miR-7031-5p	MIMAT0027966	0.207373378	0.990320465
mmu-miR-7032-5p	MIMAT0027968	-0.111016276	0.990320465
mmu-miR-7033-5p	MIMAT0027970	0.03107724	0.990320465

mmu-miR-7034-5p	MIMAT0027972	-0.01364984	0.990320465
mmu-miR-7035-5p	MIMAT0027974	0.03678684	0.990320465
mmu-miR-7036a-5p	MIMAT0027976	0.120331957	0.990320465
mmu-miR-7040-5p	MIMAT0027984	0.083718927	0.990320465
mmu-miR-7042-5p	MIMAT0027988	-0.072766266	0.990320465
mmu-miR-7043-5p	MIMAT0027990	-0.04075427	0.990320465
mmu-miR-7044-5p	MIMAT0027992	-0.050535285	0.990320465
mmu-miR-7045-5p	MIMAT0027994	0.006518493	0.990320465
mmu-miR-7046-5p	MIMAT0027996	0.189522227	0.990320465
mmu-miR-7047-5p	MIMAT0027998	0.079802972	0.990320465
mmu-miR-7048-5p	MIMAT0028000	-0.088877052	0.990320465
mmu-miR-705	MIMAT0003495	0.017039253	0.990320465
mmu-miR-7050-5p	MIMAT0028004	0.028213512	0.990320465
mmu-miR-7051-5p	MIMAT0028006	-0.206907014	0.990320465
mmu-miR-7052-5p	MIMAT0028008	-0.021121557	0.990320465
mmu-miR-7055-5p	MIMAT0028014	0.07033518	0.990320465
mmu-miR-7056-5p	MIMAT0028016	-0.028745257	0.990320465
mmu-miR-7058-5p	MIMAT0028020	-0.24255704	0.990320465
mmu-miR-706	MIMAT0003496	-0.082150719	0.990320465
mmu-miR-7063-5p	MIMAT0028030	0.048946623	0.990320465
mmu-miR-7068-5p	MIMAT0028042	0.063944387	0.990320465
mmu-miR-7069-5p	MIMAT0028044	0.021375946	0.990320465
mmu-miR-7070-5p	MIMAT0028046	0.02783305	0.990320465
mmu-miR-7072-5p	MIMAT0028050	0.016940613	0.990320465
mmu-miR-7074-5p	MIMAT0028054	0.0138919	0.990320465
mmu-miR-7075-5p	MIMAT0028056	0.076128097	0.990320465
mmu-miR-7080-3p	MIMAT0028067	-0.129028752	0.990320465
mmu-miR-7080-5p	MIMAT0028066	0.022004638	0.990320465
mmu-miR-7082-5p	MIMAT0028070	-0.028267337	0.990320465
mmu-miR-7083-5p	MIMAT0028072	0.230847963	0.990320465
mmu-miR-7084-5p	MIMAT0028074	-0.041853796	0.990320465
mmu-miR-7085-5p	MIMAT0028076	0.012610633	0.990320465
mmu-miR-7086-5p	MIMAT0028078	0.071190595	0.990320465
mmu-miR-7087-5p	MIMAT0028080	0.874288953	0.990320465
mmu-miR-7088-5p	MIMAT0028082	0.078527998	0.990320465
mmu-miR-709	MIMAT0003499	-0.035404835	0.990320465
mmu-miR-710	MIMAT0003500	-0.042182207	0.990320465
mmu-miR-711	MIMAT0003501	-0.06992499	0.990320465
mmu-miR-7115-3p	MIMAT0028128	0.085628917	0.990320465
mmu-miR-7118-5p	MIMAT0028133	-0.029621	0.990320465
mmu-miR-712-5p	MIMAT0003502	-0.048402783	0.990320465
mmu-miR-714	MIMAT0003505	0.238796315	0.990320465
mmu-miR-721	MIMAT0003515	0.394777206	0.990320465
mmu-miR-7211-3p	MIMAT0028391	-0.017282588	0.990320465
mmu-miR-7216-5p	MIMAT0028400	0.100770194	0.990320465
mmu-miR-7218-5p	MIMAT0028404	0.072795912	0.990320465
mmu-miR-7219-5p	MIMAT0028406	-0.278345127	0.990320465
mmu-miR-7221-3p	MIMAT0028411	0.069810583	0.990320465
mmu-miR-7221-5p	MIMAT0028410	0.040361979	0.990320465
mmu-miR-7222-3p	MIMAT0028413	-0.043229959	0.990320465
mmu-miR-7224-3p	MIMAT0028417	0.023134585	0.990320465

mmu-miR-7226-5p	MIMAT0028420	0.010362737	0.990320465
mmu-miR-7233-5p	MIMAT0028434	0.076802347	0.990320465
mmu-miR-7234-3p	MIMAT0028437	0.067588908	0.990320465
mmu-miR-7235-5p	MIMAT0028438	0.252572642	0.990320465
mmu-miR-7241-3p	MIMAT0028451	0.052574655	0.990320465
mmu-miR-744-5p	MIMAT0004187	0.062594954	0.990320465
mmu-miR-760-3p	MIMAT0003898	0.105312578	0.990320465
mmu-miR-762	MIMAT0003892	0.069091616	0.990320465
mmu-miR-7647-3p	MIMAT0029797	-0.103553503	0.990320465
mmu-miR-7648-3p	MIMAT0029799	0.121226681	0.990320465
mmu-miR-7653-5p	MIMAT0029812	-0.015339101	0.990320465
mmu-miR-7658-5p	MIMAT0029822	-0.049998818	0.990320465
mmu-miR-7664-3p	MIMAT0029835	0.013137243	0.990320465
mmu-miR-7665-5p	MIMAT0029836	0.010303328	0.990320465
mmu-miR-7666-3p	MIMAT0029839	0.064305918	0.990320465
mmu-miR-7666-5p	MIMAT0029838	0.063176679	0.990320465
mmu-miR-7668-3p	MIMAT0029843	-0.095364116	0.990320465
mmu-miR-7671-3p	MIMAT0029849	0.158433078	0.990320465
mmu-miR-7672-5p	MIMAT0029850	-0.013557942	0.990320465
mmu-miR-7674-5p	MIMAT0029856	0.135085059	0.990320465
mmu-miR-7682-3p	MIMAT0029885	-0.065650721	0.990320465
mmu-miR-7684-3p	MIMAT0029891	-0.057176825	0.990320465
mmu-miR-7684-5p	MIMAT0029890	0.071417521	0.990320465
mmu-miR-7686-5p	MIMAT0029898	0.098881053	0.990320465
mmu-miR-7687-5p	MIMAT0029902	0.137848255	0.990320465
mmu-miR-770-3p	MIMAT0003891	0.005549388	0.990320465
mmu-miR-7a-5p	MIMAT0000677	-0.062228699	0.990320465
mmu-miR-8090	MIMAT0031391	-0.007689187	0.990320465
mmu-miR-8093	MIMAT0031394	0.47077	0.990320465
mmu-miR-8094	MIMAT0031395	0.081676001	0.990320465
mmu-miR-8099	MIMAT0031401	-0.022062814	0.990320465
mmu-miR-8100	MIMAT0031403	0.110232603	0.990320465
mmu-miR-8101	MIMAT0031405	0.057313331	0.990320465
mmu-miR-8102	MIMAT0031406	0.048927769	0.990320465
mmu-miR-8105	MIMAT0031409	0.053967392	0.990320465
mmu-miR-8107	MIMAT0031412	0.067926144	0.990320465
mmu-miR-8108	MIMAT0031413	-0.065449623	0.990320465
mmu-miR-8109	MIMAT0031415	0.132213344	0.990320465
mmu-miR-8110	MIMAT0031416	0.148058054	0.990320465
mmu-miR-8113	MIMAT0031419	-0.096257405	0.990320465
mmu-miR-8117	MIMAT0031423	-0.193917251	0.990320465
mmu-miR-8119	MIMAT0031425	0.066749327	0.990320465
mmu-miR-872-5p	MIMAT0004934	-0.016263313	0.990320465
mmu-miR-874-3p	MIMAT0004853	0.043931216	0.990320465
mmu-miR-877-5p	MIMAT0004861	0.092058221	0.990320465
mmu-miR-878-3p	MIMAT0004933	0.026971172	0.990320465
mmu-miR-92a-2-5p	MIMAT0004635	-0.075012747	0.990320465
mmu-miR-92a-3p	MIMAT0000539	0.048145645	0.990320465
mmu-miR-93-5p	MIMAT0000540	0.080340289	0.990320465
mmu-miR-98-5p	MIMAT0000545	-0.019728998	0.990320465
mmu-miR-99a-5p	MIMAT0000131	-0.104980064	0.990320465

mmu-miR-99b-3p	MIMAT0004525	0.028702464	0.990320465
mmu-miR-99b-5p	MIMAT0000132	0.00453047	0.990320465

Supplementary Table 3: miRNA expression profiles between 6 weeks post DMM and sham synovium samples

miRNA	miRBase Accession No.	Log_2FC	adj.p.val
mmu-let-7a-5p	MIMAT0000521	0.023241697	0.999005756
mmu-let-7b-3p	MIMAT0004621	0.765910354	0.999005756
mmu-let-7b-5p	MIMAT0000522	0.046658207	0.999005756
mmu-let-7c-5p	MIMAT0000523	0.0403827	0.999005756
mmu-let-7d-5p	MIMAT0000383	0.005982298	0.999005756
mmu-let-7e-5p	MIMAT0000524	-0.074600316	0.999005756
mmu-let-7f-5p	MIMAT0000525	0.019498823	0.999005756
mmu-let-7g-5p	MIMAT0000121	-0.014263678	0.999005756
mmu-let-7i-5p	MIMAT0000122	0.001342219	0.999005756
mmu-let-7k	MIMAT0025580	-0.044353916	0.999005756
mmu-miR-100-5p	MIMAT0000655	0.043899158	0.999005756
mmu-miR-101a-3p	MIMAT0000133	0.158387676	0.999005756
mmu-miR-101c	MIMAT0019349	0.152483049	0.999005756
mmu-miR-103-3p	MIMAT0000546	-0.102269766	0.999005756
mmu-miR-106b-5p	MIMAT0000386	0.128861854	0.999005756
mmu-miR-107-3p	MIMAT0000647	-0.013401815	0.999005756
mmu-miR-10a-5p	MIMAT0000648	-0.041314555	0.999005756
mmu-miR-10b-5p	MIMAT0000208	-0.006362068	0.999005756
mmu-miR-1187	MIMAT0005837	-0.111042649	0.999005756
mmu-miR-1188-5p	MIMAT0005843	0.044268454	0.999005756
mmu-miR-1198-5p	MIMAT0005859	0.038207005	0.999005756
mmu-miR-1224-5p	MIMAT0005460	0.243982868	0.999005756
mmu-miR-1231-5p	MIMAT0022357	0.025848654	0.999005756
mmu-miR-1249-3p	MIMAT0010560	0.054608985	0.999005756
mmu-miR-125a-3p	MIMAT0004528	-0.040875344	0.999005756
mmu-miR-125a-5p	MIMAT0000135	-0.085794033	0.999005756
mmu-miR-125b-5p	MIMAT0000136	0.071800363	0.999005756
mmu-miR-126a-3p	MIMAT0000138	-0.094014271	0.999005756
mmu-miR-126a-5p	MIMAT0000137	-0.063997174	0.999005756
mmu-miR-127-3p	MIMAT0000139	-0.08442875	0.999005756
mmu-miR-128-3p	MIMAT0000140	0.151015362	0.999005756
mmu-miR-129-5p	MIMAT0000209	-0.08479188	0.999005756
mmu-miR-129b-5p	MIMAT0029862	-0.077049108	0.999005756
mmu-miR-1306-3p	MIMAT0009411	-0.056863561	0.999005756
mmu-miR-130a-3p	MIMAT0000141	-0.089545137	0.999005756
mmu-miR-130b-3p	MIMAT0000387	0.012387492	0.999005756
mmu-miR-132-3p	MIMAT0000144	-0.043712928	0.999005756
mmu-miR-133a-3p	MIMAT0000145	0.360499111	0.999005756
mmu-miR-133a-5p	MIMAT0003473	0.29152565	0.999005756
mmu-miR-133b-3p	MIMAT0000769	0.348723289	0.999005756
mmu-miR-134-5p	MIMAT0000146	0.091974479	0.999005756
mmu-miR-135a-1-3p	MIMAT0004531	0.104018292	0.999005756
mmu-miR-136-5p	MIMAT0000148	-0.008576938	0.999005756
mmu-miR-139-3p	MIMAT0004662	0.076773747	0.999005756
mmu-miR-139-5p	MIMAT0000656	-0.049729686	0.999005756
mmu-miR-140-3p	MIMAT0000152	0.290170353	0.999005756
mmu-miR-140-5p	MIMAT0000151	0.267806544	0.999005756

mmu-miR-142a-3p	MIMAT0000155	0.128512484	0.999005756
mmu-miR-142a-5p	MIMAT0000154	0.107375134	0.999005756
mmu-miR-143-3p	MIMAT0000247	-0.128488173	0.999005756
mmu-miR-144-3p	MIMAT0000156	0.550888997	0.999005756
mmu-miR-144-5p	MIMAT0016988	0.509205012	0.999005756
mmu-miR-145a-5p	MIMAT0000157	-0.081441265	0.999005756
mmu-miR-146a-5p	MIMAT0000158	-0.021042239	0.999005756
mmu-miR-146b-5p	MIMAT0003475	-0.108370728	0.999005756
mmu-miR-148a-3p	MIMAT0000516	0.022649991	0.999005756
mmu-miR-148b-3p	MIMAT0000580	0.005768124	0.999005756
mmu-miR-149-3p	MIMAT0016990	0.086956085	0.999005756
mmu-miR-149-5p	MIMAT0000159	0.13251568	0.999005756
mmu-miR-150-3p	MIMAT0004535	0.027245329	0.999005756
mmu-miR-150-5p	MIMAT0000160	0.14750427	0.999005756
mmu-miR-151-5p	MIMAT0004536	0.048092088	0.999005756
mmu-miR-152-3p	MIMAT0000162	0.034226284	0.999005756
mmu-miR-154-3p	MIMAT0004537	-0.12538797	0.999005756
mmu-miR-154-5p	MIMAT0000164	-0.075175935	0.999005756
mmu-miR-155-5p	MIMAT0000165	-0.06065459	0.999005756
mmu-miR-15a-5p	MIMAT0000526	0.058919919	0.999005756
mmu-miR-15b-5p	MIMAT0000124	0.057393855	0.999005756
mmu-miR-16-5p	MIMAT0000527	0.059650774	0.999005756
mmu-miR-17-3p	MIMAT0000650	0.012448172	0.999005756
mmu-miR-17-5p	MIMAT0000649	0.053249722	0.999005756
mmu-miR-181a-1-3p	MIMAT0000660	0.081355642	0.999005756
mmu-miR-181a-5p	MIMAT0000210	0.157869501	0.999005756
mmu-miR-181b-5p	MIMAT0000673	0.107508781	0.999005756
mmu-miR-181c-5p	MIMAT0000674	0.052127461	0.999005756
mmu-miR-181d-5p	MIMAT0004324	-0.014211371	0.999005756
mmu-miR-1839-3p	MIMAT0009457	0.01063538	0.999005756
mmu-miR-1839-5p	MIMAT0009456	-0.037448238	0.999005756
mmu-miR-185-5p	MIMAT0000214	0.113329592	0.999005756
mmu-miR-188-5p	MIMAT0000217	0.140220245	0.999005756
mmu-miR-1892	MIMAT0007871	-0.053924395	0.999005756
mmu-miR-1894-3p	MIMAT0007878	0.003481125	0.999005756
mmu-miR-1895	MIMAT0007867	-0.015970547	0.999005756
mmu-miR-1896	MIMAT0007873	-0.021256793	0.999005756
mmu-miR-1897-3p	MIMAT0007865	0.198317299	0.999005756
mmu-miR-1897-5p	MIMAT0007864	0.158477104	0.999005756
mmu-miR-18a-5p	MIMAT0000528	0.139277512	0.999005756
mmu-miR-1901	MIMAT0007880	-0.140256599	0.999005756
mmu-miR-1904	MIMAT0007874	0.03475623	0.999005756
mmu-miR-1906	MIMAT0007872	0.072492679	0.999005756
mmu-miR-1907	MIMAT0007876	-0.106466333	0.999005756
mmu-miR-190b-5p	MIMAT0004852	-0.174838889	0.999005756
mmu-miR-191-3p	MIMAT0004542	0.179030522	0.999005756
mmu-miR-192-5p	MIMAT0000517	-0.180498548	0.999005756
mmu-miR-1927	MIMAT0009390	-0.07883053	0.999005756
mmu-miR-1930-3p	MIMAT0017340	-0.041396814	0.999005756
mmu-miR-1931	MIMAT0009394	-0.178451567	0.999005756
mmu-miR-1934-3p	MIMAT0017341	-0.009075222	0.999005756

mmu-miR-193a-3p	MIMAT0000223	-0.123641449	0.999005756
mmu-miR-193b-3p	MIMAT0004859	0.165234273	0.999005756
mmu-miR-193b-5p	MIMAT0017271	-0.074074375	0.999005756
mmu-miR-194-5p	MIMAT0000224	-0.237311131	0.999005756
mmu-miR-1941-5p	MIMAT0009405	0.02416006	0.999005756
mmu-miR-1945	MIMAT0009410	-0.110517726	0.999005756
mmu-miR-1949	MIMAT0009416	0.151547349	0.999005756
mmu-miR-1955-3p	MIMAT0017348	0.045145108	0.999005756
mmu-miR-1957a	MIMAT0009430	0.001481382	0.999005756
mmu-miR-195a-3p	MIMAT0017000	-0.088742678	0.999005756
mmu-miR-195a-5p	MIMAT0000225	0.059345598	0.999005756
mmu-miR-1960	MIMAT0009433	-0.022987651	0.999005756
mmu-miR-1966-5p	MIMAT0009439	-0.039759132	0.999005756
mmu-miR-1967	MIMAT0009440	-0.128085864	0.999005756
mmu-miR-1968-5p	MIMAT0009441	-0.136964802	0.999005756
mmu-miR-196a-5p	MIMAT0000518	0.085909259	0.999005756
mmu-miR-196b-5p	MIMAT0001081	0.094772167	0.999005756
mmu-miR-1971	MIMAT0009446	-0.077738617	0.999005756
mmu-miR-1982-5p	MIMAT0009459	0.134584676	0.999005756
mmu-miR-199a-3p	MIMAT0000230	0.031993615	0.999005756
mmu-miR-199a-5p	MIMAT0000229	0.047468291	0.999005756
mmu-miR-199b-5p	MIMAT0000672	0.022869833	0.999005756
mmu-miR-19a-3p	MIMAT0000651	0.134600228	0.999005756
mmu-miR-19b-3p	MIMAT0000513	0.074145346	0.999005756
mmu-miR-1a-3p	MIMAT0000123	0.39234857	0.999005756
mmu-miR-202-3p	MIMAT0000235	0.163790189	0.999005756
mmu-miR-203-3p	MIMAT0000236	-0.142544321	0.999005756
mmu-miR-204-5p	MIMAT0000237	-0.001167131	0.999005756
mmu-miR-206-3p	MIMAT0000239	0.931466694	0.999005756
mmu-miR-208a-5p	MIMAT0017014	0.078662218	0.999005756
mmu-miR-20a-5p	MIMAT0000529	0.056999212	0.999005756
mmu-miR-20b-5p	MIMAT0003187	0.079470315	0.999005756
mmu-miR-210-3p	MIMAT0000658	0.165012209	0.999005756
mmu-miR-211-3p	MIMAT0017059	0.095532907	0.999005756
mmu-miR-212-3p	MIMAT0000659	0.070336766	0.999005756
mmu-miR-2137	MIMAT0011213	0.139681749	0.999005756
mmu-miR-214-3p	MIMAT0000661	0.029642216	0.999005756
mmu-miR-214-5p	MIMAT0004664	0.066446393	0.999005756
mmu-miR-218-5p	MIMAT0000663	-0.065212647	0.999005756
mmu-miR-21a-3p	MIMAT0004628	-0.061011715	0.999005756
mmu-miR-21a-5p	MIMAT0000530	-0.178406527	0.999005756
mmu-miR-22-3p	MIMAT0000531	0.039771541	0.999005756
mmu-miR-22-5p	MIMAT0004629	0.08439891	0.999005756
mmu-miR-221-3p	MIMAT0000669	0.01360075	0.999005756
mmu-miR-222-3p	MIMAT0000670	0.093545556	0.999005756
mmu-miR-223-3p	MIMAT0000665	-0.115176266	0.999005756
mmu-miR-224-5p	MIMAT0000671	-0.072346798	0.999005756
mmu-miR-23a-3p	MIMAT0000532	0.050568678	0.999005756
mmu-miR-23a-5p	MIMAT0017019	-0.037537233	0.999005756
mmu-miR-23b-3p	MIMAT0000125	0.085612354	0.999005756
mmu-miR-24-2-5p	MIMAT0005440	0.033936657	0.999005756

mmu-miR-24-3p	MIMAT0000219	0.017910348	0.999005756
mmu-miR-25-3p	MIMAT0000652	0.145202716	0.999005756
mmu-miR-26a-5p	MIMAT0000533	0.053581599	0.999005756
mmu-miR-26b-5p	MIMAT0000534	0.084821167	0.999005756
mmu-miR-27a-3p	MIMAT0000537	0.020445496	0.999005756
mmu-miR-27b-3p	MIMAT0000126	0.083328123	0.999005756
mmu-miR-2861	MIMAT0013803	0.135300533	0.999005756
mmu-miR-28a-5p	MIMAT0000653	0.011782221	0.999005756
mmu-miR-28c	MIMAT0019339	-0.044131271	0.999005756
mmu-miR-290a-5p	MIMAT0000366	0.105468425	0.999005756
mmu-miR-292a-5p	MIMAT0000369	0.076108567	0.999005756
mmu-miR-295-5p	MIMAT0004575	-0.048382335	0.999005756
mmu-miR-296-5p	MIMAT0000374	0.063281581	0.999005756
mmu-miR-299a-3p	MIMAT0004577	-0.075915153	0.999005756
mmu-miR-299b-5p	MIMAT0022836	-0.047892565	0.999005756
mmu-miR-29a-3p	MIMAT0000535	0.053742358	0.999005756
mmu-miR-29b-1-5p	MIMAT0004523	0.032276392	0.999005756
mmu-miR-29b-3p	MIMAT0000127	0.051474727	0.999005756
mmu-miR-29c-3p	MIMAT0000536	0.105457923	0.999005756
mmu-miR-29c-5p	MIMAT0004632	0.016446062	0.999005756
mmu-miR-300-3p	MIMAT0000378	-0.068764524	0.999005756
mmu-miR-301a-3p	MIMAT0000379	0.020258791	0.999005756
mmu-miR-302c-5p	MIMAT0003375	-0.130676013	0.999005756
mmu-miR-3058-3p	MIMAT0014814	-0.007284375	0.999005756
mmu-miR-3058-5p	MIMAT0014813	0.015186037	0.999005756
mmu-miR-3059-3p	MIMAT0014812	-0.057746124	0.999005756
mmu-miR-3067-3p	MIMAT0014841	0.082119661	0.999005756
mmu-miR-3069-3p	MIMAT0014845	0.010142802	0.999005756
mmu-miR-3070-2-3p	MIMAT0014849	-0.080443113	0.999005756
mmu-miR-3072-5p	MIMAT0014852	0.021205883	0.999005756
mmu-miR-3075-5p	MIMAT0014858	-0.087052492	0.999005756
mmu-miR-3077-5p	MIMAT0014862	0.049708158	0.999005756
mmu-miR-3081-5p	MIMAT0014870	0.049302212	0.999005756
mmu-miR-3082-5p	MIMAT0014872	0.007676298	0.999005756
mmu-miR-3085-3p	MIMAT0014879	0.028525282	0.999005756
mmu-miR-3085-5p	MIMAT0014878	0.06391976	0.999005756
mmu-miR-3092-3p	MIMAT0014906	0.060734957	0.999005756
mmu-miR-3093-3p	MIMAT0014908	0.034959488	0.999005756
mmu-miR-3095-3p	MIMAT0014912	0.072996757	0.999005756
mmu-miR-3098-5p	MIMAT0014917	-0.076587426	0.999005756
mmu-miR-3099-3p	MIMAT0014816	0.007050537	0.999005756
mmu-miR-30a-3p	MIMAT0000129	0.057151591	0.999005756
mmu-miR-30a-5p	MIMAT0000128	0.037372233	0.999005756
mmu-miR-30b-3p	MIMAT0004524	-0.022830022	0.999005756
mmu-miR-30b-5p	MIMAT0000130	0.076762437	0.999005756
mmu-miR-30c-1-3p	MIMAT0004616	-0.022382918	0.999005756
mmu-miR-30c-2-3p	MIMAT0005438	-0.017321283	0.999005756
mmu-miR-30c-5p	MIMAT0000514	0.072278197	0.999005756
mmu-miR-30d-5p	MIMAT0000515	0.07263751	0.999005756
mmu-miR-30e-3p	MIMAT0000249	0.079967235	0.999005756
mmu-miR-30e-5p	MIMAT0000248	0.081762068	0.999005756

mmu-miR-31-5p	MIMAT0000538	-0.215000576	0.999005756
mmu-miR-3101-3p	MIMAT0014922	-0.082471544	0.999005756
mmu-miR-3102-3p	MIMAT0014936	0.13002269	0.999005756
mmu-miR-3102-5p	MIMAT0014933	0.072902834	0.999005756
mmu-miR-3102-5p.2-5p	MIMAT0014934	-0.015294357	0.999005756
mmu-miR-3110-3p	MIMAT0014952	0.044522617	0.999005756
mmu-miR-3154	MIMAT0035714	0.247886722	0.999005756
mmu-miR-320-3p	MIMAT0000666	0.094076018	0.999005756
mmu-miR-322-5p	MIMAT0000548	-0.187261874	0.999005756
mmu-miR-324-3p	MIMAT0000556	0.06819883	0.999005756
mmu-miR-324-5p	MIMAT0000555	0.001937216	0.999005756
mmu-miR-327	MIMAT0004867	-0.033706423	0.999005756
mmu-miR-328-5p	MIMAT0017030	-0.148920147	0.999005756
mmu-miR-329-3p	MIMAT0000567	-0.086549114	0.999005756
mmu-miR-331-3p	MIMAT0000571	0.04274989	0.999005756
mmu-miR-335-5p	MIMAT0000766	-0.177488952	0.999005756
mmu-miR-337-3p	MIMAT0000578	-0.069263705	0.999005756
mmu-miR-337-5p	MIMAT0004644	-0.136199599	0.999005756
mmu-miR-338-3p	MIMAT0000582	-0.018588838	0.999005756
mmu-miR-338-5p	MIMAT0004647	-0.031018659	0.999005756
mmu-miR-340-5p	MIMAT0004651	-0.009818401	0.999005756
mmu-miR-341-3p	MIMAT0000588	-0.060583321	0.999005756
mmu-miR-342-3p	MIMAT0000590	-0.036585398	0.999005756
mmu-miR-344i	MIMAT0022503	0.175372277	0.999005756
mmu-miR-345-5p	MIMAT0000595	0.045663697	0.999005756
mmu-miR-3470a	MIMAT0015640	-0.050877369	0.999005756
mmu-miR-3472	MIMAT0015643	-0.362053035	0.999005756
mmu-miR-3473a	MIMAT0015645	-0.117037343	0.999005756
mmu-miR-3473b	MIMAT0020367	-0.249426023	0.999005756
mmu-miR-3473e	MIMAT0025587	0.038359851	0.999005756
mmu-miR-3473f	MIMAT0031390	0.170669941	0.999005756
mmu-miR-3473g	MIMAT0031427	0.084221638	0.999005756
mmu-miR-3474	MIMAT0015646	0.136190141	0.999005756
mmu-miR-34a-5p	MIMAT0000542	-0.016672257	0.999005756
mmu-miR-34b-5p	MIMAT0000382	-0.087173251	0.999005756
mmu-miR-34c-5p	MIMAT0000381	-0.12218914	0.999005756
mmu-miR-350-3p	MIMAT0000605	-0.001756685	0.999005756
mmu-miR-3535	MIMAT0031410	0.025440306	0.999005756
mmu-miR-3544-3p	MIMAT0022354	0.158402493	0.999005756
mmu-miR-3547-5p	MIMAT0027832	0.137127954	0.999005756
mmu-miR-3572-5p	MIMAT0022986	0.008154019	0.999005756
mmu-miR-361-5p	MIMAT0000704	0.047169583	0.999005756
mmu-miR-362-3p	MIMAT0004684	-0.075684621	0.999005756
mmu-miR-362-5p	MIMAT0000706	-0.172468898	0.999005756
mmu-miR-3620-3p	MIMAT0029879	0.085293414	0.999005756
mmu-miR-3620-5p	MIMAT0029878	0.11112447	0.999005756
mmu-miR-365-1-5p	MIMAT0017077	-0.027694794	0.999005756
mmu-miR-365-3p	MIMAT0000711	0.03617451	0.999005756
mmu-miR-370-3p	MIMAT0001095	0.133403493	0.999005756
mmu-miR-374c-5p	MIMAT0014953	0.091100073	0.999005756
mmu-miR-376a-3p	MIMAT0000740	-0.085246885	0.999005756

mmu-miR-376b-3p	MIMAT0001092	-0.079262198	0.999005756
mmu-miR-376c-3p	MIMAT0003183	-0.090638878	0.999005756
mmu-miR-377-3p	MIMAT0000741	-0.115404709	0.999005756
mmu-miR-378a-3p	MIMAT0003151	0.155787021	0.999005756
mmu-miR-378a-5p	MIMAT0000742	0.14671176	0.999005756
mmu-miR-378b	MIMAT0019348	0.16290575	0.999005756
mmu-miR-378c	MIMAT0025138	0.14874604	0.999005756
mmu-miR-378d	MIMAT0025167	0.171328812	0.999005756
mmu-miR-379-5p	MIMAT0000743	-0.117146884	0.999005756
mmu-miR-381-3p	MIMAT0000746	-0.004852431	0.999005756
mmu-miR-382-5p	MIMAT0000747	-0.06415319	0.999005756
mmu-miR-3960	MIMAT0019336	0.185293348	0.999005756
mmu-miR-3963	MIMAT0019341	-0.116309036	0.999005756
mmu-miR-3968	MIMAT0019352	0.177792981	0.999005756
mmu-miR-409-3p	MIMAT0001090	-0.062825833	0.999005756
mmu-miR-410-3p	MIMAT0001091	-0.11023215	0.999005756
mmu-miR-411-3p	MIMAT0001093	-0.029257325	0.999005756
mmu-miR-411-5p	MIMAT0004747	-0.070329914	0.999005756
mmu-miR-423-5p	MIMAT0004825	0.031614859	0.999005756
mmu-miR-425-5p	MIMAT0004750	0.016993832	0.999005756
mmu-miR-431-5p	MIMAT0001418	-0.036922491	0.999005756
mmu-miR-432	MIMAT0012771	0.011864451	0.999005756
mmu-miR-434-3p	MIMAT0001422	-0.057130544	0.999005756
mmu-miR-434-5p	MIMAT0001421	-0.076937533	0.999005756
mmu-miR-450a-2-3p	MIMAT0004789	0.003506241	0.999005756
mmu-miR-451a	MIMAT0001632	0.472777923	0.999005756
mmu-miR-452-5p	MIMAT0001637	-0.027051219	0.999005756
mmu-miR-455-3p	MIMAT0003742	0.218404353	0.999005756
mmu-miR-455-5p	MIMAT0003485	0.061582981	0.999005756
mmu-miR-466f-3p	MIMAT0004882	-0.079923091	0.999005756
mmu-miR-466g	MIMAT0004883	-0.044075848	0.999005756
mmu-miR-466h-3p	MIMAT0017274	-0.026732976	0.999005756
mmu-miR-466i-3p	MIMAT0005834	-0.048408016	0.999005756
mmu-miR-466i-5p	MIMAT0017325	-0.115305619	0.999005756
mmu-miR-466m-3p	MIMAT0014883	-0.043553344	0.999005756
mmu-miR-466q	MIMAT0020631	0.011628712	0.999005756
mmu-miR-467a-3p	MIMAT0002108	-0.064847277	0.999005756
mmu-miR-467f	MIMAT0005846	-0.017777844	0.999005756
mmu-miR-468-3p	MIMAT0002109	-0.075007358	0.999005756
mmu-miR-470-5p	MIMAT0002111	0.089763289	0.999005756
mmu-miR-483-3p	MIMAT0003120	0.15942869	0.999005756
mmu-miR-483-5p	MIMAT0004782	0.084604412	0.999005756
mmu-miR-484	MIMAT0003127	-0.084955653	0.999005756
mmu-miR-486a-3p	MIMAT0017206	0.100028604	0.999005756
mmu-miR-486a-5p	MIMAT0003130	0.30594101	0.999005756
mmu-miR-487b-3p	MIMAT0003184	0.046862054	0.999005756
mmu-miR-490-5p	MIMAT0017261	0.118367683	0.999005756
mmu-miR-494-3p	MIMAT0003182	0.09280535	0.999005756
mmu-miR-495-3p	MIMAT0003456	-0.070676368	0.999005756
mmu-miR-497a-5p	MIMAT0003453	0.067659692	0.999005756
mmu-miR-500-3p	MIMAT0003507	-0.095725278	0.999005756

mmu-miR-503-5p	MIMAT0003188	-0.030532216	0.999005756
mmu-miR-504-3p	MIMAT0017277	-0.069509486	0.999005756
mmu-miR-5099	MIMAT0020606	0.179434584	0.999005756
mmu-miR-5100	MIMAT0020607	-0.056566072	0.999005756
mmu-miR-5103	MIMAT0020610	0.080672793	0.999005756
mmu-miR-5104	MIMAT0020611	0.070035273	0.999005756
mmu-miR-5107-5p	MIMAT0020615	0.068571534	0.999005756
mmu-miR-511-3p	MIMAT0017281	-0.186739958	0.999005756
mmu-miR-5110	MIMAT0020618	-0.023282372	0.999005756
mmu-miR-5112	MIMAT0020620	0.009278352	0.999005756
mmu-miR-5113	MIMAT0020621	0.043646204	0.999005756
mmu-miR-5118	MIMAT0020626	0.056577346	0.999005756
mmu-miR-5119	MIMAT0020627	0.081872725	0.999005756
mmu-miR-5121	MIMAT0020629	-0.041565074	0.999005756
mmu-miR-5122	MIMAT0020630	-0.033280299	0.999005756
mmu-miR-5126	MIMAT0020637	0.013377724	0.999005756
mmu-miR-5128	MIMAT0020639	-0.00763541	0.999005756
mmu-miR-5130	MIMAT0020641	0.056890892	0.999005756
mmu-miR-5131	MIMAT0020642	0.093634263	0.999005756
mmu-miR-5132-5p	MIMAT0020643	-0.02853981	0.999005756
mmu-miR-5135	MIMAT0020646	-0.052758572	0.999005756
mmu-miR-532-3p	MIMAT0004781	-0.097697405	0.999005756
mmu-miR-532-5p	MIMAT0002889	-0.110789454	0.999005756
mmu-miR-541-5p	MIMAT0003170	-0.060861111	0.999005756
mmu-miR-546	MIMAT0003166	0.086410528	0.999005756
mmu-miR-551b-5p	MIMAT0017236	-0.060512074	0.999005756
mmu-miR-5620-3p	MIMAT0022368	0.007027096	0.999005756
mmu-miR-5622-3p	MIMAT0022372	-0.159783921	0.999005756
mmu-miR-574-3p	MIMAT0004894	-0.028775548	0.999005756
mmu-miR-574-5p	MIMAT0004893	-0.050477177	0.999005756
mmu-miR-615-3p	MIMAT0003783	-0.001372787	0.999005756
mmu-miR-6236	MIMAT0024857	0.019010112	0.999005756
mmu-miR-6238	MIMAT0024859	0.00962815	0.999005756
mmu-miR-6349	MIMAT0025092	0.046585667	0.999005756
mmu-miR-6351	MIMAT0025094	-0.006444575	0.999005756
mmu-miR-6354	MIMAT0025097	0.000508458	0.999005756
mmu-miR-6360	MIMAT0025103	0.077627371	0.999005756
mmu-miR-6366	MIMAT0025110	0.13522499	0.999005756
mmu-miR-6368	MIMAT0025112	-0.000116913	0.999005756
mmu-miR-6370	MIMAT0025114	0.02748806	0.999005756
mmu-miR-6378	MIMAT0025124	-0.010739725	0.999005756
mmu-miR-6385	MIMAT0025131	0.076914124	0.999005756
mmu-miR-6386	MIMAT0025133	0.014323745	0.999005756
mmu-miR-6388	MIMAT0025135	-0.045255337	0.999005756
mmu-miR-6391	MIMAT0025140	0.022552626	0.999005756
mmu-miR-6392-3p	MIMAT0025142	-0.331711802	0.999005756
mmu-miR-6394	MIMAT0025144	0.05399725	0.999005756
mmu-miR-6401	MIMAT0025153	-0.089267324	0.999005756
mmu-miR-6402	MIMAT0025154	-0.026751776	0.999005756
mmu-miR-6405	MIMAT0025157	0.023256124	0.999005756
mmu-miR-6407	MIMAT0025160	0.058823119	0.999005756

mmu-miR-6412	MIMAT0025165	0.051807174	0.999005756
mmu-miR-6418-5p	MIMAT0025173	-0.046310511	0.999005756
mmu-miR-652-3p	MIMAT0003711	-0.038431886	0.999005756
mmu-miR-652-5p	MIMAT0017260	0.141463373	0.999005756
mmu-miR-6538	MIMAT0025583	0.123782031	0.999005756
mmu-miR-664-5p	MIMAT0017353	-0.076622554	0.999005756
mmu-miR-669c-3p	MIMAT0017253	0.01287886	0.999005756
mmu-miR-669f-3p	MIMAT0005839	0.000445461	0.999005756
mmu-miR-669h-3p	MIMAT0005842	-0.05571426	0.999005756
mmu-miR-669l-5p	MIMAT0009418	-0.037124522	0.999005756
mmu-miR-669n	MIMAT0009427	-0.053841879	0.999005756
mmu-miR-669p-3p	MIMAT0014890	0.011981489	0.999005756
mmu-miR-671-5p	MIMAT0003731	0.119792594	0.999005756
mmu-miR-674-3p	MIMAT0003741	0.006257209	0.999005756
mmu-miR-674-5p	MIMAT0003740	0.013370399	0.999005756
mmu-miR-676-3p	MIMAT0003782	-0.007026746	0.999005756
mmu-miR-6769b-5p	MIMAT0028040	0.121737031	0.999005756
mmu-miR-677-3p	MIMAT0017246	0.096272056	0.999005756
mmu-miR-678	MIMAT0003452	0.007697341	0.999005756
mmu-miR-680	MIMAT0003457	0.268266885	0.999005756
mmu-miR-681	MIMAT0003458	0.043095359	0.999005756
mmu-miR-682	MIMAT0003459	-0.115247677	0.999005756
mmu-miR-6898-5p	MIMAT0027696	-0.105076119	0.999005756
mmu-miR-690	MIMAT0003469	-0.065644455	0.999005756
mmu-miR-6901-5p	MIMAT0027702	0.108571387	0.999005756
mmu-miR-6904-5p	MIMAT0027708	-0.046295521	0.999005756
mmu-miR-6905-5p	MIMAT0027710	-0.132817184	0.999005756
mmu-miR-6906-5p	MIMAT0027712	0.035123842	0.999005756
mmu-miR-6908-5p	MIMAT0027716	0.112903764	0.999005756
mmu-miR-6909-5p	MIMAT0027718	0.039581773	0.999005756
mmu-miR-691	MIMAT0003470	0.012788625	0.999005756
mmu-miR-6910-5p	MIMAT0027720	0.008660326	0.999005756
mmu-miR-6911-5p	MIMAT0027722	-0.082967177	0.999005756
mmu-miR-6912-5p	MIMAT0027724	0.051514528	0.999005756
mmu-miR-6915-5p	MIMAT0027730	0.004450448	0.999005756
mmu-miR-6918-5p	MIMAT0027736	-0.061985872	0.999005756
mmu-miR-6921-5p	MIMAT0027742	-0.03333047	0.999005756
mmu-miR-6922-5p	MIMAT0027744	-0.002980243	0.999005756
mmu-miR-6923-5p	MIMAT0027746	-0.061537783	0.999005756
mmu-miR-6926-5p	MIMAT0027752	-0.047205919	0.999005756
mmu-miR-6931-5p	MIMAT0027762	-0.014573586	0.999005756
mmu-miR-6934-5p	MIMAT0027768	0.005823244	0.999005756
mmu-miR-6937-5p	MIMAT0027774	0.018372393	0.999005756
mmu-miR-6939-5p	MIMAT0027778	-0.022341381	0.999005756
mmu-miR-6944-5p	MIMAT0027788	0.070837344	0.999005756
mmu-miR-6946-5p	MIMAT0027792	-0.075383868	0.999005756
mmu-miR-6952-5p	MIMAT0027804	0.162874632	0.999005756
mmu-miR-6954-5p	MIMAT0027808	0.039135255	0.999005756
mmu-miR-6955-5p	MIMAT0027810	-0.108622254	0.999005756
mmu-miR-6956-5p	MIMAT0027812	-0.045817529	0.999005756
mmu-miR-6957-5p	MIMAT0027814	0.007492866	0.999005756

mmu-miR-6959-5p	MIMAT0027818	0.057745981	0.999005756
mmu-miR-6961-5p	MIMAT0027822	-0.019627669	0.999005756
mmu-miR-6963-5p	MIMAT0027826	-0.009524541	0.999005756
mmu-miR-6965-5p	MIMAT0027830	0.108935834	0.999005756
mmu-miR-6969-5p	MIMAT0027840	0.018341427	0.999005756
mmu-miR-697	MIMAT0003487	0.121418822	0.999005756
mmu-miR-6970-5p	MIMAT0027842	0.015422732	0.999005756
mmu-miR-6971-5p	MIMAT0027844	-0.045048144	0.999005756
mmu-miR-6972-5p	MIMAT0027846	-0.000470604	0.999005756
mmu-miR-6973a-5p	MIMAT0027848	0.014327727	0.999005756
mmu-miR-6973b-5p	MIMAT0027908	0.041574147	0.999005756
mmu-miR-6974-5p	MIMAT0027850	0.081696008	0.999005756
mmu-miR-6975-5p	MIMAT0027852	-0.155066685	0.999005756
mmu-miR-6976-3p	MIMAT0027855	0.103326708	0.999005756
mmu-miR-6978-5p	MIMAT0027858	0.01794549	0.999005756
mmu-miR-698-5p	MIMAT0022930	-0.06180937	0.999005756
mmu-miR-6980-5p	MIMAT0027862	-0.078623581	0.999005756
mmu-miR-6981-5p	MIMAT0027864	-0.045718371	0.999005756
mmu-miR-6982-5p	MIMAT0027866	0.022073225	0.999005756
mmu-miR-6984-3p	MIMAT0027871	-0.113281601	0.999005756
mmu-miR-6984-5p	MIMAT0027870	-0.006124168	0.999005756
mmu-miR-6987-5p	MIMAT0027876	-0.008630839	0.999005756
mmu-miR-6988-5p	MIMAT0027878	0.028486899	0.999005756
mmu-miR-6990-5p	MIMAT0027882	-0.001436602	0.999005756
mmu-miR-6991-5p	MIMAT0027884	-0.015802209	0.999005756
mmu-miR-6995-5p	MIMAT0027892	0.033530307	0.999005756
mmu-miR-6997-5p	MIMAT0027896	0.096247694	0.999005756
mmu-miR-6998-5p	MIMAT0027898	0.028323903	0.999005756
mmu-miR-6999-5p	MIMAT0027900	-0.006920416	0.999005756
mmu-miR-700-3p	MIMAT0003490	-0.117622275	0.999005756
mmu-miR-7002-5p	MIMAT0027906	0.070547104	0.999005756
mmu-miR-7005-5p	MIMAT0027914	-0.028359817	0.999005756
mmu-miR-7007-5p	MIMAT0027918	0.014281329	0.999005756
mmu-miR-7008-5p	MIMAT0027920	-0.023564287	0.999005756
mmu-miR-7009-5p	MIMAT0027922	0.060496254	0.999005756
mmu-miR-7011-5p	MIMAT0027926	-0.029660564	0.999005756
mmu-miR-7012-5p	MIMAT0027928	0.039548755	0.999005756
mmu-miR-7014-5p	MIMAT0027932	0.098716322	0.999005756
mmu-miR-7015-5p	MIMAT0027934	0.144480182	0.999005756
mmu-miR-7016-5p	MIMAT0027936	-0.031685215	0.999005756
mmu-miR-7018-5p	MIMAT0027940	0.009548306	0.999005756
mmu-miR-7019-5p	MIMAT0027942	-0.020222567	0.999005756
mmu-miR-702-3p	MIMAT0003492	0.057321386	0.999005756
mmu-miR-7020-5p	MIMAT0027944	0.018670291	0.999005756
mmu-miR-7022-3p	MIMAT0027949	0.070448964	0.999005756
mmu-miR-7025-5p	MIMAT0027954	-0.07191084	0.999005756
mmu-miR-7027-5p	MIMAT0027958	-0.081210956	0.999005756
mmu-miR-7028-5p	MIMAT0027960	-0.126325102	0.999005756
mmu-miR-7031-5p	MIMAT0027966	0.059177886	0.999005756
mmu-miR-7032-5p	MIMAT0027968	0.046281112	0.999005756
mmu-miR-7033-5p	MIMAT0027970	0.047445611	0.999005756

mmu-miR-7034-5p	MIMAT0027972	-0.012934701	0.999005756
mmu-miR-7035-5p	MIMAT0027974	0.010358633	0.999005756
mmu-miR-7036a-5p	MIMAT0027976	0.158509369	0.999005756
mmu-miR-7040-5p	MIMAT0027984	0.080216519	0.999005756
mmu-miR-7042-5p	MIMAT0027988	-0.064378942	0.999005756
mmu-miR-7043-5p	MIMAT0027990	0.005056438	0.999005756
mmu-miR-7044-5p	MIMAT0027992	-0.019870201	0.999005756
mmu-miR-7045-5p	MIMAT0027994	0.046444651	0.999005756
mmu-miR-7046-5p	MIMAT0027996	0.139881288	0.999005756
mmu-miR-7047-5p	MIMAT0027998	0.063053644	0.999005756
mmu-miR-7048-5p	MIMAT0028000	0.017898695	0.999005756
mmu-miR-705	MIMAT0003495	0.074429675	0.999005756
mmu-miR-7050-5p	MIMAT0028004	0.098386115	0.999005756
mmu-miR-7051-5p	MIMAT0028006	0.059375158	0.999005756
mmu-miR-7052-5p	MIMAT0028008	0.092027736	0.999005756
mmu-miR-7055-5p	MIMAT0028014	-0.142928633	0.999005756
mmu-miR-7056-5p	MIMAT0028016	0.028907026	0.999005756
mmu-miR-7058-5p	MIMAT0028020	0.092717136	0.999005756
mmu-miR-706	MIMAT0003496	-0.086975335	0.999005756
mmu-miR-7063-5p	MIMAT0028030	0.023615421	0.999005756
mmu-miR-7068-5p	MIMAT0028042	0.063428462	0.999005756
mmu-miR-7069-5p	MIMAT0028044	0.018320637	0.999005756
mmu-miR-7070-5p	MIMAT0028046	0.112850793	0.999005756
mmu-miR-7072-5p	MIMAT0028050	0.001579693	0.999005756
mmu-miR-7074-5p	MIMAT0028054	-0.192100344	0.999005756
mmu-miR-7075-5p	MIMAT0028056	0.032969748	0.999005756
mmu-miR-7080-3p	MIMAT0028067	0.338435712	0.999005756
mmu-miR-7080-5p	MIMAT0028066	-0.12335563	0.999005756
mmu-miR-7082-5p	MIMAT0028070	0.000223115	0.999005756
mmu-miR-7083-5p	MIMAT0028072	0.048213999	0.999005756
mmu-miR-7084-5p	MIMAT0028074	-0.041402516	0.999005756
mmu-miR-7085-5p	MIMAT0028076	0.050066949	0.999005756
mmu-miR-7086-5p	MIMAT0028078	-0.128454636	0.999005756
mmu-miR-7087-5p	MIMAT0028080	0.035062129	0.999005756
mmu-miR-7088-5p	MIMAT0028082	0.043498906	0.999005756
mmu-miR-709	MIMAT0003499	-0.057268451	0.999005756
mmu-miR-710	MIMAT0003500	-0.013475049	0.999005756
mmu-miR-711	MIMAT0003501	-0.029596015	0.999005756
mmu-miR-7115-3p	MIMAT0028128	-0.002679504	0.999005756
mmu-miR-7118-5p	MIMAT0028133	-0.018994458	0.999005756
mmu-miR-712-5p	MIMAT0003502	0.042286304	0.999005756
mmu-miR-714	MIMAT0003505	-0.143771177	0.999005756
mmu-miR-721	MIMAT0003515	-0.00886459	0.999005756
mmu-miR-7211-3p	MIMAT0028391	-0.093721208	0.999005756
mmu-miR-7216-5p	MIMAT0028400	0.067887672	0.999005756
mmu-miR-7218-5p	MIMAT0028404	-0.070023946	0.999005756
mmu-miR-7219-5p	MIMAT0028406	-0.112486152	0.999005756
mmu-miR-7221-3p	MIMAT0028411	0.099762613	0.999005756
mmu-miR-7221-5p	MIMAT0028410	0.088062825	0.999005756
mmu-miR-7222-3p	MIMAT0028413	-0.017518017	0.999005756
mmu-miR-7224-3p	MIMAT0028417	0.045832599	0.999005756

mmu-miR-7226-5p	MIMAT0028420	0.070392268	0.999005756
mmu-miR-7233-5p	MIMAT0028434	-0.069847577	0.999005756
mmu-miR-7234-3p	MIMAT0028437	-0.134948087	0.999005756
mmu-miR-7235-5p	MIMAT0028438	-0.20736231	0.999005756
mmu-miR-7241-3p	MIMAT0028451	0.055907134	0.999005756
mmu-miR-744-5p	MIMAT0004187	0.034002181	0.999005756
mmu-miR-760-3p	MIMAT0003898	0.028932559	0.999005756
mmu-miR-762	MIMAT0003892	-0.005652454	0.999005756
mmu-miR-7647-3p	MIMAT0029797	-0.011298494	0.999005756
mmu-miR-7648-3p	MIMAT0029799	0.262429829	0.999005756
mmu-miR-7653-5p	MIMAT0029812	0.014630586	0.999005756
mmu-miR-7658-5p	MIMAT0029822	0.005705937	0.999005756
mmu-miR-7664-3p	MIMAT0029835	-0.159491547	0.999005756
mmu-miR-7665-5p	MIMAT0029836	-0.032302292	0.999005756
mmu-miR-7666-3p	MIMAT0029839	0.089454483	0.999005756
mmu-miR-7666-5p	MIMAT0029838	0.07349711	0.999005756
mmu-miR-7668-3p	MIMAT0029843	-0.048993522	0.999005756
mmu-miR-7671-3p	MIMAT0029849	-0.057514791	0.999005756
mmu-miR-7672-5p	MIMAT0029850	0.066107112	0.999005756
mmu-miR-7674-5p	MIMAT0029856	-0.066275193	0.999005756
mmu-miR-7682-3p	MIMAT0029885	-0.169915039	0.999005756
mmu-miR-7684-3p	MIMAT0029891	-0.0720925	0.999005756
mmu-miR-7684-5p	MIMAT0029890	0.142311022	0.999005756
mmu-miR-7686-5p	MIMAT0029898	0.101398875	0.999005756
mmu-miR-7687-5p	MIMAT0029902	-0.093743267	0.999005756
mmu-miR-770-3p	MIMAT0003891	0.094079607	0.999005756
mmu-miR-7a-5p	MIMAT0000677	-0.003476021	0.999005756
mmu-miR-8090	MIMAT0031391	-0.005754073	0.999005756
mmu-miR-8093	MIMAT0031394	0.128819079	0.999005756
mmu-miR-8094	MIMAT0031395	0.027434758	0.999005756
mmu-miR-8099	MIMAT0031401	0.049168916	0.999005756
mmu-miR-8100	MIMAT0031403	0.022151183	0.999005756
mmu-miR-8101	MIMAT0031405	-0.061484077	0.999005756
mmu-miR-8102	MIMAT0031406	0.064011941	0.999005756
mmu-miR-8105	MIMAT0031409	0.226364096	0.999005756
mmu-miR-8107	MIMAT0031412	0.02192006	0.999005756
mmu-miR-8108	MIMAT0031413	-0.114609713	0.999005756
mmu-miR-8109	MIMAT0031415	0.125831884	0.999005756
mmu-miR-8110	MIMAT0031416	0.137454167	0.999005756
mmu-miR-8113	MIMAT0031419	-0.023305102	0.999005756
mmu-miR-8117	MIMAT0031423	-0.01263458	0.999005756
mmu-miR-8119	MIMAT0031425	-0.058484068	0.999005756
mmu-miR-872-5p	MIMAT0004934	-0.020101841	0.999005756
mmu-miR-874-3p	MIMAT0004853	0.111635099	0.999005756
mmu-miR-877-5p	MIMAT0004861	0.138790659	0.999005756
mmu-miR-878-3p	MIMAT0004933	0.07881582	0.999005756
mmu-miR-92a-2-5p	MIMAT0004635	-0.029381431	0.999005756
mmu-miR-92a-3p	MIMAT0000539	0.073470885	0.999005756
mmu-miR-93-5p	MIMAT0000540	0.136677404	0.999005756
mmu-miR-98-5p	MIMAT0000545	0.10858455	0.999005756
mmu-miR-99a-5p	MIMAT0000131	-0.012394065	0.999005756

mmu-miR-99b-3p	MIMAT0004525	0.044337773	0.999005756
mmu-miR-99b-5p	MIMAT0000132	-0.060816867	0.999005756

Supplementary Table 4: Differentially expressed miRNAs in 6 weeks vs 1 week synovium samples (adj.p.value <0.05)

miRNA	miRBase Accession No.	Log_2FC	adj.P.Val
mmu-miR-1a-3p	MIMAT0000123	3.854905934	0.000202028
mmu-miR-133a-3p	MIMAT0000145	2.611995273	0.004289374
mmu-miR-101c	MIMAT0019349	2.481334751	2.59E-07
mmu-miR-101a-3p	MIMAT0000133	2.480364078	2.59E-07
mmu-miR-451a	MIMAT0001632	2.455017788	0.000584209
mmu-miR-144-3p	MIMAT0000156	2.21616346	0.001034398
mmu-miR-29c-3p	MIMAT0000536	2.180908647	3.82E-07
mmu-miR-133a-5p	MIMAT0003473	2.148818271	0.000531154
mmu-miR-195a-5p	MIMAT0000225	2.025572497	4.25E-07
mmu-miR-1941-5p	MIMAT0009405	1.936025684	0.000262539
mmu-miR-5128	MIMAT0020639	1.846364855	0.002264527
mmu-miR-23b-3p	MIMAT0000125	1.836609129	9.76E-06
mmu-miR-133b-3p	MIMAT0000769	1.825890694	0.028916507
mmu-miR-7087-5p	MIMAT0028080	1.805417371	0.045677333
mmu-miR-5121	MIMAT0020629	1.788167346	3.34E-05
mmu-miR-26a-5p	MIMAT0000533	1.769223132	6.78E-07
mmu-let-7c-5p	MIMAT0000523	1.663393736	2.59E-07
mmu-miR-30a-5p	MIMAT0000128	1.649101916	1.81E-06
mmu-miR-181a-5p	MIMAT0000210	1.625439697	0.000319411
mmu-miR-3473e	MIMAT0025587	1.590000388	8.96E-05
mmu-miR-30c-5p	MIMAT0000514	1.576527552	1.21E-05
mmu-miR-26b-5p	MIMAT0000534	1.56585168	7.34E-06
mmu-miR-8093	MIMAT0031394	1.553851287	0.001748998
mmu-miR-23a-3p	MIMAT0000532	1.502072746	6.95E-06
mmu-miR-29a-3p	MIMAT0000535	1.469520827	2.18E-06
mmu-miR-486a-5p	MIMAT0003130	1.455534286	0.001933512
mmu-miR-486a-3p	MIMAT0017206	1.412390486	3.48E-05
mmu-let-7b-5p	MIMAT0000522	1.410688904	3.80E-07
mmu-miR-181c-5p	MIMAT0000674	1.403619347	6.64E-07
mmu-miR-150-5p	MIMAT0000160	1.375202222	5.83E-05
mmu-miR-144-5p	MIMAT0016988	1.343555897	0.003114256
mmu-miR-15a-5p	MIMAT0000526	1.325873827	5.71E-06
mmu-miR-30a-3p	MIMAT0000129	1.319371704	1.64E-06
mmu-miR-125b-5p	MIMAT0000136	1.295849711	2.34E-05
mmu-miR-203-3p	MIMAT0000236	1.267163907	2.59E-07
mmu-miR-7051-5p	MIMAT0028006	1.243520264	0.000130576
mmu-miR-7020-5p	MIMAT0027944	1.239166717	0.001034398
mmu-miR-1960	MIMAT0009433	1.223652853	0.001165658
mmu-miR-709	MIMAT0003499	1.222916215	0.0033144
mmu-miR-338-3p	MIMAT0000582	1.222171425	3.93E-06
mmu-miR-1955-3p	MIMAT0017348	1.21144879	0.001112936
mmu-miR-8119	MIMAT0031425	1.192086412	0.000124581
mmu-miR-1927	MIMAT0009390	1.171642625	0.006992459
mmu-miR-7088-5p	MIMAT0028082	1.168726463	0.000102545
mmu-miR-7086-5p	MIMAT0028078	1.146093643	0.004518173
mmu-miR-327	MIMAT0004867	1.134257496	2.03E-05
mmu-miR-129b-5p	MIMAT0029862	1.12344253	0.008364393

mmu-miR-100-5p	MIMAT0000655	1.107692903	0.000717856
mmu-miR-3472	MIMAT0015643	1.105657762	0.033640307
mmu-miR-8100	MIMAT0031403	1.095705708	0.001110463
mmu-miR-7080-5p	MIMAT0028066	1.094982755	0.002529451
mmu-miR-497a-5p	MIMAT0003453	1.092852889	0.000240688
mmu-miR-7233-5p	MIMAT0028434	1.08885717	0.00773256
mmu-miR-10b-5p	MIMAT0000208	1.083959411	4.44E-05
mmu-miR-5113	MIMAT0020621	1.062412826	4.70E-05
mmu-miR-6975-5p	MIMAT0027852	1.061642028	0.00161935
mmu-miR-1931	MIMAT0009394	1.0615396	0.004252304
mmu-miR-5118	MIMAT0020626	1.058113201	0.001242108
mmu-miR-6980-5p	MIMAT0027862	1.022387012	0.004626791
mmu-miR-6418-5p	MIMAT0025173	1.016818378	3.05E-05
mmu-miR-7084-5p	MIMAT0028074	1.014630755	7.34E-06
mmu-miR-1957a	MIMAT0009430	1.011373007	0.001841627
mmu-miR-204-5p	MIMAT0000237	1.006287754	5.16E-05
mmu-miR-7216-5p	MIMAT0028400	0.992557116	0.001161442
mmu-miR-691	MIMAT0003470	0.988453848	0.000638653
mmu-miR-7055-5p	MIMAT0028014	0.987731394	0.011090955
mmu-miR-5130	MIMAT0020641	0.978627464	0.001052069
mmu-miR-6904-5p	MIMAT0027708	0.977915683	0.013361691
mmu-miR-7056-5p	MIMAT0028016	0.972302941	0.000209861
mmu-miR-6970-5p	MIMAT0027842	0.972166274	0.00020407
mmu-miR-302c-5p	MIMAT0003375	0.9647001	0.009460083
mmu-miR-6998-5p	MIMAT0027898	0.962772267	0.000243148
mmu-miR-7218-5p	MIMAT0028404	0.94768965	0.011439132
mmu-miR-6921-5p	MIMAT0027742	0.944499055	0.000122687
mmu-miR-6918-5p	MIMAT0027736	0.943693575	0.013974815
mmu-miR-3075-5p	MIMAT0014858	0.943349179	0.001169575
mmu-miR-698-5p	MIMAT0022930	0.941516558	2.80E-06
mmu-miR-504-3p	MIMAT0017277	0.938898654	0.009992922
mmu-miR-378c	MIMAT0025138	0.934646549	0.001287879
mmu-miR-5104	MIMAT0020611	0.933548813	0.001234983
mmu-miR-24-3p	MIMAT0000219	0.912652045	0.000122687
mmu-miR-7019-5p	MIMAT0027942	0.909541879	1.32E-05
mmu-miR-29c-5p	MIMAT0004632	0.908673023	6.06E-05
mmu-miR-7028-5p	MIMAT0027960	0.905960567	0.000954724
mmu-miR-7234-3p	MIMAT0028437	0.89455736	0.003130627
mmu-miR-6946-5p	MIMAT0027792	0.893383911	0.000207147
mmu-miR-30b-5p	MIMAT0000130	0.890317838	0.004050699
mmu-miR-99a-5p	MIMAT0000131	0.879346164	0.004033596
mmu-miR-7035-5p	MIMAT0027974	0.878171514	0.000531154
mmu-let-7a-5p	MIMAT0000521	0.877075492	0.000111361
mmu-miR-6901-5p	MIMAT0027702	0.868165743	0.001086588
mmu-miR-6957-5p	MIMAT0027814	0.865397634	0.001030528
mmu-miR-6961-5p	MIMAT0027822	0.863279349	0.000185943
mmu-miR-190b-5p	MIMAT0004852	0.859407288	0.004516627
mmu-miR-3070-2-3p	MIMAT0014849	0.859212773	0.014216616
mmu-let-7g-5p	MIMAT0000121	0.857236179	0.000146678
mmu-miR-7012-5p	MIMAT0027928	0.841804589	9.12E-06

mmu-miR-30e-3p	MIMAT0000249	0.841103629	0.000472173
mmu-miR-7063-5p	MIMAT0028030	0.838608374	0.001242108
mmu-miR-7016-5p	MIMAT0027936	0.834315235	5.81E-05
mmu-miR-3059-3p	MIMAT0014812	0.826905081	9.25E-05
mmu-miR-378d	MIMAT0025167	0.818365202	0.029328854
mmu-miR-6923-5p	MIMAT0027746	0.814161947	9.86E-06
mmu-miR-194-5p	MIMAT0000224	0.811382315	0.000176888
mmu-miR-676-3p	MIMAT0003782	0.810552565	5.53E-05
mmu-miR-1968-5p	MIMAT0009441	0.80918555	3.02E-05
mmu-miR-1839-5p	MIMAT0009456	0.800307584	8.81E-05
mmu-miR-6401	MIMAT0025153	0.792933829	0.007666011
mmu-miR-7664-3p	MIMAT0029835	0.792070788	0.007156985
mmu-miR-7027-5p	MIMAT0027958	0.777455424	0.00047372
mmu-miR-7072-5p	MIMAT0028050	0.773024268	0.002002784
mmu-miR-29b-3p	MIMAT0000127	0.772298997	0.003033437
mmu-miR-16-5p	MIMAT0000527	0.772074909	0.001488909
mmu-miR-682	MIMAT0003459	0.771877539	0.005180874
mmu-miR-681	MIMAT0003458	0.76025331	0.002211128
mmu-miR-5107-5p	MIMAT0020615	0.759459211	0.000815732
mmu-miR-1930-3p	MIMAT0017340	0.755824424	0.000185282
mmu-miR-3085-5p	MIMAT0014878	0.753801562	0.000287384
mmu-miR-6973b-5p	MIMAT0027908	0.752426497	0.000207147
mmu-miR-7074-5p	MIMAT0028054	0.750669762	0.000517335
mmu-miR-1966-5p	MIMAT0009439	0.74620438	0.003412877
mmu-miR-378a-5p	MIMAT0000742	0.744196107	0.012827679
mmu-miR-7222-3p	MIMAT0028413	0.743858512	0.00012121
mmu-miR-1971	MIMAT0009446	0.742994813	0.001030528
mmu-miR-6905-5p	MIMAT0027710	0.742654424	0.040844176
mmu-miR-1967	MIMAT0009440	0.738843578	0.003404703
mmu-miR-30e-5p	MIMAT0000248	0.737443296	0.004185924
mmu-miR-143-3p	MIMAT0000247	0.734754483	0.002101332
mmu-miR-6922-5p	MIMAT0027744	0.728812126	0.000256761
mmu-miR-7070-5p	MIMAT0028046	0.728312019	0.004498021
mmu-miR-710	MIMAT0003500	0.724536068	0.000202028
mmu-miR-6971-5p	MIMAT0027844	0.723078828	0.000202028
mmu-miR-150-3p	MIMAT0004535	0.72213601	2.16E-05
mmu-let-7k	MIMAT0025580	0.720532083	2.46E-05
mmu-miR-8108	MIMAT0031413	0.718961314	0.000685952
mmu-miR-6911-5p	MIMAT0027722	0.718706526	8.73E-05
mmu-miR-7674-5p	MIMAT0029856	0.707707573	0.003855262
mmu-miR-711	MIMAT0003501	0.707008579	0.000521872
mmu-miR-6394	MIMAT0025144	0.70489944	0.001672458
mmu-miR-27b-3p	MIMAT0000126	0.701017905	0.037875595
mmu-miR-1897-5p	MIMAT0007864	0.699805239	0.011218456
mmu-miR-7075-5p	MIMAT0028056	0.699450021	0.000163154
mmu-miR-3110-3p	MIMAT0014952	0.696437662	0.001353663
mmu-miR-7045-5p	MIMAT0027994	0.685293355	0.000259805
mmu-miR-7682-3p	MIMAT0029885	0.684551463	0.034205796
mmu-miR-5110	MIMAT0020618	0.682207725	0.000747784
mmu-miR-6954-5p	MIMAT0027808	0.681015354	0.000321455

mmu-miR-30d-5p	MIMAT0000515	0.678377232	0.00535556
mmu-miR-192-5p	MIMAT0000517	0.674003939	0.000819531
mmu-miR-7668-3p	MIMAT0029843	0.673922522	0.002429574
mmu-miR-7034-5p	MIMAT0027972	0.659713126	0.000485684
mmu-miR-365-3p	MIMAT0000711	0.65143756	0.005512193
mmu-miR-208a-5p	MIMAT0017014	0.65027455	0.004983566
mmu-miR-6972-5p	MIMAT0027846	0.649827376	0.000332231
mmu-miR-193b-3p	MIMAT0004859	0.637621878	0.017142246
mmu-miR-145a-5p	MIMAT0000157	0.624360506	0.006236587
mmu-miR-6973a-5p	MIMAT0027848	0.618779974	0.000306304
mmu-miR-196b-5p	MIMAT0001081	0.616100927	0.044938961
mmu-miR-107-3p	MIMAT0000647	0.615580598	0.004983566
mmu-miR-181b-5p	MIMAT0000673	0.613480442	0.033848309
mmu-miR-1188-5p	MIMAT0005843	0.612692739	0.002370412
mmu-miR-7665-5p	MIMAT0029836	0.60894671	0.002101332
mmu-miR-341-3p	MIMAT0000588	0.607189217	0.00303544
mmu-miR-3099-3p	MIMAT0014816	0.604595147	0.009259217
mmu-miR-664-5p	MIMAT0017353	0.59246389	0.023439677
mmu-miR-3101-3p	MIMAT0014922	0.588673397	0.008363477
mmu-miR-1894-3p	MIMAT0007878	0.576134484	0.000485684
mmu-miR-8094	MIMAT0031395	0.561987417	0.002114628
mmu-miR-3095-3p	MIMAT0014912	0.555962642	0.009005669
mmu-miR-30c-2-3p	MIMAT0005438	0.552361731	0.000102431
mmu-miR-24-2-5p	MIMAT0005440	0.549884338	0.015495421
mmu-miR-3102-5p.2-5p	MIMAT0014934	0.548173165	0.02659469
mmu-miR-7011-5p	MIMAT0027926	0.548167584	0.009992922
mmu-miR-23a-5p	MIMAT0017019	0.541812267	0.001408586
mmu-miR-199b-5p	MIMAT0000672	0.539557235	0.021428983
mmu-miR-129-5p	MIMAT0000209	0.527010494	0.008434235
mmu-miR-151-5p	MIMAT0004536	0.526604069	0.031028962
mmu-miR-30b-3p	MIMAT0004524	0.525680063	0.001946971
mmu-let-7f-5p	MIMAT0000525	0.523959131	0.022982685
mmu-miR-22-3p	MIMAT0000531	0.523333887	0.011160185
mmu-miR-3072-5p	MIMAT0014852	0.520313402	0.002529451
mmu-miR-7032-5p	MIMAT0027968	0.520258753	0.008454003
mmu-miR-7007-5p	MIMAT0027918	0.518909421	0.005501759
mmu-miR-6386	MIMAT0025133	0.518680247	0.000899757
mmu-miR-7658-5p	MIMAT0029822	0.513920347	0.00122457
mmu-miR-6956-5p	MIMAT0027812	0.513367726	0.001447413
mmu-miR-218-5p	MIMAT0000663	0.512195342	0.009725379
mmu-miR-146a-5p	MIMAT0000158	0.487428171	0.037839433
mmu-miR-6984-5p	MIMAT0027870	0.486596413	0.023885584
mmu-miR-1895	MIMAT0007867	0.483129607	0.005314491
mmu-miR-669l-5p	MIMAT0009418	0.474444184	0.000819531
mmu-miR-3535	MIMAT0031410	0.469709174	0.001748998
mmu-miR-6370	MIMAT0025114	0.468205136	0.001050109
mmu-miR-195a-3p	MIMAT0017000	0.468198249	0.003475139
mmu-miR-7684-3p	MIMAT0029891	0.463157592	0.028198771
mmu-miR-6926-5p	MIMAT0027752	0.462676282	0.00176225
mmu-miR-1896	MIMAT0007873	0.455922688	0.005305751

mmu-miR-7211-3p	MIMAT0028391	0.450986206	0.025170476
mmu-miR-181d-5p	MIMAT0004324	0.445330121	0.008287112
mmu-miR-678	MIMAT0003452	0.442019771	0.005501759
mmu-miR-6349	MIMAT0025092	0.435753411	0.006115704
mmu-miR-10a-5p	MIMAT0000648	0.434484721	0.027003108
mmu-miR-7033-5p	MIMAT0027970	0.431290282	0.004641048
mmu-miR-103-3p	MIMAT0000546	0.427325654	0.019499258
mmu-miR-1892	MIMAT0007871	0.426628359	0.038289217
mmu-miR-7653-5p	MIMAT0029812	0.423941647	0.029328854
mmu-miR-139-5p	MIMAT0000656	0.420268419	0.022716151
mmu-miR-29b-1-5p	MIMAT0004523	0.398529322	0.022716151
mmu-miR-7043-5p	MIMAT0027990	0.394878655	0.010063361
mmu-miR-365-1-5p	MIMAT0017077	0.37791556	0.012262244
mmu-miR-6915-5p	MIMAT0027730	0.376105347	0.022620318
mmu-miR-6990-5p	MIMAT0027882	0.364975385	0.018773116
mmu-miR-8113	MIMAT0031419	0.334645755	0.025677805
mmu-miR-7069-5p	MIMAT0028044	0.329794819	0.011160185
mmu-miR-3067-3p	MIMAT0014841	0.32522775	0.01896059
mmu-miR-1934-3p	MIMAT0017341	0.323897721	0.028674533
mmu-miR-6982-5p	MIMAT0027866	0.229224012	0.045450587
mmu-miR-7672-5p	MIMAT0029850	-0.226570255	0.034575192
mmu-miR-149-3p	MIMAT0016990	-0.247983188	0.031184677
mmu-miR-6999-5p	MIMAT0027900	-0.305446552	0.018726285
mmu-miR-874-3p	MIMAT0004853	-0.315536332	0.005732844
mmu-miR-7002-5p	MIMAT0027906	-0.325425538	0.022708071
mmu-miR-7009-5p	MIMAT0027922	-0.32815693	0.043190008
mmu-miR-450a-2-3p	MIMAT0004789	-0.339964443	0.04245501
mmu-miR-3069-3p	MIMAT0014845	-0.359343123	0.025608624
mmu-miR-423-5p	MIMAT0004825	-0.364938396	0.020894942
mmu-miR-700-3p	MIMAT0003490	-0.369206107	0.00512983
mmu-miR-6931-5p	MIMAT0027762	-0.374369435	0.0304479
mmu-miR-5122	MIMAT0020630	-0.375229066	0.010310827
mmu-miR-470-5p	MIMAT0002111	-0.376927639	0.036642954
mmu-miR-877-5p	MIMAT0004861	-0.38892275	0.022231322
mmu-miR-6391	MIMAT0025140	-0.39262021	0.027003108
mmu-miR-155-5p	MIMAT0000165	-0.402348517	0.042051799
mmu-miR-1982-5p	MIMAT0009459	-0.413091701	0.002551325
mmu-miR-1906	MIMAT0007872	-0.416755556	0.00540298
mmu-miR-7018-5p	MIMAT0027940	-0.419005065	0.006032823
mmu-miR-99b-3p	MIMAT0004525	-0.420040443	0.001050109
mmu-miR-3058-3p	MIMAT0014814	-0.430050272	0.004718884
mmu-miR-8109	MIMAT0031415	-0.439393376	0.012598223
mmu-miR-92a-3p	MIMAT0000539	-0.454821204	0.020053312
mmu-miR-362-3p	MIMAT0004684	-0.455194667	0.019600917
mmu-miR-467a-3p	MIMAT0002108	-0.460600878	0.002481674
mmu-miR-7046-5p	MIMAT0027996	-0.461274061	0.012423809
mmu-miR-6937-5p	MIMAT0027774	-0.485596802	0.018726285
mmu-miR-6402	MIMAT0025154	-0.49235017	0.002211128
mmu-miR-350-3p	MIMAT0000605	-0.498294932	0.028191917
mmu-miR-6238	MIMAT0024859	-0.499763084	0.014685091

mmu-miR-532-5p	MIMAT0002889	-0.506687734	0.032728783
mmu-miR-6995-5p	MIMAT0027892	-0.507518204	0.005084776
mmu-miR-7050-5p	MIMAT0028004	-0.512581505	0.00118884
mmu-miR-5620-3p	MIMAT0022368	-0.514299522	0.00293707
mmu-miR-5131	MIMAT0020642	-0.523667493	0.000509515
mmu-miR-6952-5p	MIMAT0027804	-0.549485278	0.011160185
mmu-miR-6898-5p	MIMAT0027696	-0.553287101	0.021434778
mmu-miR-551b-5p	MIMAT0017236	-0.568073067	0.00059738
mmu-miR-28a-5p	MIMAT0000653	-0.56951245	0.005488036
mmu-miR-425-5p	MIMAT0004750	-0.584042472	0.005503692
mmu-miR-770-3p	MIMAT0003891	-0.585479584	0.004813546
mmu-miR-130b-3p	MIMAT0000387	-0.58573734	0.000966922
mmu-miR-1187	MIMAT0005837	-0.587228204	0.00540298
mmu-miR-5100	MIMAT0020607	-0.595623864	0.007798311
mmu-miR-3544-3p	MIMAT0022354	-0.598561172	0.010870532
mmu-miR-34a-5p	MIMAT0000542	-0.605418302	0.017319781
mmu-miR-211-3p	MIMAT0017059	-0.609146134	0.048751292
mmu-miR-6965-5p	MIMAT0027830	-0.613751062	0.000159989
mmu-miR-3102-3p	MIMAT0014936	-0.624548926	0.002349612
mmu-miR-295-5p	MIMAT0004575	-0.625429802	0.004370133
mmu-miR-6987-5p	MIMAT0027876	-0.628509085	0.000147745
mmu-miR-20b-5p	MIMAT0003187	-0.629357819	0.012987723
mmu-miR-7224-3p	MIMAT0028417	-0.632640998	0.000728815
mmu-miR-1249-3p	MIMAT0010560	-0.637269287	5.53E-05
mmu-miR-93-5p	MIMAT0000540	-0.643420402	0.015453138
mmu-miR-130a-3p	MIMAT0000141	-0.672341922	0.004983566
mmu-miR-532-3p	MIMAT0004781	-0.67410476	0.002765672
mmu-miR-125a-3p	MIMAT0004528	-0.689627793	0.003404703
mmu-miR-3058-5p	MIMAT0014813	-0.723964476	0.000133685
mmu-miR-17-3p	MIMAT0000650	-0.724284034	0.001014579
mmu-miR-6538	MIMAT0025583	-0.72443005	0.000569696
mmu-miR-296-5p	MIMAT0000374	-0.726177518	3.51E-05
mmu-miR-6360	MIMAT0025103	-0.733997771	0.004033596
mmu-miR-7115-3p	MIMAT0028128	-0.737438716	0.00033949
mmu-miR-483-3p	MIMAT0003120	-0.74137059	0.016772267
mmu-miR-152-3p	MIMAT0000162	-0.742656668	0.026660806
mmu-miR-139-3p	MIMAT0004662	-0.749676782	0.00044691
mmu-miR-7241-3p	MIMAT0028451	-0.758340029	2.45E-05
mmu-miR-6912-5p	MIMAT0027724	-0.76072933	0.000110563
mmu-miR-3102-5p	MIMAT0014933	-0.767489345	1.36E-05
mmu-miR-712-5p	MIMAT0003502	-0.770342396	0.000270456
mmu-miR-878-3p	MIMAT0004933	-0.776739816	0.000113245
mmu-miR-674-3p	MIMAT0003741	-0.784429994	0.001425877
mmu-miR-6997-5p	MIMAT0027896	-0.796575049	0.000890397
mmu-miR-615-3p	MIMAT0003783	-0.804811576	0.001445471
mmu-miR-3085-3p	MIMAT0014879	-0.834713875	0.000126335
mmu-miR-99b-5p	MIMAT0000132	-0.852094442	0.004626791
mmu-miR-6378	MIMAT0025124	-0.853689573	0.021121841
mmu-miR-6969-5p	MIMAT0027840	-0.854159188	0.004396846
mmu-miR-290a-5p	MIMAT0000366	-0.85523533	5.30E-05

mmu-miR-8101	MIMAT0031405	-0.87130063	0.000485684
mmu-miR-338-5p	MIMAT0004647	-0.875168361	0.000995653
mmu-miR-191-3p	MIMAT0004542	-0.881532773	0.003855262
mmu-miR-19a-3p	MIMAT0000651	-0.894615342	0.002143768
mmu-miR-7226-5p	MIMAT0028420	-0.8971275	2.03E-05
mmu-miR-7058-5p	MIMAT0028020	-0.898641072	0.004916263
mmu-miR-7a-5p	MIMAT0000677	-0.904499764	8.08E-05
mmu-miR-6976-3p	MIMAT0027855	-0.927675854	1.10E-05
mmu-miR-3620-3p	MIMAT0029879	-0.929128316	0.0002258
mmu-miR-702-3p	MIMAT0003492	-0.947817804	0.000118128
mmu-miR-7036a-5p	MIMAT0027976	-0.953615722	0.000404164
mmu-miR-7666-3p	MIMAT0029839	-0.96009289	5.71E-06
mmu-miR-224-5p	MIMAT0000671	-0.962975064	0.004789388
mmu-miR-6368	MIMAT0025112	-0.965145547	1.75E-05
mmu-miR-362-5p	MIMAT0000706	-0.96559231	0.003659803
mmu-miR-188-5p	MIMAT0000217	-0.969428992	2.09E-05
mmu-miR-15b-5p	MIMAT0000124	-0.970543055	0.000262539
mmu-miR-135a-1-3p	MIMAT0004531	-0.972813032	0.00535556
mmu-miR-697	MIMAT0003487	-0.983237017	2.54E-05
mmu-miR-20a-5p	MIMAT0000529	-0.998063958	0.000634149
mmu-miR-7666-5p	MIMAT0029838	-1.010184542	5.81E-05
mmu-miR-455-3p	MIMAT0003742	-1.014514465	0.015235403
mmu-miR-3154	MIMAT0035714	-1.032069757	5.83E-05
mmu-miR-5099	MIMAT0020606	-1.033978982	0.0006152
mmu-miR-6769b-5p	MIMAT0028040	-1.049460047	0.000408667
mmu-miR-19b-3p	MIMAT0000513	-1.055708632	0.000847966
mmu-miR-3081-5p	MIMAT0014870	-1.059869284	9.59E-06
mmu-miR-7022-3p	MIMAT0027949	-1.067394817	0.000112912
mmu-miR-7686-5p	MIMAT0029898	-1.074614267	0.000237234
mmu-miR-8105	MIMAT0031409	-1.076785134	3.19E-05
mmu-miR-8117	MIMAT0031423	-1.096511827	0.015558409
mmu-miR-199a-5p	MIMAT0000229	-1.098804563	0.009221073
mmu-miR-677-3p	MIMAT0017246	-1.099864328	7.72E-05
mmu-miR-7042-5p	MIMAT0027988	-1.101424648	0.006277343
mmu-miR-6908-5p	MIMAT0027716	-1.110712162	3.30E-06
mmu-miR-7648-3p	MIMAT0029799	-1.119103218	5.30E-05
mmu-miR-7684-5p	MIMAT0029890	-1.137804974	5.53E-05
mmu-miR-2137	MIMAT0011213	-1.137890004	2.34E-06
mmu-miR-6984-3p	MIMAT0027871	-1.150877679	0.003033437
mmu-miR-18a-5p	MIMAT0000528	-1.160187222	0.000147536
mmu-miR-3620-5p	MIMAT0029878	-1.170735368	5.92E-06
mmu-miR-6366	MIMAT0025110	-1.187467961	0.000223614
mmu-miR-154-5p	MIMAT0000164	-1.190143101	0.001933512
mmu-miR-3092-3p	MIMAT0014906	-1.196321323	5.81E-05
mmu-miR-376c-3p	MIMAT0003183	-1.203213912	0.000202028
mmu-miR-2861	MIMAT0013803	-1.209398966	6.64E-07
mmu-miR-434-3p	MIMAT0001422	-1.236811622	0.00053277
mmu-miR-494-3p	MIMAT0003182	-1.249358352	0.014339896
mmu-miR-3473g	MIMAT0031427	-1.251684163	6.64E-07
mmu-miR-7671-3p	MIMAT0029849	-1.255461067	2.81E-06

mmu-miR-7047-5p	MIMAT0027998	-1.257354429	0.000992479
mmu-miR-370-3p	MIMAT0001095	-1.268967753	0.000170776
mmu-miR-382-5p	MIMAT0000747	-1.277459884	0.000202028
mmu-miR-210-3p	MIMAT0000658	-1.313154084	0.009711254
mmu-miR-212-3p	MIMAT0000659	-1.318503301	0.000123999
mmu-miR-6412	MIMAT0025165	-1.367950584	0.001165658
mmu-miR-680	MIMAT0003457	-1.373146884	1.33E-05
mmu-miR-34b-5p	MIMAT0000382	-1.394335478	5.53E-05
mmu-miR-6963-5p	MIMAT0027826	-1.39512704	9.84E-05
mmu-miR-329-3p	MIMAT0000567	-1.412745682	8.81E-05
mmu-miR-202-3p	MIMAT0000235	-1.430292264	2.87E-06
mmu-miR-214-5p	MIMAT0004664	-1.430592227	0.0013761
mmu-miR-31-5p	MIMAT0000538	-1.441893222	0.000296587
mmu-miR-652-5p	MIMAT0017260	-1.496431935	0.000669502
mmu-miR-299a-3p	MIMAT0004577	-1.572063628	6.95E-05
mmu-miR-322-5p	MIMAT0000548	-1.592640875	6.06E-05
mmu-miR-1897-3p	MIMAT0007865	-1.599705627	5.62E-06
mmu-miR-125a-5p	MIMAT0000135	-1.72747777	4.32E-05
mmu-miR-431-5p	MIMAT0001418	-1.728355871	1.97E-05
mmu-miR-487b-3p	MIMAT0003184	-1.752123036	0.000257426
mmu-miR-337-5p	MIMAT0004644	-1.77393784	1.33E-05
mmu-miR-434-5p	MIMAT0001421	-1.806312568	1.13E-05
mmu-miR-34c-5p	MIMAT0000381	-1.831968839	4.76E-05
mmu-miR-411-5p	MIMAT0004747	-1.835300843	4.44E-05
mmu-miR-1949	MIMAT0009416	-1.846092538	2.76E-06
mmu-miR-21a-5p	MIMAT0000530	-1.868722061	9.50E-06
mmu-miR-495-3p	MIMAT0003456	-1.89673976	3.93E-06
mmu-miR-337-3p	MIMAT0000578	-1.901052359	6.69E-06
mmu-miR-381-3p	MIMAT0000746	-1.981901826	3.20E-07
mmu-miR-377-3p	MIMAT0000741	-1.982753763	1.81E-06
mmu-miR-299b-5p	MIMAT0022836	-2.002996103	2.34E-05
mmu-miR-411-3p	MIMAT0001093	-2.010254169	2.84E-06
mmu-miR-154-3p	MIMAT0004537	-2.023868262	9.01E-07
mmu-miR-376a-3p	MIMAT0000740	-2.112328809	3.45E-06
mmu-miR-409-3p	MIMAT0001090	-2.117896798	1.97E-07
mmu-miR-214-3p	MIMAT0000661	-2.194747489	2.54E-05
mmu-miR-3968	MIMAT0019352	-2.429731492	0.000329144
mmu-miR-127-3p	MIMAT0000139	-2.438877674	2.03E-05
mmu-miR-21a-3p	MIMAT0004628	-2.440079513	2.78E-07
mmu-miR-410-3p	MIMAT0001091	-2.442405098	4.53E-07
mmu-miR-300-3p	MIMAT0000378	-2.511026255	2.59E-07
mmu-miR-379-5p	MIMAT0000743	-2.708450239	3.80E-06
mmu-miR-503-5p	MIMAT0003188	-2.8506012	2.84E-06
mmu-miR-541-5p	MIMAT0003170	-2.927417376	1.97E-07
mmu-miR-376b-3p	MIMAT0001092	-3.192497413	2.59E-07
mmu-miR-335-5p	MIMAT0000766	-3.425965894	5.62E-06
mmu-miR-206-3p	MIMAT0000239	-4.426338251	0.00011953

Supplementary Table 5. Genes dysregulated in 6 weeks SYN compared to 1 week SYN samples (adj.p.val < 0.05)

Agilent Probe ID	Gene	Average Expression	Log_2FC	adj.p.val	Description
**Up-Regulated Genes**					
A_52_P534583	Ahsp	13.63	10.10	1.30E-06	ref|Mus musculus alpha hemoglobin stabilizing protein (Ahsp), mRNA [NM_133245]
A_55_P2714921	Ctse	10.99	9.47	1.30E-07	ref|Mus musculus cathepsin E (Ctse), mRNA [NM_007799]
A_55_P2732296	Hemgn	9.72	9.46	1.89E-07	ref|Mus musculus hemogen (Hemgn), mRNA [NM_053149]
A_51_P299062	Kel	10.47	9.15	1.24E-07	ref|Mus musculus Kell blood group (Kel), mRNA [NM_032540]
A_51_P417891	Trim10	10.12	9.14	1.21E-07	ref|Mus musculus tripartite motif-containing 10 (Trim10), mRNA [NM_011280]
A_55_P2045931	Btnl10	9.02	9.03	5.44E-08	ref|Mus musculus butyrophilin-like 10 (Btnl10), mRNA [NM_138678]
A_55_P2011678	Pdzk1ip1	11.53	9.02	1.90E-07	ref|Mus musculus PDZK1 interacting protein 1 (Pdzk1ip1), transcript variant 1, mRNA [NM_001164557]
A_66_P118929	Kel	9.23	9.02	3.16E-07	ref|Mus musculus Kell blood group (Kel), mRNA [NM_032540]
A_51_P469568	Cldn13	10.04	9.02	5.15E-07	ref|Mus musculus claudin 13 (Cldn13), mRNA [NM_020504]
A_55_P2046852	Gata1	10.74	8.86	3.42E-08	ref|Mus musculus GATA binding protein 1 (Gata1), mRNA [NM_008089]
A_55_P2099952	Car1	9.49	8.82	1.63E-08	ref|Mus musculus carbonic anhydrase 1 (Car1), transcript variant 1, mRNA [NM_009799]
A_51_P327451	Alas2	13.08	8.74	7.17E-09	ref|Mus musculus aminolevulinic acid synthase 2, erythroid (Alas2), transcript variant 1, mRNA [NM_009653]
A_51_P509679	Igha	14.12	8.72	1.38E-05	ens|immunoglobulin heavy constant alpha [Source:MGI Symbol;Acc:MGI:96444] [ENSMUST00000178282]
A_51_P509643	Snca	11.86	8.69	2.62E-08	ref|Mus musculus synuclein, alpha (Snca), transcript variant 1, mRNA [NM_001042451]
A_51_P241769	Rhd	12.40	8.68	7.15E-08	ref|Mus musculus Rh blood group, D antigen (Rhd), mRNA [NM_011270]
A_51_P150710	Jchain	11.32	8.67	1.69E-08	ref|Mus musculus immunoglobulin joining chain (Jchain), mRNA [NM_152839]
A_55_P2108151	Hbb-b1	13.52	8.52	2.60E-07	ref|Mus musculus hemoglobin, beta adult major chain (Hbb-b1), mRNA [NM_001278161]
A_51_P386625	Epx	9.01	8.51	0.0002444	ref|Mus musculus eosinophil peroxidase (Epx), mRNA [NM_007946]
A_55_P2038540	Hbb-b2	15.08	8.49	1.41E-07	ref|Mus musculus hemoglobin, beta adult minor chain (Hbb-b2), mRNA [NM_016956]
A_55_P2136906	Vpreb3	11.95	8.49	7.73E-07	ref|Mus musculus pre-B lymphocyte gene 3 (Vpreb3), mRNA [NM_009514]
**Down-Regulated Genes**					
A_55_P2090025	Mest	8.62	-11.58	1.57E-12	ref|Mus musculus mesoderm specific transcript (Mest), transcript variant 1, mRNA [NM_001252292]
A_55_P2066440	Myh3	6.10	-11.27	1.18E-09	ref|Mus musculus myosin, heavy polypeptide 3, skeletal muscle, embryonic (Myh3), mRNA [NM_001099635]
A_55_P2151868	Mest	6.55	-9.94	5.86E-13	ref|Mus musculus mesoderm specific transcript (Mest), transcript variant 1, mRNA [NM_001252292]
A_66_P106131	Saa3	9.16	-9.37	8.66E-08	ref|Mus musculus serum amyloid A 3 (Saa3), mRNA [NM_011315]
A_51_P288009	Chrng	5.42	-8.95	1.83E-11	ref|Mus musculus cholinergic receptor, nicotinic, gamma polypeptide (Chrng), mRNA [NM_009604]
A_66_P114804	Gm7325	5.32	-8.81	3.42E-08	ref|Mus musculus predicted gene 7325 (Gm7325), transcript variant 3, mRNA [NM_001177470]
A_30_P01026972	H19	8.59	-8.79	2.55E-11	lincRNA:chr7:149761435-149764019 reverse strand
A_55_P2744654	Myh8	4.91	-8.77	3.77E-11	ref|Mus musculus myosin, heavy polypeptide 8, skeletal muscle, perinatal (Myh8), mRNA [NM_177369]
A_55_P2903297	H19	11.45	-8.54	1.42E-10	ref|Mus musculus H19, imprinted maternally expressed transcript (H19), transcript variant 1, long non-coding RNA [NR_130973]
A_55_P2110548	Chrnd	5.36	-8.43	3.19E-10	ref|Mus musculus cholinergic receptor, nicotinic, delta polypeptide (Chrnd), mRNA [NM_021600]
A_55_P1990032	Cxcl5	8.15	-8.34	1.57E-12	ref|Mus musculus chemokine (C-X-C motif) ligand 5 (Cxcl5), mRNA [NM_009141]
A_55_P2076219	Prokr2	4.67	-8.17	3.76E-11	ref|Mus musculus prokineticin receptor 2 (Prokr2), mRNA [NM_144944]
A_51_P338262	Tnnt2	6.98	-7.79	5.01E-09	ref|Mus musculus troponin T2, cardiac (Tnnt2), transcript variant 9, mRNA [NM_011619]
A_55_P1971009	Gzme	5.14	-7.49	1.57E-11	ref|Mus musculus granzyme E (Gzme), mRNA [NM_010373]
A_51_P339793	Il1rl1	7.85	-7.49	7.59E-10	ref|Mus musculus interleukin 1 receptor-like 1 (Il1rl1), transcript variant 2, mRNA [NM_010743]
A_52_P588633	C1qtnf3	7.36	-7.19	4.64E-07	ref|Mus musculus C1q and tumor necrosis factor related protein 3 (C1qtnf3), transcript variant 2, mRNA [NM_030888]
A_55_P2619020	Postn	11.16	-7.19	2.60E-09	ref|Mus musculus periostin, osteoblast specific factor (Postn), transcript variant 3, mRNA [NM_001198766]
A_55_P2716356	Postn	7.36	-7.18	5.32E-10	ens|periostin, osteoblast specific factor [Source:MGI Symbol;Acc:MGI:1926321] [ENSMUST00000145036]
A_51_P503883	Plekhg4	7.78	-7.14	5.44E-08	ref|Mus musculus pleckstrin homology domain containing, family G (with RhoGef domain) member 4 (Plekhg4), mRNA [NM_001081333]

Supplementary Table 6: > 2 fold mRNA dysregulation between 1 week post DMM and sham SYN samples

Agilent Probe ID	Gene	Log_2FC	adj.p.val	Description
A_66_P115174	ENSMUST00000075226	3.173708291	0.999992814	ens|predicted pseudogene 10112 [Source:MGI Symbol;Acc:MGI:3800293] [ENSMUST00000075226]
A_66_P132787	ENSMUST00000103277	3.15537233	0.999992814	ens|T cell receptor beta, variable 20 [Source:MGI Symbol;Acc:MGI:98589] [ENSMUST00000103277]
A_52_P109740	Lpgat1	3.112443134	0.999992814	ens|lysophosphatidylglycerol acyltransferase 1 [Source:MGI Symbol;Acc:MGI:2446186] [ENSMUST00000138494]
A_55_P2042738	Tmem196	2.864414627	0.999992814	ref|Mus musculus transmembrane protein 196 (Tmem196), transcript variant 3, mRNA [NM_001294154
A_66_P130199	Slfn10-ps	2.792954394	0.999992814	ref|Mus musculus schlafen 10, pseudogene (Slfn10-ps), transcript variant 1, non-coding RNA [NR_073523
A_55_P2831539	Gm10612	2.545704724	0.999992814	gb|Mus musculus adult inner ear cDNA, RIKEN full-length enriched library, clone:F930118L16 product:hypothetical
A_30_P01031444	chr11:31647436-31647791_R	2.412546788	0.999992814	lincRNA:chr11:31647436-31647791 reverse strand
A_52_P274504	Dcaf12	2.26757553	0.999992814	gb|Mus musculus 2 days neonate thymus thymic cells cDNA, RIKEN full-length enriched library, clone:C920024D13
A_51_P405513	Zscan4d	2.164233621	0.999992814	ref|PREDICTED: Mus musculus zinc finger and SCAN domain containing 4D (Zscan4d), transcript variant X1, mRNA [
A_55_P2829085	Gm34799	2.161364728	0.999992814	ref|PREDICTED: Mus musculus predicted gene, 34799 (Gm34799), transcript variant X3, ncRNA [XR_385211
A_51_P491378	Csn1s1	2.095493986	0.999992814	ref|Mus musculus casein alpha s1 (Csn1s1), transcript variant 1, mRNA [NM_007784]
A_55_P2566166	Gm31137	1.958086413	0.999992814	ref|PREDICTED: Mus musculus predicted gene, 31137 (Gm31137), ncRNA [XR_373508]
A_55_P2608833	Med7	1.930118067	0.999992814	ref|Mus musculus mediator complex subunit 7 (Med7), transcript variant 1, mRNA [NM_025426]
A_55_P2166733	NAP059265-1	1.871769954	0.999992814	Unknown
A_55_P2603772	Hrasls	1.862044047	0.999992814	ens|HRAS-like suppressor [Source:MGI Symbol;Acc:MGI:1351473] [ENSMUST00000161294]
A_52_P420234	BG083441	1.816962553	0.999992814	gb|H3089B12-5 NIA Mouse 15K cDNA Clone Set Mus musculus cDNA clone H3089B12 5', mRNA sequence [BG0834
A_51_P271417	Fibcd1	1.790696363	0.999992814	ref|Mus musculus fibrinogen C domain containing 1 (Fibcd1), mRNA [NM_178887]
A_55_P2327518	1500032P08Rik	1.778780233	0.999992814	gb|Mus musculus adult male cerebellum cDNA, RIKEN full-length enriched library, clone:1500032P08 product:uncla
A_52_P223224	Hsd17b11	1.731452498	0.999992814	ens|hydroxysteroid (17-beta) dehydrogenase 11 [Source:MGI Symbol;Acc:MGI:2149821] [ENSMUST00000119025]
A_66_P125973	1700025N23Rik	1.71675533	0.999992814	ref|Mus musculus RIKEN cDNA 1700025N23 gene (1700025N23Rik), long non-coding RNA [NR_040523
A_55_P2143131	Rwdd3	1.714042283	0.999992814	ref|Mus musculus RWD domain containing 3 (Rwdd3), transcript variant 1, mRNA [NM_025637]
A_51_P145988	Vmn1r171	1.697943794	0.999992814	ref|Mus musculus vomeronasal 1 receptor 171 (Vmn1r171), mRNA [NM_030737]
A_30_P01027225	chr14:121850951-121854978_R	1.685179571	0.999992814	lincRNA:chr14:121850951-121854978 reverse strand
A_66_P125047	NAP059186-1	1.664791609	0.999992814	Unknown
A_66_P139022	ENSMUST00000117284	1.644555742	0.999992814	ens|predicted gene 649 [Source:MGI Symbol;Acc:MGI:2685495] [ENSMUST00000117284]
A_55_P2044182	Gm572	1.636067517	0.999992814	ref|Mus musculus predicted gene 572 (Gm572), mRNA [NM_001085505]
A_30_P01022022	chr1:59775022-59780348_R	1.621469565	0.999992814	lincRNA:chr1:59775022-59780348 reverse strand
A_55_P2414707	4732463B04Rik	1.621043168	0.999992814	gb|Mus musculus 10 days neonate skin cDNA, RIKEN full-length enriched library, clone:4732463B04 product:hypotl
A_55_P2075909	Vmn1r12	1.610371323	0.999992814	ref|Mus musculus vomeronasal 1 receptor 12 (Vmn1r12), mRNA [NM_001101579]
A_30_P01017835	chr7:6210340-6229115_F	1.602894223	0.999992814	lincRNA:chr7:6210340-6229115 forward strand
A_51_P110289	Lingo2	1.596414634	0.999992814	ref|Mus musculus leucine rich repeat and Ig domain containing 2 (Lingo2), transcript variant 3, mRNA [NM_175516
A_52_P562624	Pkhd1l1	1.594314152	0.999992814	ref|Mus musculus polycystic kidney and hepatic disease 1-like 1 (Pkhd1l1), mRNA [NM_138674]
A_30_P01017833	chr12:119836655-119984828_R	1.582223923	0.999992814	lincRNA:chr12:119836655-119984828 reverse strand
A_55_P2171258	Gm13180	1.579219467	0.999992814	gb|Mus musculus 2 cells egg cDNA, RIKEN full-length enriched library, clone:B020008H17 product:unclassifiable, fu
A_51_P415546	Defb6	1.57840927	0.999992814	ref|Mus musculus defensin beta 6 (Defb6), mRNA [NM_054074]
A_55_P2934693	1700019L22Rik	1.576684879	0.999992814	gb|Mus musculus adult male testis cDNA, RIKEN full-length enriched library, clone:1700019L22 product:unclassifial
A_66_P118547	BC027087	1.575752935	0.999992814	gb|Mus musculus cDNA clone IMAGE:5361338. [BC027087]
A_51_P215275	Hmx2	1.565972345	0.999992814	ref|Mus musculus H6 homeobox 2 (Hmx2), mRNA [NM_145998]
A_55_P2118436	H2-T3	1.533633868	0.999992814	ref|PREDICTED: Mus musculus histocompatibility 2, T region locus 3 (H2-T3), transcript variant X3, mRNA [XM_006

A_52_P458000	AI467606	1.53006531	0.999992814	ref|Mus musculus expressed sequence AI467606 (AI467606), mRNA [NM_178901]
A_66_P120521	AK030177	1.527248698	0.999992814	gb|Mus musculus adult male testis cDNA, RIKEN full-length enriched library, clone:4933412A06 product:unclassifia
A_55_P2784026	CJ060512	1.511871686	0.999992814	gb|CJ060512 RIKEN full-length enriched mouse cDNA library, C57BL [CJ060512]
A_65_P04809	BU787352	1.50501001	0.999992814	gb|il58d04.y1 Kaestner ngn3 wt Mus musculus cDNA 5', mRNA sequence [BU787352]
A_55_P2112972	Gm6288	1.503921267	0.999992814	ref|PREDICTED: Mus musculus predicted gene 6288 (Gm6288), transcript variant X9, misc_RNA [XR_869622]
A_51_P148478	Olfr510	1.490367935	0.999992814	ref|Mus musculus olfactory receptor 510 (Olfr510), mRNA [NM_146311]
A_51_P369784	Ces1e	1.485651751	0.999992814	ref|Mus musculus carboxylesterase 1E (Ces1e), mRNA [NM_133660]
A_55_P2745318	Jpx	1.479602969	0.999992814	gb|Mus musculus domesticus clone CJ-0.2 expressed neighbor of Xist non-coding RNA (Enox), partial sequence, alte
A_66_P117786	Crxos	1.472360647	0.999992814	ref|Mus musculus cone-rod homeobox, opposite strand (Crxos), transcript variant 1, mRNA [NM_001033638]
A_55_P2133978	Slc38a8	1.467250482	0.999992814	ref|Mus musculus solute carrier family 38, member 8 (Slc38a8), mRNA [NM_001009950]
A_52_P437820	Gm17767	1.446496666	0.999992814	ref|PREDICTED: Mus musculus predicted gene, 17767 (Gm17767), transcript variant X1, ncRNA [XR_865419]
A_52_P502226	2810429I04Rik	1.431138424	0.999992814	ref|Mus musculus RIKEN cDNA 2810429I04 gene (2810429I04Rik), long non-coding RNA [NR_015522]
A_52_P332513	Gm9798	1.42782748	0.999992814	gb|Mus musculus tripartite motif protein 8, mRNA (cDNA clone IMAGE:3668270), partial cds. [BC024694]
A_55_P2037752	Dtna	1.416144434	0.999992814	gb|Mus musculus 18 days pregnant adult female placenta and extra embryonic tissue cDNA, RIKEN full-length enric
A_55_P2517519	Pdik1l	1.414736353	0.999992814	ref|Mus musculus PDLIM1 interacting kinase 1 like (Pdik1l), transcript variant 1, mRNA [NM_146156]
A_55_P2165984	Olfr8	1.405367161	0.999992814	ref|Mus musculus olfactory receptor 8 (Olfr8), mRNA [NM_207201]
A_51_P150242	Olfr1362	1.404988501	0.999992814	ref|Mus musculus olfactory receptor 1362 (Olfr1362), mRNA [NM_146744]
A_55_P2142928	Cadm2	1.396283196	0.999992814	ref|Mus musculus cell adhesion molecule 2 (Cadm2), transcript variant 1, mRNA [NM_178721]
A_51_P217616	Chst3	1.389049969	0.999992814	ref|Mus musculus carbohydrate (chondroitin 6/keratan) sulfotransferase 3 (Chst3), mRNA [NM_016803]
A_66_P129819	ENSMUST00000068836	1.382447555	0.999992814	ens|predicted gene 9967 [Source:MGI Symbol;Acc:MGI:3704300] [ENSMUST00000068836]
A_55_P2111875	Taf12	1.372037601	0.999992814	ens|TAF12 RNA polymerase II, TATA box binding protein (TBP)-associated factor [Source:MGI Symbol;Acc:MGI:1913
A_66_P110461	BC018473	1.356175095	0.999992814	ref|Mus musculus cDNA sequence BC018473 (BC018473), long non-coding RNA [NR_003364]
A_66_P132121	Gm33602	1.353060032	0.999992814	ref|PREDICTED: Mus musculus predicted gene, 33602 (Gm33602), transcript variant X5, ncRNA [XR_864657]
A_65_P03318	A_65_P03318	1.351545005	0.999992814	Unknown
A_66_P110829	Dok2	1.349711164	0.999992814	ref|PREDICTED: Mus musculus docking protein 2 (Dok2), transcript variant X2, mRNA [XM_011244944]
A_52_P1075840	AK043872	1.349112121	0.999992814	gb|Mus musculus 10 days neonate cortex cDNA, RIKEN full-length enriched library, clone:A830044L07 product:uncl
A_55_P1998204	AK131853	1.339534458	0.999992814	gb|Mus musculus 10 day old male pancreas cDNA, RIKEN full-length enriched library, clone:1810063E24 product:ur
A_55_P2537234	A_55_P2537234	1.339354847	0.999992814	Unknown
A_55_P2502456	a	1.337561552	0.999992814	ref|Mus musculus nonagouti (a), mRNA [NM_015770]
A_66_P126727	Gm20750	1.33241735	0.999992814	ref|Mus musculus predicted gene, 20750 (Gm20750), long non-coding RNA [NR_040555]
A_52_P1019763	Panct2	1.327187551	0.999992814	ref|PREDICTED: Mus musculus pluripotency-associated noncoding transcript 2 (Panct2), transcript variant X1, misc_
A_55_P2416837	1700084F23Rik	1.324601304	0.999992814	ref|Mus musculus RIKEN cDNA 1700084F23 gene (1700084F23Rik), long non-coding RNA [NR_045965]
A_51_P301603	AI854517	1.319504969	0.999992814	ref|Mus musculus expressed sequence AI854517 (AI854517), transcript variant 1, long non-coding RNA [NR_04031
A_66_P109244	ENSMUST00000117238	1.315717878	0.999992814	ens|predicted gene 13761 [Source:MGI Symbol;Acc:MGI:3651274] [ENSMUST00000117238]
A_51_P206197	Gtf2a1l	1.309748733	0.999992814	ref|Mus musculus general transcription factor IIA, 1-like (Gtf2a1l), mRNA [NM_023630]
A_66_P111015	9530003J23Rik	1.306483457	0.999992814	ens|RIKEN cDNA 9530003J23 gene [Source:MGI Symbol;Acc:MGI:1924647] [ENSMUST00000159193]
A_52_P1171783	AK046770	1.29876672	0.999992814	gb|Mus musculus 10 days neonate medulla oblongata cDNA, RIKEN full-length enriched library, clone:B830002A04
A_30_P01028571	chr2:174092104-174106704_R	1.296903336	0.999992814	lincRNA:chr2:174092104-174106704 reverse strand
A_55_P2125738	Tas2r119	1.296878604	0.999992814	ref|Mus musculus taste receptor, type 2, member 119 (Tas2r119), mRNA [NM_020503]
A_55_P2079291	Aars	1.294849013	0.999992814	ens|alanyl-tRNA synthetase [Source:MGI Symbol;Acc:MGI:2384560] [ENSMUST00000034441]
A_55_P2557836	Gm36270	1.291577026	0.999992814	ref|PREDICTED: Mus musculus predicted gene, 36270 (Gm36270), ncRNA [XR_395827]
A_51_P193682	1700012B09Rik	1.275779031	0.999992814	ref|Mus musculus RIKEN cDNA 1700012B09 gene (1700012B09Rik), mRNA [NM_029306]

Supplementary Table 7: > 2 fold mRNA dysregulation between 6 week post DMM and sham SYN samples

Agilent Probe ID	Gene	Log_2FC	adj.p.val	Description
A_55_P2811679	Cxcl1	-1.72344	0.068617	ref|Mus musculus chemokine (C-X-C motif) ligand 1 (Cxcl1), mRNA [NM_008176]
A_51_P436652	Ccl7	-1.48269	0.068617	ref|Mus musculus chemokine (C-C motif) ligand 7 (Ccl7), mRNA [NM_013654]
A_51_P461894	Tnnc1	3.214999	0.068617	ref|Mus musculus troponin C, cardiac/slow skeletal (Tnnc1), mRNA [NM_009393]
A_66_P138345	Myh7b	3.631662	0.113213	ref|Mus musculus myosin, heavy chain 7B, cardiac muscle, beta (Myh7b), mRNA [NM_001085378]
A_55_P1990032	Cxcl5	-1.74345	0.11915	ref|Mus musculus chemokine (C-X-C motif) ligand 5 (Cxcl5), mRNA [NM_009141]
A_55_P2090205	Tpm3	2.139829	0.11915	ref|Mus musculus tropomyosin 3, gamma (Tpm3), transcript variant Tpm3.12, mRNA [NM_001293748]
A_55_P2088385	Tnnt1	3.088846	0.11915	ref|Mus musculus troponin T1, skeletal, slow (Tnnt1), transcript variant 1, mRNA [NM_001277903]
A_51_P362627	Tnnt1	3.163231	0.125985	ref|Mus musculus troponin T1, skeletal, slow (Tnnt1), transcript variant 2, mRNA [NM_011618]
A_55_P2093232	Myh7	4.376031	0.128119	ref|Mus musculus myosin, heavy polypeptide 7, cardiac muscle, beta (Myh7), mRNA [NM_080728]
A_65_P07389	Myh7	2.501658	0.132833	ens|myosin, heavy polypeptide 7, cardiac muscle, beta [Source:MGI Symbol;Acc:MGI:2155600] [ENSMUST00000149852]
A_66_P111661	Myh13	2.95834	0.132833	ref|Mus musculus myosin, heavy polypeptide 13, skeletal muscle (Myh13), mRNA [NM_001081250]
A_51_P114462	Ccl17	-1.69668	0.139078	ref|Mus musculus chemokine (C-C motif) ligand 17 (Ccl17), mRNA [NM_011332]
A_52_P403266	Myoz2	1.532997	0.139078	ens|myozenin 2 [Source:MGI Symbol;Acc:MGI:1913063] [ENSMUST00000140231]
A_52_P657360	Tnni1	3.184222	0.163363	ref|Mus musculus troponin I, skeletal, slow 1 (Tnni1), transcript variant 1, mRNA [NM_021467]
A_66_P127420	Tnni1	2.045083	0.304534	ref|Mus musculus troponin I, skeletal, slow 1 (Tnni1), transcript variant 1, mRNA [NM_021467]
A_51_P286737	Ccl2	-1.5702	0.440538	ref|Mus musculus chemokine (C-C motif) ligand 2 (Ccl2), mRNA [NM_011333]
A_52_P413395	Sln	2.705541	0.440538	ref|Mus musculus sarcolipin (Sln), mRNA [NM_025540]
A_55_P2003103	1500012K07Rik	2.090931	0.582298	ref|Mus musculus RIKEN cDNA 1500012K07 gene (1500012K07Rik), transcript variant 2, long non-coding RNA [NR_045812]
A_51_P205385	Uox	-1.0756	0.621682	ref|Mus musculus urate oxidase (Uox), mRNA [NM_009474]
A_51_P462153	Tpm3	1.000632	0.775768	ref|Mus musculus tropomyosin 3, gamma (Tpm3), transcript variant Tpm3.13, mRNA [NM_022314]
A_66_P104309	Myl2	3.400735	0.800847	ref|Mus musculus myosin, light polypeptide 2, regulatory, cardiac, slow (Myl2), mRNA [NM_010861]
A_51_P451075	Atp2a2	1.414804	0.9596	ref|Mus musculus ATPase, Ca++ transporting, cardiac muscle, slow twitch 2 (Atp2a2), transcript variant 2, mRNA [NM_009722]
A_52_P84217	Ophn1	-2.73411	0.999947	ens|oligophrenin 1 [Source:MGI Symbol;Acc:MGI:2151070] [ENSMUST00000156917]
A_66_P106131	Saa3	-2.15221	0.999947	ref|Mus musculus serum amyloid A 3 (Saa3), mRNA [NM_011315]
A_55_P2733427	Kif15	-2.1489	0.999947	gb|Mus musculus 2 days pregnant adult female oviduct cDNA, RIKEN full-length enriched library, clone:E230029J11 product:kinesin superfamily protein KIF15, full insert sequence. [AK087635]
A_52_P232813	Cxcl3	-2.05722	0.999947	ref|Mus musculus chemokine (C-X-C motif) ligand 3 (Cxcl3), mRNA [NM_203320]
A_30_P01028210	chrX:120590872-120598071_R	-2.01872	0.999947	lincRNA:chrX:120590872-120598071 reverse strand
A_51_P303160	Arg1	-1.94668	0.999947	ref|Mus musculus arginase, liver (Arg1), mRNA [NM_007482]
A_66_P136035	Tmeff2	-1.82957	0.999947	gb|Mus musculus adult male testis cDNA, RIKEN full-length enriched library, clone:4932418H01 product:transmembrane protein with EGF-like and two follistatin-like domains 2, full insert sequence. [AK030053]
A_30_P01028206	chr13:104802772-104802960_R	-1.8168	0.999947	lincRNA:chr13:104802772-104802960 reverse strand
A_52_P568200	BC057602	-1.75467	0.999947	gb|Mus musculus cDNA clone MGC:67258 IMAGE:6413648, complete cds. [BC057602]
A_66_P120406	A430072P03Rik	-1.7299	0.999947	ref|PREDICTED: Mus musculus RIKEN cDNA A430072P03 gene (A430072P03Rik), misc_RNA [XR_880530]
A_30_P01032987	chr13:66381200-66410200_R	-1.72021	0.999947	lincRNA:chr13:66381200-66410200 reverse strand
A_52_P167317	Nol4	-1.69887	0.999947	ref|Mus musculus nucleolar protein 4 (Nol4), transcript variant 2, mRNA [NM_199024]
A_55_P2546692	Gm36212	-1.67465	0.999947	ref|PREDICTED: Mus musculus predicted gene, 36212 (Gm36212), ncRNA [XR_862659]
A_30_P01023350	chr8:80581177-80621652_F	-1.59645	0.999947	lincRNA:chr8:80581177-80621652 forward strand
A_66_P109624	NAP025355-001	-1.55703	0.999947	Unknown
A_30_P01031086	chr5:113771087-113771170_F	-1.54612	0.999947	lincRNA:chr5:113771087-113771170 forward strand
A_55_P1969575	Gm10701	-1.53204	0.999947	gb|Mus musculus 16 days neonate male medulla oblongata cDNA, RIKEN full-length enriched library, clone:G630005L12 product:hypothetical protein, full insert sequence. [AK144276]
A_55_P2722512	ENSMUST00000189047	-1.50564	0.999947	ens|predicted gene 28956 [Source:MGI Symbol;Acc:MGI:5579662] [ENSMUST00000189047]
A_30_P01028819	chr4:146345650-146356650_F	-1.49124	0.999947	lincRNA:chr4:146345650-146356650 forward strand
A_66_P115856	AK048337	-1.47607	0.999947	gb|Mus musculus 16 days embryo head cDNA, RIKEN full-length enriched library, clone:C130050C01 product:unclassifiable, full insert sequence. [AK048337]
A_52_P195640	AW551984	-1.46322	0.999947	ref|Mus musculus expressed sequence AW551984 (AW551984), transcript variant 2, mRNA [NM_178737]
A_55_P2729978	Gm36582	-1.46025	0.999947	ref|PREDICTED: Mus musculus predicted gene, 36582 (Gm36582), ncRNA [XR_378070]

A_66_P140611	AK080734	-1.45902	0.999947	gb|Mus musculus adult retina cDNA, RIKEN full-length enriched library, clone:A930026N12 product:unclassifiable, full insert sequence. [AK080734]
A_55_P2232410	AI195470	-1.45364	0.999947	gb|uc72a10.x1 Sugano mouse liver mlia Mus musculus cDNA clone IMAGE:1431162 3'. [AA985897]
A_51_P454422	Unc79	-1.45352	0.999947	ref|Mus musculus unc-79 homolog (C. elegans) (Unc79), mRNA [NM_001081017]
A_30_P01024156	chr12:76776232-76776905_F	-1.44588	0.999947	lincRNA:chr12:76776232-76776905 forward strand
A_51_P475788	ENSMUST00000071920	-1.43743	0.999947	ens|killer cell lectin-like receptor subfamily C, member 2 [Source:MGI Symbol;Acc:MGI:1336162] [ENSMUST00000071920]
A_66_P120390	BB624079	-1.41801	0.999947	gb|BB624079 RIKEN full-length enriched, adult male eyeball Mus musculus cDNA clone 7530429N01 5', mRNA sequence [BB624079]
A_30_P01019580	chr7:88131123-88136898_F	-1.40994	0.999947	lincRNA:chr7:88131123-88136898 forward strand
A_55_P2069724	Csta1	-1.40753	0.999947	ref|Mus musculus cystatin A1 (Csta1), mRNA [NM_001033239]
A_52_P691200	AK047392	-1.40138	0.999947	gb|Mus musculus 10 days neonate cerebellum cDNA, RIKEN full-length enriched library, clone:B930055D05 product:unclassifiable, full insert sequence. [AK047392]
A_66_P134715	Otx2os1	-1.39882	0.999947	ref|Mus musculus orthodenticle homolog 2 opposite strand 1 (Otx2os1), long non-coding RNA [NR_029384]
A_30_P01025531	chr10:61486151-61488363_F	-1.36822	0.999947	lincRNA:chr10:61486151-61488363 forward strand
A_55_P2141686	Efcab9	-1.33664	0.999947	ens|EF-hand calcium binding domain 9 [Source:MGI Symbol;Acc:MGI:1916556] [ENSMUST00000109377]
A_55_P1967286	ENSMUST00000103370	-1.33656	0.999947	ens|immunoglobulin kappa variable 5-39 [Source:MGI Symbol;Acc:MGI:2686255] [ENSMUST00000103370]
A_52_P256492	Grip2	-1.33646	0.999947	ref|Mus musculus glutamate receptor interacting protein 2 (Grip2), mRNA [NM_001159507]
A_55_P1961938	Otop3	-1.33568	0.999947	ref|Mus musculus otopetrin 3 (Otop3), mRNA [NM_027132]
A_52_P42245	Klrb1a	-1.33193	0.999947	ref|Mus musculus killer cell lectin-like receptor subfamily B member 1A (Klrb1a), transcript variant 1, mRNA [NM_010737]
A_52_P95096	1700025L06Rik	-1.32636	0.999947	gb|Mus musculus adult male testis cDNA, RIKEN full-length enriched library, clone:4930449M09 product:unclassifiable, full insert sequence. [AK015435]
A_66_P109491	Gm36807	-1.32518	0.999947	ref|PREDICTED: Mus musculus predicted gene, 36807 (Gm36807), transcript variant X1, mRNA [XM_011251873]
A_55_P2089076	Msmp	-1.31473	0.999947	ref|Mus musculus microseminoprotein, prostate associated (Msmp), mRNA [NM_001099314]
A_51_P494315	Olfr1313	-1.29885	0.999947	ref|Mus musculus olfactory receptor 1313 (Olfr1313), mRNA [NM_207150]
A_55_P2232155	D930019F10Rik	-1.29334	0.999947	gb|Mus musculus 16 days embryo head cDNA, RIKEN full-length enriched library, clone:C130018B16 product:unclassifiable, full insert sequence. [AK047872]
A_51_P158686	Gm6377	-1.29194	0.999947	ref|Mus musculus predicted gene 6377 (Gm6377), mRNA [NM_001037917]
A_51_P477972	Olfr948	-1.27949	0.999947	ref|Mus musculus olfactory receptor 948 (Olfr948), mRNA [NM_001011756]
A_55_P2823758	Aatk	-1.26408	0.999947	ens|apoptosis-associated tyrosine kinase [Source:MGI Symbol;Acc:MGI:1197518] [ENSMUST00000134319]
A_51_P174215	Dhrs2	-1.24558	0.999947	ref|Mus musculus dehydrogenase/reductase member 2 (Dhrs2), mRNA [NM_027790]
A_52_P87900	Fam107a	-1.23763	0.999947	ref|Mus musculus family with sequence similarity 107, member A (Fam107a), mRNA [NM_183187]
A_55_P2721891	BC055324	-1.2374	0.999947	ens|cDNA sequence BC055324 [Source:MGI Symbol;Acc:MGI:3590554] [ENSMUST00000162949]
A_55_P1992849	Adrb3	-1.2349	0.999947	ref|Mus musculus adrenergic receptor, beta 3 (Adrb3), mRNA [NM_013462]
A_55_P2822812	Gm26838	-1.23349	0.999947	gb|Mus musculus 6 days neonate spleen cDNA, RIKEN full-length enriched library, clone:F430007H14 product:unclassifiable, full insert sequence. [AK143665]
A_55_P2003488	ENSMUST00000103265	-1.22753	0.999947	ens|T cell receptor beta, variable 10 [Source:MGI Symbol;Acc:MGI:98584] [ENSMUST00000103265]
A_66_P132128	Wdr49	-1.22109	0.999947	ref|PREDICTED: Mus musculus WD repeat domain 49 (Wdr49), transcript variant X2, mRNA [XM_006502498]
A_52_P244349	Dcx	-1.20265	0.999947	ref|Mus musculus doublecortin (Dcx), transcript variant 4, mRNA [NM_010025]
A_52_P548470	Shank2	-1.19658	0.999947	ref|Mus musculus SH3/ankyrin domain gene 2 (Shank2), transcript variant 2, mRNA [NM_001113373]
A_66_P113374	Thsd7a	-1.19404	0.999947	gb|Mus musculus 15 days embryo head cDNA, RIKEN full-length enriched library, clone:D930002I12 product:unclassifiable, full insert sequence. [AK086074]
A_55_P1964911	Gm30454	-1.19104	0.999947	ref|PREDICTED: Mus musculus predicted gene, 30454 (Gm30454), transcript variant X1, ncRNA [XR_386395]
A_30_P01020100	chr2:116802549-116803286_R	-1.17741	0.999947	lincRNA:chr2:116802549-116803286 reverse strand
A_30_P01031720	chr12:73789535-73799787_R	-1.1746	0.999947	lincRNA:chr12:73789535-73799787 reverse strand
A_55_P2931095	Zic4	-1.17346	0.999947	ref|Mus musculus zinc finger protein of the cerebellum 4 (Zic4), mRNA [NM_009576]
A_51_P257951	Retnla	-1.17006	0.999947	ref|Mus musculus resistin like alpha (Retnla), mRNA [NM_020509]
A_55_P1999301	Cyp2e1	-1.168	0.999947	ref|Mus musculus cytochrome P450, family 2, subfamily e, polypeptide 1 (Cyp2e1), mRNA [NM_021282]
A_30_P01025690	chr6:148162552-148176577_R	-1.16594	0.999947	lincRNA:chr6:148162552-148176577 reverse strand
A_55_P2726994	Lrrc75aos2	-1.16078	0.999947	gb|Mus musculus adult male testis cDNA, RIKEN full-length enriched library, clone:1700008B19 product:unclassifiable, full insert sequence. [AK005754]
A_52_P276614	1700015E13Rik	-1.1443	0.999947	ref|Mus musculus RIKEN cDNA 1700015E13 gene (1700015E13Rik), mRNA [NM_001039593]
A_51_P184949	F12	-1.12923	0.999947	ref|Mus musculus coagulation factor XII (Hageman factor) (F12), mRNA [NM_021489]
A_51_P412926	Krt27	-1.12739	0.999947	ref|Mus musculus keratin 27 (Krt27), mRNA [NM_010666]
A_52_P1027776	AK034495	-1.12664	0.999947	gb|Mus musculus adult male diencephalon cDNA, RIKEN full-length enriched library, clone:9330200D03 product:unclassifiable, full insert sequence. [AK034495]
A_55_P2408112	4930431P03Rik	-1.1243	0.999947	ref|Mus musculus RIKEN cDNA 4930431P03 gene (4930431P03Rik), long non-coding RNA [NR_045059]
A_30_P01032126	chr3:26975624-26988699_R	-1.11995	0.999947	lincRNA:chr3:26975624-26988699 reverse strand
A_51_P210835	Nebl	-1.11961	0.999947	ens|nebulette [Source:MGI Symbol;Acc:MGI:1921353] [ENSMUST00000145545]

A_30_P01033157	chr14:47978277-47985238_R	-1.11957	0.999947	lincRNA:chr14:47978277-47985238 reverse strand
A_51_P354003	Pou3f4	-1.11885	0.999947	ref|Mus musculus POU domain, class 3, transcription factor 4 (Pou3f4), mRNA [NM_008901]
A_66_P119754	Stx7	-1.11801	0.999947	gb|Mus musculus 16 days neonate heart cDNA, RIKEN full-length enriched library, clone:D830040L21 product:syntaxin 7, full insert sequence. [AK142742]
A_30_P01018507	chrX:120784762-120785166_R	-1.11299	0.999947	lincRNA:chrX:120784762-120785166 reverse strand
A_55_P2545102	Gm32378	-1.11084	0.999947	ref|PREDICTED: Mus musculus predicted gene, 32378 (Gm32378), ncRNA [XR_377778]
A_51_P227386	En1	-1.10598	0.999947	ref|Mus musculus engrailed 1 (En1), mRNA [NM_010133]
A_55_P2729458	Dock4	-1.1038	0.999947	gb|Mus musculus 13 days embryo male testis cDNA, RIKEN full-length enriched library, clone:6030495I01 product:hypothetical Src homology 3 (SH3) domain containing protein, full insert sequence. [AK031713]
A_55_P2020311	Skint2	-1.10177	0.999947	ens|selection and upkeep of intraepithelial T cells 2 [Source:MGI Symbol;Acc:MGI:3649629] [ENSMUST00000106559]
A_55_P2098485	AK132990	-1.08242	0.999947	gb|Mus musculus adult male testis cDNA, RIKEN full-length enriched library, clone:4930545G21 product:hypothetical protein, full insert sequence. [AK132990]
A_51_P123625	Irg1	-1.08237	0.999947	ref|Mus musculus immunoresponsive gene 1 (Irg1), mRNA [NM_008392]
A_66_P125185	Gm13749	-1.0785	0.999947	ref|Mus musculus predicted gene 13749 (Gm13749), long non-coding RNA [NR_027824]
A_55_P2050528	Olfr913	-1.07717	0.999947	ref|Mus musculus olfactory receptor 913 (Olfr913), mRNA [NM_001011523]
A_51_P178772	Ces1f	-1.07483	0.999947	ref|Mus musculus carboxylesterase 1F (Ces1f), mRNA [NM_144930]
A_30_P01033083	chr18:84747776-84748219_F	-1.06525	0.999947	lincRNA:chr18:84747776-84748219 forward strand
A_51_P171075	Csf2	-1.05999	0.999947	ref|Mus musculus colony stimulating factor 2 (granulocyte-macrophage) (Csf2), mRNA [NM_009969]
A_51_P339613	Lcn9	-1.05341	0.999947	ref|Mus musculus lipocalin 9 (Lcn9), mRNA [NM_029959]
A_55_P1998591	Fam43b	-1.05095	0.999947	ref|Mus musculus family with sequence similarity 43, member B (Fam43b), mRNA [NM_001081672]
A_30_P01025190	chr1:42759171-42771337_R	-1.04824	0.999947	lincRNA:chr1:42759171-42771337 reverse strand
A_51_P108659	Pon1	-1.04676	0.999947	ref|Mus musculus paraoxonase 1 (Pon1), mRNA [NM_011134]
A_55_P2151413	Gml	-1.04357	0.999947	ref|Mus musculus GPI anchored molecule like protein (Gml), mRNA [NM_001177524]
A_55_P2914090	Pramef25	-1.03721	0.999947	ref|Mus musculus PRAME family member 25 (Pramef25), mRNA [NM_001126315]
A_55_P2052465	NAP029844-1	-1.03704	0.999947	Unknown
A_30_P01031313	chr6:93570531-93599006_R	-1.03401	0.999947	lincRNA:chr6:93570531-93599006 reverse strand
A_52_P80702	4930503B20Rik	-1.02539	0.999947	ref|Mus musculus RIKEN cDNA 4930503B20 gene (4930503B20Rik), mRNA [NM_029144]
A_66_P101985	A630001O12Rik	-1.02453	0.999947	ref|PREDICTED: Mus musculus RIKEN cDNA A630001O12 gene (A630001O12Rik), misc_RNA [XR_863755]
A_66_P103516	Mest	-1.01923	0.999947	ens|mesoderm specific transcript [Source:MGI Symbol;Acc:MGI:96968] [ENSMUST00000131465]
A_51_P419226	S100a14	-1.01918	0.999947	ref|Mus musculus S100 calcium binding protein A14 (S100a14), transcript variant 2, mRNA [NM_025393]
A_55_P2482301	Gm7861	-1.01875	0.999947	ref|Mus musculus predicted gene 7861 (Gm7861), mRNA [NM_001177526]
A_55_P1965030	Slc5a12	-1.01865	0.999947	ref|Mus musculus solute carrier family 5 (sodium/glucose cotransporter), member 12 (Slc5a12), transcript variant 1, mRNA [NM_001003915]
A_66_P109628	AK081357	-1.01805	0.999947	gb|Mus musculus 16 days embryo head cDNA, RIKEN full-length enriched library, clone:C130009N01 product:unclassifiable, full insert sequence. [AK081357]
A_51_P283456	Cyp2e1	-1.01527	0.999947	ref|Mus musculus cytochrome P450, family 2, subfamily e, polypeptide 1 (Cyp2e1), mRNA [NM_021282]
A_55_P2935695	Mctp2	-1.01396	0.999947	gb|Mus musculus activated spleen cDNA, RIKEN full-length enriched library, clone:F830111H15 product:hypothetical protein, full insert sequence. [AK156983]
A_30_P01027954	chrX:34360500-34388500_F	-1.01279	0.999947	lincRNA:chrX:34360500-34388500 forward strand
A_51_P156955	Cfd	-1.01189	0.999947	ref|Mus musculus complement factor D (adipsin) (Cfd), transcript variant 1, mRNA [NM_013459]
A_52_P90363	Ifi27l2a	-1.01057	0.999947	ref|Mus musculus interferon, alpha-inducible protein 27 like 2A (Ifi27l2a), transcript variant 1, mRNA [NM_029803]
A_55_P2069256	Prr19	-1.00997	0.999947	ref|Mus musculus proline rich 19 (Prr19), mRNA [NM_001081294]
A_66_P115578	Gm32364	-1.00876	0.999947	ref|PREDICTED: Mus musculus predicted gene, 32364 (Gm32364), transcript variant X11, ncRNA [XR_871792]
A_52_P430322	NAP020145-001	-1.00662	0.999947	Unknown
A_66_P111464	Trpm1	-1.00558	0.999947	ref|Mus musculus transient receptor potential cation channel, subfamily M, member 1 (Trpm1), transcript variant 2, mRNA [NM_001039104]
A_55_P2929644	Aldh3a2	1.006647	0.999947	ref|Mus musculus aldehyde dehydrogenase family 3, subfamily A2 (Aldh3a2), mRNA [NM_007437]
A_55_P1977220	Ferd3l	1.00712	0.999947	ref|Mus musculus Fer3-like (Drosophila) (Ferd3l), mRNA [NM_033522]
A_55_P1974367	Fabp3	1.009057	0.999947	ref|Mus musculus fatty acid binding protein 3, muscle and heart (Fabp3), mRNA [NM_010174]
A_55_P2720878	Kbtbd12	1.0116	0.999947	ens|kelch repeat and BTB (POZ) domain containing 12 [Source:MGI Symbol;Acc:MGI:1918481] [ENSMUST00000184743]
A_55_P2173952	Myh6	1.016747	0.999947	ref|Mus musculus myosin, heavy polypeptide 6, cardiac muscle, alpha (Myh6), transcript variant 1, mRNA [NM_001164171]
A_52_P48218	Tdrd1	1.020889	0.999947	ref|Mus musculus tudor domain containing 1 (Tdrd1), transcript variant 3, mRNA [NM_001002241]
A_52_P205710	Myl10	1.025314	0.999947	ref|Mus musculus myosin, light chain 10, regulatory (Myl10), transcript variant 1, mRNA [NM_021611]
A_52_P159276	Grhl1	1.025908	0.999947	ref|Mus musculus grainyhead-like 1 (Drosophila) (Grhl1), transcript variant 2, mRNA [NM_145890]
A_55_P2197418	C80360	1.027197	0.999947	gb|C80360 Mouse 3.5-dpc blastocyst cDNA Mus musculus cDNA clone J0080C10 3', mRNA sequence [C80360]
A_55_P2211957	C330022C24Rik	1.027726	0.999947	ref|Mus musculus RIKEN cDNA C330022C24 gene (C330022C24Rik), long non-coding RNA [NR_045717]

A_55_P2075909	Vmn1r12	1.029116	0.999947	ref|Mus musculus vomeronasal 1 receptor 12 (Vmn1r12), mRNA [NM_001101579]
A_55_P2162825	Smco3	1.029479	0.999947	ref|Mus musculus single-pass membrane protein with coiled-coil domains 3 (Smco3), mRNA [NM_001039558]
A_52_P812362	BC064078	1.032142	0.999947	ref|Mus musculus cDNA sequence BC064078 (BC064078), long non-coding RNA [NR_015455]
A_52_P374960	Ostn	1.032789	0.999947	ref|Mus musculus osteocrin (Ostn), mRNA [NM_198112]
A_51_P253426	Olfr494	1.038589	0.999947	ref|Mus musculus olfactory receptor 494 (Olfr494), mRNA [NM_146737]
A_55_P2021689	Chrdl2	1.040105	0.999947	ref|Mus musculus chordin-like 2 (Chrdl2), transcript variant 2, mRNA [NM_133709]
A_65_P13435	Actn2	1.040765	0.999947	ref|Mus musculus actinin alpha 2 (Actn2), mRNA [NM_033268]
A_52_P747685	Gm33989	1.042302	0.999947	ref|PREDICTED: Mus musculus predicted gene, 33989 (Gm33989), transcript variant X2, mRNA [XM_011250924]
A_52_P818815	AK029633	1.04248	0.999947	gb|Mus musculus adult male testis cDNA, RIKEN full-length enriched library, clone:4930417J09 product:unclassifiable, full insert sequence. [AK029633]
A_55_P2424042	Ttn	1.04463	0.999947	ref|Mus musculus titin (Ttn), transcript variant N2-B, mRNA [NM_028004]
A_55_P2758927	Gm34979	1.044741	0.999947	ref|PREDICTED: Mus musculus predicted gene, 34979 (Gm34979), ncRNA [XR_877174]
A_55_P1993353	Slc44a5	1.045801	0.999947	ref|Mus musculus solute carrier family 44, member 5 (Slc44a5), mRNA [NM_001081263]
A_66_P116508	Gm13848	1.049226	0.999947	ref|PREDICTED: Mus musculus predicted gene 13848 (Gm13848), transcript variant X1, misc_RNA [XR_377513]
A_55_P2604167	Plxnc1	1.05321	0.999947	ens|plexin C1 [Source:MGI Symbol;Acc:MGI:1890127] [ENSMUST00000181244]
A_52_P642005	Camta1	1.053992	0.999947	ref|Mus musculus calmodulin binding transcription activator 1 (Camta1), transcript variant 1, mRNA [NM_001081557]
A_55_P2746505	Sox2	1.056264	0.999947	ref|Mus musculus SRY (sex determining region Y)-box 2 (Sox2), mRNA [NM_011443]
A_52_P464729	Hcrtr2	1.06041	0.999947	ens|hypocretin (orexin) receptor 2 [Source:MGI Symbol;Acc:MGI:2680765] [ENSMUST00000184757]
A_55_P2360271	9530056K15Rik	1.062403	0.999947	gb|Mus musculus 16 days neonate cerebellum cDNA, RIKEN full-length enriched library, clone:9630002I21 product:unclassifiable, full insert sequence. [AK035765]
A_65_P12037	BB207980	1.071186	0.999947	gb|BB207980 RIKEN full-length enriched, 0 day neonate thymus Mus musculus cDNA clone A430086A16 3'. [BB207980]
A_51_P235775	Mastl	1.078203	0.999947	ref|Mus musculus microtubule associated serine/threonine kinase-like (Mastl), mRNA [NM_025979]
A_51_P490337	Tmem190	1.080874	0.999947	ref|Mus musculus transmembrane protein 190 (Tmem190), mRNA [NM_030028]
A_51_P480311	F2	1.089904	0.999947	ref|Mus musculus coagulation factor II (F2), mRNA [NM_010168]
A_51_P295315	Ankrd2	1.09623	0.999947	ref|Mus musculus ankyrin repeat domain 2 (stretch responsive muscle) (Ankrd2), mRNA [NM_020033]
A_52_P411490	A830019P07Rik	1.097577	0.999947	ref|PREDICTED: Mus musculus RIKEN cDNA A830019P07 gene (A830019P07Rik), transcript variant X6, misc_RNA [XR_386670]
A_55_P2004547	Klra6	1.098951	0.999947	ref|Mus musculus killer cell lectin-like receptor, subfamily A, member 6 (Klra6), mRNA [NM_008464]
A_55_P2186282	Agxt2	1.100053	0.999947	ref|Mus musculus alanine-glyoxylate aminotransferase 2 (Agxt2), mRNA [NM_001031851]
A_55_P2909829	Frzb	1.10344	0.999947	ref|Mus musculus frizzled-related protein (Frzb), mRNA [NM_011356]
A_30_P01018969	chr4:53315503-53316050_F	1.10944	0.999947	lincRNA:chr4:53315503-53316050 forward strand
A_66_P138542	ENSMUST00000171792	1.109751	0.999947	ens|predicted gene 17198 [Source:MGI Symbol;Acc:MGI:4938025] [ENSMUST00000171792]
A_55_P2552388	Gm29832	1.110371	0.999947	ref|PREDICTED: Mus musculus predicted gene, 29832 (Gm29832), transcript variant X2, ncRNA [XR_380783]
A_66_P102708	Myh15	1.115888	0.999947	ref|Mus musculus myosin, heavy chain 15 (Myh15), mRNA [NM_001166210]
A_55_P2043699	Tmc1	1.120036	0.999947	gb|Mus musculus adult male testis cDNA, RIKEN full-length enriched library, clone:4933416G09 product:hypothetical protein, full insert sequence. [AK016832]
A_52_P175316	Rictor	1.123936	0.999947	gb|Mus musculus 0 day neonate cerebellum cDNA, RIKEN full-length enriched library, clone:C230066I17 product:hypothetical ARM repeat structure containing protein, full insert sequence. [AK082586]
A_55_P2724139	Mfsd7b	1.124777	0.999947	ref|PREDICTED: Mus musculus major facilitator superfamily domain containing 7B (Mfsd7b), transcript variant X2, mRNA [XM_006497152]
A_55_P2009239	4932414J04Rik	1.134386	0.999947	ref|Mus musculus RIKEN cDNA 4932414J04 gene (4932414J04Rik), long non-coding RNA [NR_028259]
A_55_P2006499	Esrrg	1.137982	0.999947	ref|Mus musculus estrogen-related receptor gamma (Esrrg), transcript variant 2, mRNA [NM_001243792]
A_52_P906899	1700101C01Rik	1.138869	0.999947	gb|Mus musculus adult male testis cDNA, RIKEN full-length enriched library, clone:1700101C01 product:unclassifiable, full insert sequence. [AK007104]
A_55_P2730761	E430016F16Rik	1.147031	0.999947	gb|Mus musculus 3 days neonate thymus cDNA, RIKEN full-length enriched library, clone:A630052G14 product:unclassifiable, full insert sequence. [AK042016]
A_30_P01023385	chr15:38639259-38639546_R	1.149897	0.999947	lincRNA:chr15:38639259-38639546 reverse strand
A_66_P112109	Gm4598	1.150908	0.999947	ref|Mus musculus predicted gene 4598 (Gm4598), long non-coding RNA [NR_030681]
A_55_P2007601	Sftpd	1.158935	0.999947	ref|Mus musculus surfactant associated protein D (Sftpd), mRNA [NM_009160]
A_51_P133012	Fbxo24	1.159221	0.999947	ref|Mus musculus F-box protein 24 (Fbxo24), mRNA [NM_027708]
A_52_P664783	Spef2	1.160733	0.999947	ref|Mus musculus sperm flagellar 2 (Spef2), transcript variant 3, mRNA [NM_001305044]
A_55_P2095380	1700012I11Rik	1.161085	0.999947	ref|Mus musculus RIKEN cDNA 1700012I11 gene (1700012I11Rik), long non-coding RNA [NR_045140]
A_55_P2004031	Ctrc	1.16817	0.999947	ref|Mus musculus chymotrypsin C (caldecrin) (Ctrc), mRNA [NM_001033875]
A_30_P01019872	chr8:128933150-128943375_F	1.168867	0.999947	lincRNA:chr8:128933150-128943375 forward strand
A_66_P121197	ENSMUST00000092369	1.170339	0.999947	ens|keratin associated protein 10-4 [Source:MGI Symbol;Acc:MGI:1925013] [ENSMUST00000092369]

A_66_P108886	AK051693	1.171987	0.999947	gb|Mus musculus 12 days embryo spinal ganglion cDNA, RIKEN full-length enriched library, clone:D130065P14 product:unclassifiable, full insert sequence. [AK051693]
A_55_P2017964	Unc13a	1.177215	0.999947	ref|Mus musculus unc-13 homolog A (C. elegans) (Unc13a), mRNA [NM_001029873]
A_66_P127235	4930426I24Rik	1.177771	0.999947	ref|PREDICTED: Mus musculus RIKEN cDNA 4930426I24 gene (4930426I24Rik), misc_RNA [XR_105498]
A_66_P100644	AK156094	1.180263	0.999947	gb|Mus musculus activated spleen cDNA, RIKEN full-length enriched library, clone:F830007M13 product:unclassifiable, full insert sequence. [AK156094]
A_55_P2735730	Gm34006	1.183468	0.999947	gb|Mus musculus mammary gland RCB-0527 Jyg-MC(B) cDNA, RIKEN full-length enriched library, clone:G930003B20 product:unclassifiable, full insert sequence. [AK145144]
A_30_P01024388	chrX:12811766-12813409_R	1.184781	0.999947	lincRNA:chrX:12811766-12813409 reverse strand
A_51_P108266	Actn2	1.186582	0.999947	ref|Mus musculus actinin alpha 2 (Actn2), mRNA [NM_033268]
A_52_P86635	D030068K23Rik	1.195573	0.999947	gb|Mus musculus 12 days embryo spinal ganglion cDNA, RIKEN full-length enriched library, clone:D130033M24 product:hypothetical protein, full insert sequence. [AK051321]
A_30_P01029845	chr3:41143423-41359623_R	1.196892	0.999947	lincRNA:chr3:41143423-41359623 reverse strand
A_55_P2810256	Casq2	1.201666	0.999947	ref|Mus musculus calsequestrin 2 (Casq2), mRNA [NM_009814]
A_55_P2025538	Ano4	1.203232	0.999947	ref|Mus musculus anoctamin 4 (Ano4), transcript variant 1, mRNA [NM_001277188]
A_52_P1194909	3110023J12Rik	1.20553	0.999947	gb|Mus musculus 13 days embryo head cDNA, RIKEN full-length enriched library, clone:3110023J12 product:unclassifiable, full insert sequence. [AK014075]
A_55_P2110548	Chrnd	1.206473	0.999947	ref|Mus musculus cholinergic receptor, nicotinic, delta polypeptide (Chrnd), mRNA [NM_021600]
A_52_P1115389	2310041K03Rik	1.20769	0.999947	gb|Mus musculus adult male tongue cDNA, RIKEN full-length enriched library, clone:2310041K03 product:unclassifiable, full insert sequence. [AK009740]
A_55_P2581670	Gm34360	1.211814	0.999947	ref|PREDICTED: Mus musculus predicted gene, 34360 (Gm34360), ncRNA [XR_383395]
A_55_P2148283	Gm20822	1.213046	0.999947	ref|Mus musculus predicted gene, 20822 (Gm20822), mRNA [NM_001199331]
A_55_P2550477	Gm33302	1.218148	0.999947	ref|PREDICTED: Mus musculus predicted gene, 33302 (Gm33302), transcript variant X1, ncRNA [XR_864251]
A_52_P361208	Kbtbd12	1.226175	0.999947	ref|Mus musculus kelch repeat and BTB (POZ) domain containing 12 (Kbtbd12), mRNA [NM_001278671]
A_30_P01027020	chr11:117835849-117842574_R	1.228588	0.999947	lincRNA:chr11:117835849-117842574 reverse strand
A_51_P193185	Mb	1.233277	0.999947	ref|Mus musculus myoglobin (Mb), transcript variant 2, mRNA [NM_013593]
A_65_P19877	BC050216	1.237121	0.999947	gb|Mus musculus cDNA clone IMAGE:30015155, partial cds. [BC050216]
A_51_P286748	Frzb	1.244519	0.999947	ref|Mus musculus frizzled-related protein (Frzb), mRNA [NM_011356]
A_30_P01018573	chr3:127170996-127201096_R	1.246259	0.999947	lincRNA:chr3:127170996-127201096 reverse strand
A_52_P370715	AK034179	1.249204	0.999947	gb|Mus musculus adult male diencephalon cDNA, RIKEN full-length enriched library, clone:9330161J18 product:unclassifiable, full insert sequence. [AK034179]
A_51_P435410	Grid2ip	1.258472	0.999947	ref|Mus musculus glutamate receptor, ionotropic, delta 2 (Grid2) interacting protein 1 (Grid2ip), transcript variant 2, mRNA [NM_133355]
A_55_P2034067	Myom3	1.258558	0.999947	ref|Mus musculus myomesin family, member 3 (Myom3), mRNA [NM_001085509]
A_52_P356084	Agmo	1.270915	0.999947	ens|alkylglycerol monooxygenase [Source:MGI Symbol;Acc:MGI:2442495] [ENSMUST00000160158]
A_30_P01022480	chr7:134595900-134610975_R	1.27368	0.999947	lincRNA:chr7:134595900-134610975 reverse strand
A_55_P2045871	ENSMUST00000176659	1.288366	0.999947	ens|vomeronasal 1 receptor, pseudogene 150 [Source:MGI Symbol;Acc:MGI:4439321] [ENSMUST00000176659]
A_51_P121915	BC089597	1.291632	0.999947	ref|Mus musculus cDNA sequence BC089597 (BC089597), mRNA [NM_145424]
A_55_P2288047	Igkv6-15	1.294583	0.999947	gb|AGENCOURT_10154517 NCI_CGAP_Co24 Mus musculus cDNA clone IMAGE:6529794 5', mRNA sequence [BU523154]
A_55_P2321889	4933404I11Rik	1.297308	0.999947	gb|Mus musculus adult male testis cDNA, RIKEN full-length enriched library, clone:4933404I11 product:unclassifiable, full insert sequence. [AK016650]
A_55_P2041728	Gm590	1.304687	0.999947	ref|Mus musculus predicted gene 590 (Gm590), mRNA [NM_001195437]
A_51_P157698	Oaz3	1.311633	0.999947	ref|Mus musculus ornithine decarboxylase antizyme 3 (Oaz3), mRNA [NM_016901]
A_52_P948388	BU961420	1.321254	0.999947	gb|AGENCOURT_10613907 NIH_MGC_169 Mus musculus cDNA clone IMAGE:6741956 5', mRNA sequence [BU961420]
A_51_P309804	Olfr584	1.334029	0.999947	ref|Mus musculus olfactory receptor 584 (Olfr584), mRNA [NM_147054]
A_55_P2742690	Cd226	1.336987	0.999947	gb|Mus musculus CD226 antigen, mRNA (cDNA clone IMAGE:3328279), complete cds. [BC051526]
A_55_P2120000	ENSMUST00000086672	1.337886	0.999947	ens|predicted gene 10192 [Source:MGI Symbol;Acc:MGI:3642867] [ENSMUST00000086672]
A_55_P2799081	Esrrg	1.340014	0.999947	ref|Mus musculus estrogen-related receptor gamma (Esrrg), transcript variant 2, mRNA [NM_001243792]
A_66_P119219	4930479D17Rik	1.379754	0.999947	ref|Mus musculus RIKEN cDNA 4930479D17 gene (4930479D17Rik), long non-coding RNA [NR_046277]
A_30_P01032142	chr5:119954412-119954855_R	1.391076	0.999947	lincRNA:chr5:119954412-119954855 reverse strand
A_55_P2744295	Triml1	1.391296	0.999947	ref|Mus musculus tripartite motif family-like 1 (Triml1), mRNA [NM_177742]
A_55_P2904073	Triml1	1.438818	0.999947	ref|Mus musculus tripartite motif family-like 1 (Triml1), mRNA [NM_177742]
A_55_P1953201	Olfr1301	1.450006	0.999947	ref|Mus musculus olfactory receptor 1301 (Olfr1301), mRNA [NM_146887]
A_51_P338262	Tnnt2	1.461031	0.999947	ref|Mus musculus troponin T2, cardiac (Tnnt2), transcript variant 9, mRNA [NM_011619]
A_51_P212630	Chd8	1.481906	0.999947	ref|Mus musculus chromodomain helicase DNA binding protein 8 (Chd8), mRNA [NM_201637]
A_30_P01032181	chr18:35967933-35986564_R	1.517272	0.999947	lincRNA:chr18:35967933-35986564 reverse strand

A_55_P1993153	Smtnl1	1.534362	0.999947	ref|Mus musculus smoothelin-like 1 (Smtnl1), mRNA [NM_024230]
A_66_P127412	ENSMUST00000103468	1.538182	0.999947	ens|immunoglobulin heavy variable V11-2 [Source:MGI Symbol;Acc:MGI:4947968] [ENSMUST00000103468]
A_30_P01017854	chr8:15580278-15583962_R	1.54878	0.999947	lincRNA:chr8:15580278-15583962 reverse strand
A_66_P125565	4933427J07Rik	1.601034	0.999947	gb|Mus musculus adult male testis cDNA, RIKEN full-length enriched library, clone:4933427J07 product:unclassifiable, full insert sequence. [AK016956]
A_55_P2806415	Slc15a2	1.6082	0.999947	ref|Mus musculus solute carrier family 15 (H+/peptide transporter), member 2 (Slc15a2), transcript variant 1, mRNA [NM_021301]
A_55_P2559025	A_55_P2559025	1.616971	0.999947	Unknown
A_55_P2066440	Myh3	1.622681	0.999947	ref|Mus musculus myosin, heavy polypeptide 3, skeletal muscle, embryonic (Myh3), mRNA [NM_001099635]
A_66_P107972	Defb23	1.667107	0.999947	ref|Mus musculus defensin beta 23 (Defb23), mRNA [NM_001037933]
A_66_P110707	Lrcol1	1.689978	0.999947	ref|Mus musculus leucine rich colipase-like 1 (Lrcol1), mRNA [NM_001033459]
A_55_P2736904	Dcc	1.743391	0.999947	ref|Mus musculus deleted in colorectal carcinoma (Dcc), mRNA [NM_007831]
A_66_P111041	Epha3	1.864984	0.999947	gb|Mus musculus 3 days neonate thymus cDNA, RIKEN full-length enriched library, clone:A630047N16 product:Eph receptor A3, full insert sequence. [AK041935]
A_55_P1972172	Cacng2	1.864997	0.999947	ref|Mus musculus calcium channel, voltage-dependent, gamma subunit 2 (Cacng2), mRNA [NM_007583]
A_55_P2580971	Gm32450	1.890275	0.999947	ref|PREDICTED: Mus musculus predicted gene, 32450 (Gm32450), transcript variant X4, ncRNA [XR_873945]
A_52_P868199	AK085542	1.943438	0.999947	gb|Mus musculus 0 day neonate kidney cDNA, RIKEN full-length enriched library, clone:D630039F19 product:unclassifiable, full insert sequence. [AK085542]
A_51_P436263	Olfr790	1.944415	0.999947	ref|Mus musculus olfactory receptor 790 (Olfr790), mRNA [NM_146933]
A_55_P2744634	Myh2	2.087109	0.999947	ref|Mus musculus myosin, heavy polypeptide 2, skeletal muscle, adult (Myh2), mRNA [NM_001039545]
A_55_P2735220	Gm3764	2.131491	0.999947	gb|Mus musculus adult male hippocampus cDNA, RIKEN full-length enriched library, clone:C630029I11 product:unclassifiable, full insert sequence. [AK141565]
A_51_P133684	Csrp3	2.233429	0.999947	ref|Mus musculus cysteine and glycine-rich protein 3 (Csrp3), transcript variant 1, mRNA [NM_013808]
A_65_P13823	Myoz2	2.304951	0.999947	ref|Mus musculus myozenin 2 (Myoz2), mRNA [NM_021503]
A_51_P248638	Myoz2	2.462825	0.999947	ref|Mus musculus myozenin 2 (Myoz2), mRNA [NM_021503]
A_51_P225134	Myh2	2.70279	0.999947	ref|Mus musculus myosin, heavy polypeptide 2, skeletal muscle, adult (Myh2), mRNA [NM_001039545]
A_51_P309740	Usp40	2.717635	0.999947	ref|Mus musculus ubiquitin specific peptidase 40 (Usp40), transcript variant 2, mRNA [NM_001033291]
A_55_P2105958	Trpa1	2.745334	0.999947	ref|Mus musculus transient receptor potential cation channel, subfamily A, member 1 (Trpa1), mRNA [NM_177781]
A_52_P175073	AK037317	2.998325	0.999947	gb|Mus musculus 16 days neonate thymus cDNA, RIKEN full-length enriched library, clone:A130006J09 product:NEPRILYSIN-LIKE PEPTIDASE GAMMA homolog [Mus musculus], full insert sequence. [AK037317]
A_66_P114091	Lvrn	3.939194	0.999947	ref|Mus musculus laeverin (Lvrn), mRNA [NM_029008]
